# Reductions in recurrence in women with early breast cancer entering clinical trials between 1990 and 2009: a pooled analysis of 155 746 women in 151 trials

**DOI:** 10.1016/S0140-6736(24)01745-8

**Published:** 2024-10-12

**Authors:** Early Breast Cancer Trialists' Collaborative Group

## Abstract

**Background:**

Distant recurrence in women with oestrogen receptor-positive early breast cancer persists at a constant rate for more than 20 years after diagnosis, with little equivalent data for oestrogen receptor-negative breast cancer. Using the database of the Early Breast Cancer Trialists’ Collaborative Group (EBCTCG) we investigated rates of distant breast-cancer recurrence in oestrogen receptor-positive and oestrogen receptor-negative tumours and trends in outcomes over time.

**Methods:**

In this pooled analysis of randomised controlled trial data, patients in the EBCTCG database of more than 650 000 women in trials of treatment for early-stage breast cancer were screened for eligibility. Women were eligible if they were enrolled between 1990 and 2009 and newly diagnosed with oestrogen receptor-positive breast cancer and scheduled for at least 5 years of endocrine therapy, or oestrogen receptor-negative disease, and if they were younger than 75 years at diagnosis, had a tumour diameter of 50 mm or less, and fewer than ten positive axillary lymph nodes, and no evidence of distant metastases at entry. Trial of neoadjuvant therapy, or those in which adjuvant therapy was unclear, and women with oestrogen receptor-negative, progesterone receptor-positive disease, or those for whom outcome or baseline data were missing were excluded. The primary outcome was time to first distant recurrence as defined by each trial, ignoring any locoregional recurrence or contralateral breast cancer. 10-year risks of distant recurrence by period of diagnosis were compared using Cox regression adjusted for patient and tumour characteristics, trial, and assigned treatment.

**Findings:**

Of the 652 258 women with early breast cancer in the EBCTCG database on Jan 17, 2023, patient-level data were available from 151 randomised trials that included 155 746 women. Rates of distant tumour recurrence improved similarly in women with oestrogen receptor-positive and oestrogen receptor-negative tumours. 80·5% of the improvement for oestrogen receptor-positive disease and 89·8% of the improvement for eostrogen receptor-negative disease was explained by changes in patient and tumour characteristics and improved treatments, but remained significant (p<0·0001). More recently diagnosed patients were more likely to have node-negative disease. 10-year distant recurrence risks during 1990–99 versus 2000–09 were as follows: for node-negative disease, 10·1% versus 7·3% for oestrogen receptor-positive disease and 18·3% versus 11·9% for oestrogen receptor-negative disease; for disease with one to three positive nodes, 19·9% versus 14·7% for oestrogen receptor-positive disease and 31·9% versus 22·1% for oestrogen receptor-negative disease; and for disease with four to nine positive nodes, 39·6% versus 28·5% for oestrogen receptor-positive disease and 47·8% versus 36·5% for oestrogen receptor-negative disease. After adjustment for therapy, rates were reduced by 25% (oestrogen receptor-positive disease) and 19% (oestrogen receptor-negative disease) after 2000 versus the 1990s, with similar improvements observed in oestrogen receptor-positive disease beyond 5 years.

**Interpretation:**

Most of the improvement in trial outcomes is explained by a greater proportion of women with lower-risk disease entering trials and improved adjuvant treatment. After adjustment, women diagnosed since 2000 have about a fifth lower rate of distant recurrence than the 1990s. Long-term risks of distant recurrence for oestrogen receptor-positive disease remain, but are about a tenth lower now than in our previous report.

**Funding:**

Cancer Research UK, UK Medical Research Council.

## Introduction

Adjuvant treatment for early-stage breast cancer substantially reduces the risk of recurrence compared with no treatment. For women with hormone receptor-positive breast cancer, 5 years of endocrine therapy with either tamoxifen or an aromatase inhibitor reduces the rate of distant recurrence and breast cancer death by about 40%.^1^, ^2^ However, previous work from the Early Breast Cancer Trialists’ Collaborative Group (EBCTCG) identified that, among women with oestrogen receptor-positive breast cancer who were disease-free after 5 years of scheduled endocrine therapy, risks of distant recurrence and death persisted at a relatively constant rate for at least the subsequent 15 years.^3^ Recurrence risk was strongly related to tumour stage (lymph node status and tumour size). For example, in women with T1 disease (tumour diameter ≤20 mm), the cumulative 20-year risk of distant recurrence was 13% with no axillary nodal involvement, 20% with one to three nodes involved, and 34% with four to nine nodes; in those with T2 disease (diameter 21–50 mm), the risks were 19% with no axillary nodal involvement, 26% with one to three nodes involved, and 41% with four to nine nodes.^3^ The pattern of long-term recurrence risk was confirmed in population-based data from the Danish Breast Cancer Group, which found that risks persisted for up to 32 years after diagnosis.^4^

**Research in context**

**Evidence before this study**

Reports by us and others have demonstrated that the risk of recurrence in women with hormone receptor-positive early breast cancer persists for at least two decades after diagnosis. However, these data were derived from trials conducted over many decades, and a recent analysis of a nationwide dataset suggested that outcomes for patients with early breast cancer have improved over time. Thus, estimates of recurrence on the basis of older datasets for women with early breast cancer, regardless of hormone receptor status, might not be relevant in today's practice. There is similarly a scarcity of information on women with hormone receptor-negative breast cancer, both in terms of outcomes and the extent to which they have improved over time. In both groups there is a need to understand the pattern of recurrence and whether the rates, or timing of recurrence, have changed with the advent of more modern therapies.

**Added value of this study**

Trial outcomes have improved over time in both hormone receptor-positive and hormone receptor-negative cancers. Most (80–90%) of this improvement can be explained by a combination of two factors: greater recruitment of women at lower risk in modern trials; and improved adjuvant therapies. Compared with 1990–99, in similar patients, adjusted for therapy, the rate of distant recurrence is reduced by approximately a quarter for oestrogen receptor-positive breast cancer and a fifth for oestrogen receptor-negative breast cancer. Although the risk of distant recurrence is lower in recent, compared to past eras, the risk of late recurrence is still present for patients with hormone receptor-positive breast cancer. By contrast, although risk of recurrence is lower for modern compared to older eras in hormone receptor-negative breast cancer, most of the recurrences in such patients continue to occur in the first 5 years after diagnosis.

**Implications of all the available evidence**

Estimates of recurrence rates can be used both by patients and their doctors in deciding on a course of therapy, in terms of balancing benefits and harms of therapies, and especially regarding whether to persist with endocrine therapy beyond 5 years in hormone receptor-positive disease, as well as in the design of future clinical trials. It is important to use up-to-date data and incorporate patient risk factors rather than simply estimating recurrence rates using overall results from older trials that reflect neither disease heterogeneity nor current case mix and outcomes.

Assessment of long-term outcomes is necessarily based on women diagnosed and treated many years, or even decades ago. For example, the women in the previous meta-analysis were diagnosed between 1976 and 2011.^3^ Improvements in care mean that their outcomes might not be representative of patients diagnosed today, and historical data might overestimate the risk of recurrence. Such overestimates could have implications for current patients and clinicians when balancing risks of recurrence and breast cancer mortality with toxicities and treatment-associated morbidities. Overestimating recurrence risks could also affect clinical trial design. Where prespecified analyses are event driven, some trials will require extended periods of follow-up (compared with the original plan in the protocol) to obtain sufficient power for analysis. This effect was observed in the recently reported TAILORx^5^ and RxPONDER trials,^6^ in which the observed event rate was lower than that anticipated using data from older studies.

Understanding patterns in recurrence by year of diagnosis, and considering reasons for any changes, can thus inform clinical practice and trial design. We evaluated outcome trends by period of diagnosis using individual patient-level data from participants in randomised trials within the EBCTCG database. This investigation extends our previous analysis for women with oestrogen receptor-positive disease receiving endocrine therapy,^3^ and widens the scope of the investigation to include women with oestrogen receptor-negative disease for whom endocrine therapy is ineffective.

## Methods

### Patients

In this pooled analysis of randomised trial data, patients in the EBCTCG database of more than 650 000 women in trials of treatment for early-stage breast cancer were screened for eligibility. Methods of trial identification and data collection are described in previous EBCTCG reports.^1^, ^2^, ^7^ Newly diagnosed women were included if they were enrolled between 1990 and 2009 and had no distant metastases at trial entry. In line with our previous report,^3^ women aged 75 years or older, with tumour diameter greater than 50 mm, or ten or more involved axillary nodes were excluded, as such women are rarely enrolled in trials and therefore might not be representative. Trials of neoadjuvant therapy were excluded because of the potential effect on pathological stage and hormone-receptor status. Women with oestrogen receptor-negative, progesterone receptor-positive breast cancer were also excluded. To reflect current treatment patterns, women with oestrogen receptor-positive disease were required to be scheduled for at least 5 years of adjuvant endocrine therapy. Finally, the women for whom adjuvant therapy allocation was unclear were excluded.

### Primary outcome and patient characteristics

The primary outcome was time to first distant recurrence as defined by each trial, ignoring any locoregional recurrence or contralateral breast cancer. Data were collected on tumour diameter and pathological nodal status (TN), tumour grade (low, moderate, or high), oestrogen receptor, progesterone receptor, and human epidermal growth factor receptor type 2 (HER2) status, as well as patient age, and intended adjuvant treatment as determined within each trial and recorded in the central EBCTCG database. TNM staging was used. Tumour diameter was categorised as 10 mm or less (T1a–b), 11–20 mm (T1c), 21–30 mm (T2), and 31–50 mm (T2); nodal status as no involved axillary nodes (N0), one to three positive nodes (N1–3), or four to nine positive nodes (N4–9). Assays and cutoff points to distinguish positive from negative oestrogen receptor, progesterone receptor, and HER2 tumours were determined by the participating trialists, according to standards established at time of diagnosis. Age was categorised as younger than 35 years, and then every 5 years from age 35–74 years. Missing data (eg, HER2, surgery, or grade) were treated as a separate category in analyses. Intended treatment according to protocol was collected as binary variables for surgery type (breast-conserving surgery or mastectomy), chemotherapy, including anthracycline with or without taxane use, HER2-directed therapy (for patients with HER2-positive disease), duration of endocrine therapy, and use of aromatase inhibitors for women with oestrogen receptor-positive disease. There was insufficient information provided on the use of radiotherapy for these data to be included.

### Statistical analysis

Analyses of distant recurrence at any time were censored at 10 years to allow an equivalent follow-up period across each enrolment period. Rates of distant recurrence were compared by period of diagnosis, both as a continuous variable, and split into periods: 1990–99; 2000–04; and 2005–09. These periods were chosen to reflect changes in treatment patterns and to ensure sufficient distant recurrences for analysis in each time period.

Analyses were done separately for oestrogen receptor-positive and oestrogen receptor-negative tumours. Changes in patient characteristics over time were assessed using linear and logistic regression. Rates of distant recurrence in each period of diagnosis were displayed using the Kaplan–Meier method, as in previous EBCTCG meta-analyses.^7^ Hazard rate ratios (RRs) for distant recurrence by period of diagnosis were calculated using Cox regression adjusted for the patient, tumour, and treatment variables previously described. The strength of evidence for improvements over time was assessed using the deviance statistic, giving a χ^2^ for trend over period of diagnosis. The degree to which improvements in time could be explained by other characteristics was assessed by looking at the reduction in deviance after adjusting for patient, tumour, and treatment characteristics: a reduction of 50% in the deviance between an unadjusted and adjusted model implies that half of the apparent improvement can be explained by the variables being adjusted for. RRs by period of diagnosis with 95% CIs are displayed using the method of floating absolute risks to avoid reliance on a reference group that itself is subject to variability.^8^ All p values are two-sided.

### Role of the funding source

The funders had no role in study design, data collection, data analysis, data interpretation, or writing of this report.

## Results

**Figure 1:**
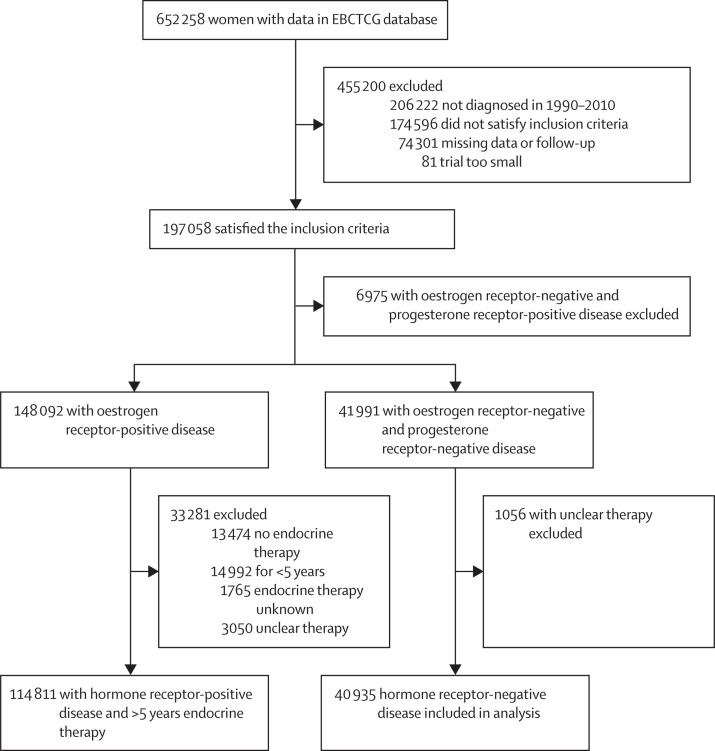
Selection of patients for the analysis of trends in outcome over time Patients were required to be younger than 75 years, with a tumour diameter of less than 50 mm, with fewer than ten positive nodes, and no known distant metastases at entry. EBCTCG=Early Breast Cancer Trialists’ Collaborative Group.

Of the 652 258 women with early breast cancer in the EBCTCG database on Jan 17, 2023, 155 746 women from 151 trials (table 1; appendix pp 11–34) satisfied the inclusion criteria: 114 811 women with oestrogen receptor-positive disease scheduled to receive at least 5 years of endocrine therapy and 40 935 women with oestrogen receptor-negative disease (figure 1).

**Table 1:** Characteristics of 114 811 women with oestrogen receptor-positive disease scheduled to receive 5 years or more of endocrine therapy and 40 935 women with oestrogen receptor-negative disease

	**Oestrogen receptor-positive disease**				**Oestrogen receptor-negative disease**			
	Overall (N=114 811)	1990–99 (N=29 080)	2000–04 (N=45 495)	2005–09 (N=40 236)	Overall (N=40 935)	1990–99 (N=15 295)	2000–04 (N=16 180)	2005–09 (N=8830)
**Age at diagnosis**								
<35 years	3036 (2·6%)	640 (2·2%)	1318 (2·9%)	1078 (2·7%)	2520 (6·2%)	1113 (7·0%)	958 (5·9%)	449 (5·1%)
35–44 years	17 962 (15·6%)	3560 (12·2%)	7351 (16·2%)	7051 (17·5%)	9769 (23·9%)	4044 (25·4%)	3787 (23·4%)	1938 (22·0%)
45–54 years	35 997 (31·4%)	8408 (28·9%)	14 712 (32·3%)	12 877 (32·0%)	13 898 (34·0%)	5210 (32·7%)	5866 (36·3%)	2822 (32·0%)
55–64 years	37 214 (32·4%)	10 024 (34·5%)	14 665 (32·2%)	12 525 (31·1%)	10 857 (26·5%)	3903 (24·5%)	4291 (26·5%)	2663 (30·2%)
65–74 years	20 602 (17·9%)	6448 (22·2%)	7449 (16·4%)	6705 (16·7%)	3891 (9·5%)	1655 (10·4%)	1278 (7·9%)	958 (10·9%)
Mean difference per decade	..		−0·17 (−0·18 to −0·16); p<0·0001		..		0·09 (0·07 to 0·11); p<0·0001	
**Tumour diameter, mm**								
1–10 (T1a–b)	20 100 (17·5%)	3568 (12·3%)	7917 (17·4%)	8615 (21·4%)	4135 (10·1%)	1582 (9·9%)	1842 (11·4%)	711 (8·1%)
11–20 (T1c)	47 433 (41·3%)	12 874 (44·3%)	18 534 (40·7%)	16 025 (39·8%)	15 028 (36·7%)	5924 (37·2%)	5569 (34·4%)	3535 (40·0%)
21–30 (T2)	29 766 (25·9%)	8290 (28·5%)	11 685 (25·7%)	9791 (24·3%)	13 074 (31·9%)	4916 (30·9%)	5153 (31·9%)	3005 (34·0%)
31–50 (T2)	17 512 (15·3%)	4348 (15·0%)	7359 (16·2%)	5805 (14·4%)	8698 (21·3%)	3503 (22·0%)	3616 (22·4%)	1579 (17·9%)
Odds ratio per category by time period	..		0·86 (0·85 to 0·88); p<0·0001		..		0·97 (0·95 to 1·00); p=0·024	
**Axillary nodal status**								
Zero positive nodes	49 735 (43·3%)	10 673 (36·7%)	17 465 (38·4%)	21 597 (53·7%)	17 784 (43·4%)	6841 (43·0%)	6025 (37·2%)	4918 (55·7%)
One to three positive nodes	46 412 (40·4%)	12 285 (42·3%)	19 328 (42·5%)	14 799 (36·8%)	14 967 (36·6%)	5329 (33·5%)	6570 (0·6%)	3068 (34·8%)
Four to nine positive nodes	18 664 (16·3%)	61 222 (21·1%)	8702 (19·1%)	3840 (9·5%)	8184 (20·0%)	3755 (23·6%)	3585 (22·2%)	844 (9·6%)
Odds ratio per category by time period	..		0·68 (0·67 to 0·69); p<0·0001		..		0·77 (0·75 to 0·79); p<0·0001	
**Tumour grade (differentiation)**								
Low (well differentiated)	14 000 (12·2%)	3625 (12·5%)	5923 (13·0%)	4452 (11·1%)	661 (1·6%)	391 (2·5%)	207 (1·3%)	63 (0·7%)
Moderate	46 903 (40·9%)	10 924 (37·6%)	19 207 (42·2%)	16 772 (41·7%)	6257 (15·3%)	2707 (17·0%)	2540 (15·7%)	1010 (11·4%)
High (poorly differentiated)	25 947 (22·6%)	5694 (19·6%)	11 885 (26·1%)	8368 (20·8%)	23 257 (56·8%)	6930 (43·5%)	10 474 (64·7%)	5853 (66·3%)
Unknown grade	27 961 (24·4%)	8837 (30·4%)	8480 (18·6%)	10 644 (26·5%)	10 760 (26·3%)	5897 (37·0%)	2959 (18·3%)	1904 (21·6%)
Odds ratio per category by time period*	..		1·05 (1·03 to 1·06); p=0·0001		..		1·71 (1·66 to 1·75); p<0·0001	
**Progesterone receptor status (oestrogen-receptor positive)**								
Oestrogen receptor-positive, progesterone-poor disease	18 255 (15·9%)	5253 (18·1%)	7892 (17·4%)	5110 (12·7%)	..	..	..	..
Oestrogen receptor-positive, progesterone-positive disease	96 556 (84·1%)	23 827 (81·9%)	37 603 (82·6%)	35 126 (87·3%)	..	..	..	..
Odds ratio by time period	..		1·19 (1·16 to 1·21); p<0·0001		..	..	..	..
**HER2 overexpression**								
HER2 negative	52 341 (45·6%)	6320 (21·7%)	21 060 (46·3%)	24 961 (62·0%)	13 179 (32·2%)	2009 (12·6%)	5888 (36·4%)	5282 (59·8%)
HER2 positive	13 018 (11·3%)	1189 (4·1%)	8723 (19·2%)	3106 (7·7%)	8945 (21·9%)	929 (5·8%)	6652 (41·1%)	1364 (15·5%)
HER2 unknown	49 452 (43·1%)	21 571 (74·2%)	15 712 (34·5%)	12 169 (30·2%)	18 811 (46·0%)	12 987 (81·6%)	3640 (22·5%)	2184 (24·7%)
Odds ratio for HER2 testing by time period	..		2·46 (2·42 to 2·50); p<0·0001		..		4·77 (4·61 to 4·94); p<0·0001	
p value for HER2 positivity by period	..		p<0·0001		..		p<0·0001	
**Original breast surgery**								
Breast conserving	64 919 (56·5%)	13 992 (48·1%)	24 415 (53·7%)	26 512 (65·9%)	21 014 (51·3%)	7324 (46·0%)	8148 (50·4%)	5542 (62·8%)
Mastectomy	47 184 (41·1%)	13 903 (47·8%)	19 959 (43·9%)	13 322 (33·1%)	18 326 (44·8%)	7930 (49·8%)	7346 (45·4%)	3050 (34·5%)
Unknown	2708 (2·4%)	1185 (4·1%)	1121 (2·5%)	402 (1·0%)	1595 (3·9%)	671 (4·2%)	686 (4·2%)	238 (2·7%)
Odds ratio per category by time period*	..		0·71 (0·70 to 0·71); p<0·0001		..		0·73 (0·71 to 0·75); p<0·0001	
**Chemotherapy scheduled**								
Yes	75 414 (65·7%)	17 329 (59·6%)	30 910 (67·9%)	27 175 (67·5%)	37 518 (91·7%)	13 265 (83·3%)	15 473 (95·6%)	8780 (99·4%)
Non-anthracycline, non-taxane chemotherapy	9361 (8·2%)	1845 (6·3%)	3244 (7·1%)	4272 (10·6%)	6181 (15·1%)	3672 (23·1%)	2056 (12·7%)	453 (5·1%)
Anthracycline	22 513 (19·6%)	10 200 (35·1%)	6881 (15·1%)	5432 (13·5%)	12 964 (31·7%)	7257 (45·6%)	2879 (17·8%)	2828 (32·0%)
Taxane	3197 (2·8%)	332 (1·1%)	976 (2·2%)	1889 (4·7%)	1908 (4·7%)	128 (0·8%)	1029 (6·4%)	751 (8·5%)
Anthracycline + taxane	40 343 (35·1%)	4952 (17·0%)	19 809 (43·5%)	15 582 (38·7%)	16 465 (40·2%)	2208 (13·9%)	9509 (58·8%)	4748 (53·8%)
No	39 397 (34·3%)	11 751 (40·4%)	14 585 (32·1%)	13 061 (32·5%)	3417 (8·3%)	2660 (16·7%)	707 (4·4%)	50 (0·6%)
Odds ratio for chemotherapy by time period	..		1·18 (1·16 to 1·20); p<0·0001		..		4·82 (4·48 to 5·18); p<0·0001	
Odds ratio for anthracycline by time period	..		0·99 (0·97 to 1·00); p=0·059		..		2·08 (2·02 to 2·15); p<0·0001	
Odds ratio for taxane by time period	..		1·62 (1·60 to 1·65); p<0·0001		..		3·22 (3·12 to 3·32); p<0·0001	
**Trastuzumab scheduled in HER2-positive disease**								
Yes	6139 (46·6%)	0	4380 (49·6%)	1759 (55·5%)	4672 (51·9%)	0	3812 (56·8%)	860 (63·1%)
No	7048 (53·4%)	1189 (100%)	4448 (50·4%)	1411 (44·5%)	4329 (48·1%)	929 (100%)	2896 (43·2%)	504 (37·0%)
Odds ratio between 2000–04 and 2005–09	..		1·27 (1·17 to 1·37); p<0·0001		..		1·30 (1·15 to 1·46); p<0·0001	
**Aromatase inhibitors given (oestrogen receptor-positive disease)**								
Yes	47 165 (41·1%)	4250 (14·6%)	17 332 (38·1%)	25 583 (63·6%)	..	..	..	..
No	67 646 (58·9%)	24 830 (85·4%)	28 163 (61·9%)	14 653 (36·4%)	..	..	..	..
Odds ratio by time period	..		2·84 (2·76 to 2·92); p<0·0001		..	..	..	..
**Duration of endocrine therapy (oestrogen receptor-positive disease)**								
5 years	96 686 (84·2%)	29 080 (100%)	41 655 (91·6%)	25 951 (64·5%)	..	..	..	..
>5 years	18 125 (15·8%)	0	3840 (8·4%)	14 285 (35·5%)	..	..	..	..
Odds ratio between 2000–04 and 2005–09	..		5·97 (5·74 to 6·21); p<0·0001		..	..	..	..

Odds ratios and p values are for comparisons between all time periods.

* Excluding unknown.

Women with oestrogen receptor-positive disease were older than those with oestrogen receptor-negative disease (median age 55 years, IQR 47–62, *vs* 50 years, 43–58); 29 080 (25·3%) of women with oestrogen receptor-postive disease and 15 295 (37·4%; table 1) of those with oestrogen receptor-negative disease were diagnosed before 2000. Women with oestrogen receptor-positive disease were more likely to have smaller tumours (T1 67 533 [58·8%] *vs* 19 163 [46·8%]); 67 519 (43·3%) had N0 disease. Data were available regarding tumour grade in 117 025 (75·1%) of women, and HER2 status in 87 483 (56·2%) of women. As expected, tumour grade was lower for oestrogen receptor-positive than oestrogen receptor-negative disease; oestrogen receptor-positive disease was more likely to be HER2-negative (table 1). Breast-conserving surgery was done in 85 933 (55·2%) of women. Among women with oestrogen receptor-positive disease, 47 165 (41·1%) were assigned an aromatase inhibitor, either instead of, in combination, or sequentially with tamoxifen.

Analyses of tumour characteristics by year of diagnosis showed a significant trend towards lower risk tumours in more recent years, driven by a highly significant trend towards lower nodal status (p<0·0001 for both oestrogen receptor-positive and oestrogen receptor-negative groups; table 1; appendix p 35). There was strong evidence of a trend towards smaller tumours in the oestrogen receptor-positive group over time (table 1; appendix p 35). Trends in participant age were different between the two groups with an increase in women with oestrogen receptor-positive tumours younger than 50 years since 2000 versus more women aged 50–69 years with oestrogen receptor-negative disease in the most recent cohort (p<0·0001 in both oestrogen receptor-positive and oestrogen receptor-negative groups; table 1; appendix p 36); in both cases few women older than 70 years were enrolled. The distribution of women with HER2-positive cancers show the effects of more HER2 testing, and changes in trial eligibility over time; the greatest number of women with HER2-positive tumours were included in 2000–04, when HER2-directed therapies were being tested. More recent trials recruited fewer women with HER2-positive disease as, more frequently, eligibility criteria excluded such women (table 1; appendix p 36).

**Figure 2:**
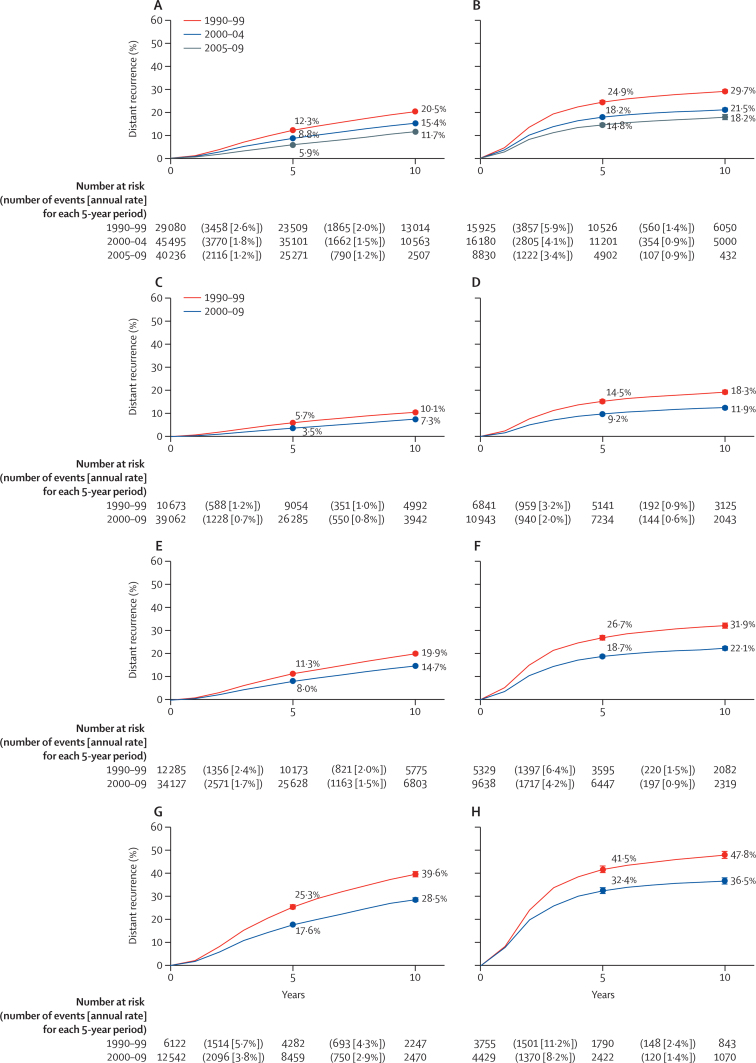
Risk of distant recurrence by period of enrolment Risk of distant recurrence in patients with oestrogen receptor-positive disease (A); oestrogen receptor-negative disease (B); node-negative, oestrogen receptor-positive disease (C); node-negative, oestrogen receptor-negative disease (D); one-to-three positive nodes and oestrogen receptor-positive disease (E); one-to-three positive nodes and oestrogen receptor-negative disease (F); four-to-nine positive nodes and oestrogen receptor-positive disease (G); four-to-nine positive nodes and oestrogen receptor-negative disease (H). pinteraction<0·0001 between oestrogen receptor-positive and oestrogen receptor-negative disease (A, B). (C–H) Periods 2000–04 and 2005–09 combined to reflect the similarity of outcomes in those periods (for graphs for three time periods see appendix pp 37–38).

Unadjusted rates of distant recurrence in women by oestrogen receptor status and period of enrolment were calculated (figure 2A, B). The risk of distant recurrence was significantly reduced in each successive time period (p<0·0001 for both groups). Cox regression by year of diagnosis yielded an RR of 0·939 (95% CI 0·936– 0·942) per calendar year in women with oestrogen receptor-positive tumours and 0·963 (0·960–0·967) in the oestrogen receptor-negative group; outcomes improved more rapidly in women with oestrogen receptor-positive tumours (p_interaction_<0·0001; figure 2). The shapes of the recurrence curves differed; the oestrogen receptor-positive group showed that a roughly constant annual risk of recurrence persisted even in more recently diagnosed patients, whereas about 80% of the recurrence risk for oestrogen receptor-negative tumours occured in the first 5 years after diagnosis, regardless of time period.

**Figure 3:**
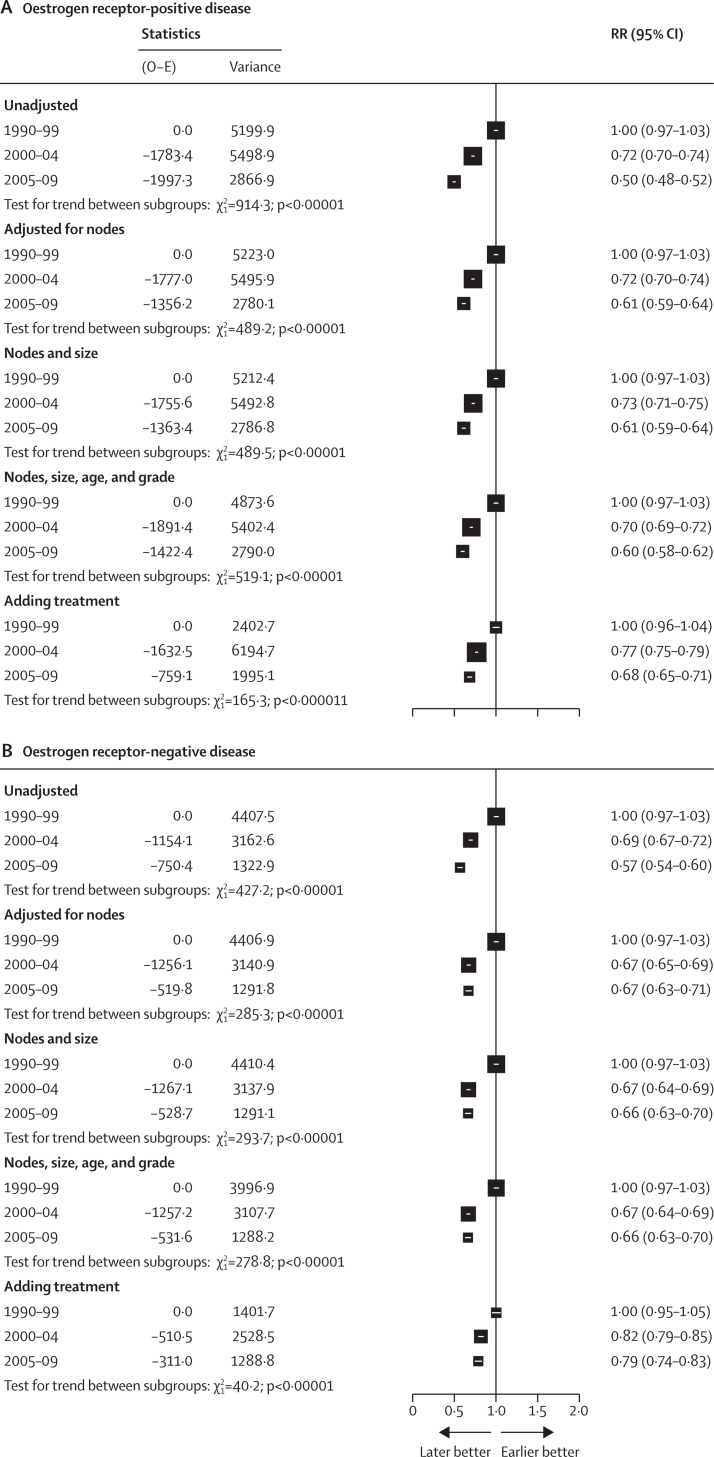
Association between year of diagnosis and rate ratio for distant recurrence during years 0–9 from enrolment, adjusted successively for tumour, patient characteristics, and treatment

Given the clear evidence of association between patient and tumour characteristics and period of recruitment, further analyses focused on understanding whether improvements in outcomes were associated with changes in case mix, either because of diagnosis at earlier stages or changes in trial inclusion criteria. The clearest evidence of change in patient characteristics comes from the association between year of enrolment and nodal status. Stratifying analyses by nodal status attenuated the effect of recruitment period as women diagnosed more recently had fewer positive nodes (and hence a better prognosis). In particular, there was little evidence of differences in outcomes in women diagnosed in 2000–04 and 2005–09, and so results for these two periods were combined for display purposes (figure 3; appendix pp 37–38). Similar trends were seen in analyses stratified by TN status (appendix p 39). The RRs by period of enrolment were closer to 1·0 after adjustment (figure 3). The χ^2^ deviance statistic in the adjusted Cox regression was reduced by about a half in the oestrogen receptor-positive group and a third in the oestrogen receptor-negative group (table 2). This decrease represents the proportion of the apparent improvement explained by changes in TN status. Other univariate adjustments made little difference to the deviance, indicating that these factors do not explain improving outcomes, with the exception of age. More recent trials among women with oestrogen receptor-positive disease include a greater proportion of younger women, who have a higher underlying risk of recurrence, and so adjusting for age increases the improvement over time.

**Table 2:** Effect of successive adjustment on deviance associated with period of enrolment in Cox regression analyses

		**Oestrogen receptor-positive disease**	**Oestrogen receptor-negative disease**
Unadjusted		940·65	433·05
Univariate adjustment			
	Nodes alone	481·25 (48·8)	282·45 (34·8)
	Size alone	831·41 (11·6)	426·80 (1·4)
	Grade alone	942·70 (−0·2)	420·11 (3·0)
	Age alone	1037·28 (−10·3)	422·61 (2·4)
Bivariate adjustment			
	Nodes and size	482·40 (48·7)	290·73 (32·9)
	Nodes and grade	508·80 (45·9)	274·76 (36·6)
	Nodes and age	503·37 (46·5)	276·01 (36·3)
	Size and grade	833·15 (11·4)	426·86 (1·4)
	Size and age	892·38 (5·1)	419·88 (3·0)
	Grade and age	1007·64 (−7·1)	429·52 (0·8)
Adjustment for patient and tumour characteristics			
	Nodes, size, grade, and age	510·18 (45·8)	276·00 (36·3)
Addition of treatment variables			
	Including chemotherapy	316·14 (66·4)	59·90 (86·2)
	Including chemotherapy and biological treatment	278·78 (70·4)	44·17 (89·8)
	Including chemotherapy, biological treatment, and hormonal treatment (oestrogen receptor-positive disease)	182·98 (80·5)	..

Deviance associated with period of enrolment (percentage reduction from unadjusted analyses)

Adjusting for nodal status, tumour size, age, and grade reduced the deviance by 430·47 (45·8%) in the oestrogen receptor-positive group and 157·05 (36·3%) in the oestrogen receptor-negative group, suggesting that a significant proportion of the improvement seen here for outcomes for patients enrolled in clinical trials can be explained by changes in the population of women in these trials, especially since 2005 (table 2). Compared with 1990–99, after adjusting for patient and tumour characteristics, the RR for 2005–09 was 0·60 (95% CI 0·58–0·62) for women with oestrogen receptor-positive tumours and 0·66 (0·63–0·70) for those with oestrogen receptor-negative disease (figure 3A,B).

After adjusting for patient and tumour characteristics, there remained a substantial degree of unexplained improvement in distant recurrence rates by time period. To determine the extent to which more efficacious modern therapies might influence these improvements, analyses were adjusted successively by use and type of chemotherapy (including anthracycline and taxane use), trastuzumab for those with HER2-positive disease, and, for the oestrogen receptor-positive group, hormonal therapy (including duration and the use of aromatase inhibitors). Variables were introduced based upon the planned treatment protocol within each trial; in adjusted analyses, the deviance indicated that for oestrogen receptor-positive women, 80·5% of the improvement in outcomes over time can be explained by changes in patient population and therapy; with 89·8% of the improvement in outcomes over time explainable by these factors in oestrogen receptor-negative disease (table 2). Between a third and a half of this improvement can be attributable to changes in therapy alone. Again, RRs for time periods after 2000 were closer to 1·0, reflecting an association between more modern therapies and lower rates of recurrence (rate ratio for 2005–09 *vs* 1990–99 was 0·68, 95% CI 0·65–0·71 for oestrogen receptor-positive tumours and 0·79, 0·74–0·83 for oestrogen receptor-negative tumours; figure 3A, B).

To further understand the effect of changes in patient management, we did two sensitivity analyses. Recognising that the management of women with HER2-positive tumours changed substantially between 1990 and 2009, both in terms of HER2 testing and use of trastuzumab,^9^ we looked separately by HER2 status. To reflect the differences in testing rates, we considered both those without known HER2-positive disease (ie, HER2-negative and HER2-unknown disease), and those with documented HER2-negative disease. Results were not materially different in either sensitivity analysis (appendix pp 42–45). An analysis of HER2-positive tumours reflected improvements in outcomes following the introduction of trastuzumab (appendix p 45).

**Figure 4:**
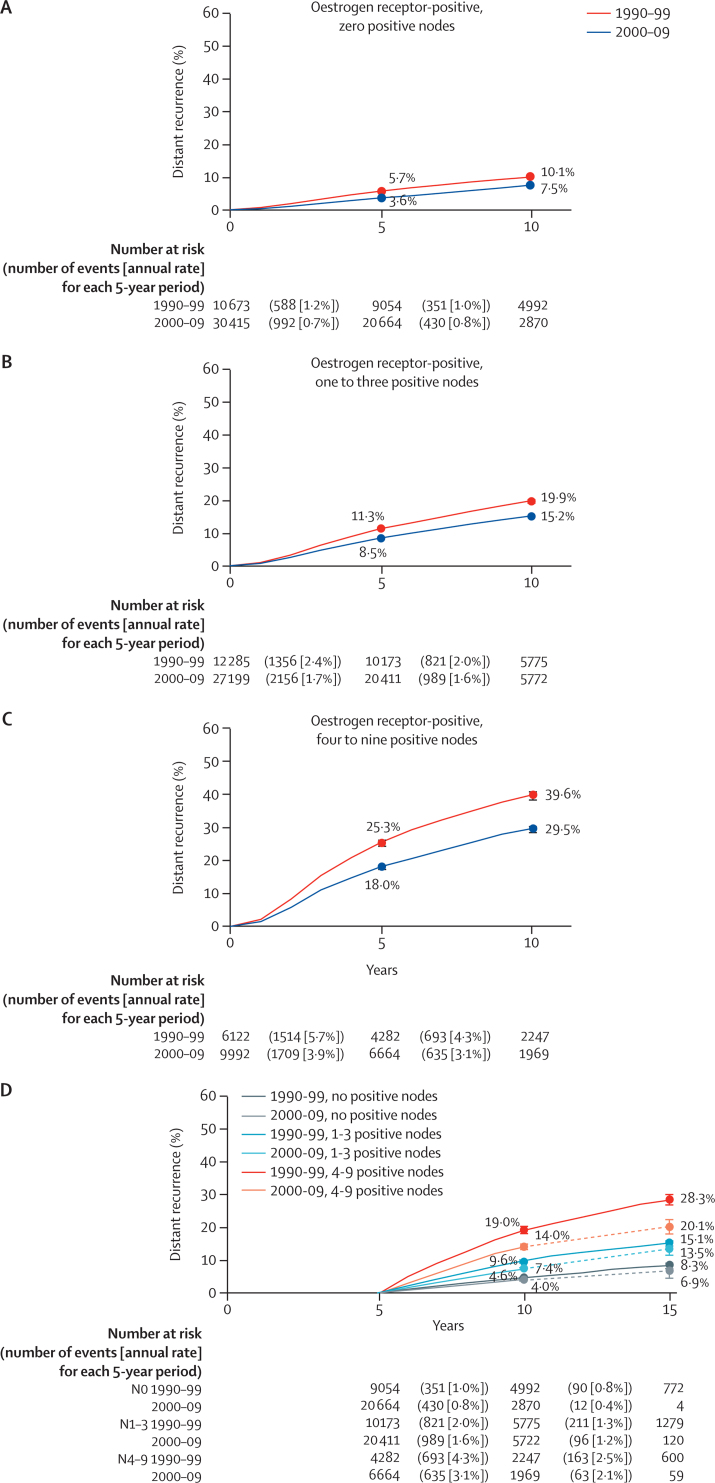
Distant recurrence by nodal status for women with oestrogen-receptor-positive disease with 5 years of scheduled endocrine therapy (A) Years 0–9 from diagnosis, node-negative disease. (B) Years 0–9 from diagnosis, one to three positive nodes. (C) Years 0–9 from diagnosis, four to nine positive nodes; (D) Recurrence after year 5 for women who were alive and recurrence free at 5 years split by period of diagnosis and nodal status; data beyond 10 years is smoothed for patients diagnosed after 2000 (dotted lines).

Additionally, we analysed the group of women with oestrogen receptor-positive disease scheduled for only 5 years of endocrine therapy to address explicitly the issue of whether improvement could be ascribed to a switch to longer-term endocrine therapy. Results remained consistent with those in the entire population with a similar pattern of lower, but still appreciable, distant recurrence in more recently diagnosed patients (figure 4A–D; appendix p 46).

## Discussion

In this study of women with early breast cancer enrolled in clinical randomised trials, we observed substantial reductions in 10-year risks of distant tumour recurrence for those diagnosed after 2000 compared with those diagnosed from 1990 to 1999, both in those with oestrogen receptor-positive tumours scheduled for at least 5 years of endocrine therapy, and oestrogen receptor-negative disease. Consistent with our earlier report,^3^ women allocated at least 5 years of endocrine therapy, who remained recurrence-free at year 5, remained at constant annual risk of distant recurrence in the succeeding 5 years (figure 4D). Our data suggest that for women with oestrogen receptor-positive disease diagnosed after 2000 who have at least 5 years of endocrine therapy planned, the risk of distant recurrence persists but is about a tenth lower than equivalent women in our previous report.^3^ By contrast, for those with oestrogen receptor-negative disease, the majority of recurrences occurred in the first 5 years after diagnosis.

The improvements in outcome in more recently diagnosed women have a variety of causes. Notably, the proportion of women with node-negative disease entering trials increased over time, accounting for a third to a half of the improvement in outcomes. This change coincided with the widespread application of mammographic screening and increased breast-cancer awareness. Earlier diagnosis and therapy might provide superior therapeutic results, but could also result in both lead-time and overdiagnosis bias.^10^, ^11^ The EBCTCG database does not include means of diagnosis (screened *vs* interval cancers), therefore this issue cannot be investigated here. However, detecting tumours at an earlier stage cannot be the sole factor as improvements persisted even after adjustment for tumour size and nodal involvement. Another possible explanation is more accurate tumour staging resulting from improvements in diagnostic accuracy. Detecting tumour spread more accurately can upstage tumours, leading to artefactual improvements in stage-stratified analyses, the so-called Will Rogers effect.^12^ The staging system for breast cancer underwent a substantial change in 2009;^13^ prior to that year, and particularly during the period of this study, staging approaches did not change substantially. Although we did not include staging per se in the analysis, it is possible that changes in methods for evaluating lymph node involvement over time (from dissection, sampling, and sentinel node biopsy) might have altered estimates of prognosis among different categories of axillary nodal status (figure 2). However, we were unable to analyse this within our dataset.

Methods of analysis of tumour biomarker tests, including oestrogen receptor and HER2, have also evolved over the past four decades. Oestrogen receptor testing has changed from ligand-binding assays (LBA) to immunohistochemical analysis, with varying analytical technologies and cutoffs.^14^, ^15^ These changes might artefactually alter estimates of outcomes both for women designated as having oestrogen receptor-positive and oestrogen receptor-negative breast cancers, given that rates of false positives and negatives are higher with LBA than immunohistochemistry.

Additionally, trial design and selection of participants might have changed over time as more specific questions have been addressed. In the 1980s and 1990s, poor-prognosis patients entered trials of anthracycline and taxane chemotherapy or high-dose chemotherapy with haematopoietic stem cell transplant, regardless of tumour receptor profile. By contrast, more recently, clinical trials addressing endocrine therapy questions with aromatase inhibitors enrolled only patients with oestrogen receptor-positive disease, often with earlier stage disease. In the early 2000s several trials focused on trastuzumab and only recruited patients with HER2-positive breast cancers. Taken together, these caveats emphasise the need for caution in relying on clinical trial reports to explore trends in patient outcomes. However, a recent study of real-world data reported remarkably similar findings to ours, with improvement in overall survival over time for patients with newly diagnosed breast cancer in England.^16^

To address confounding, we explored the influence of other possible reasons for improving prognosis in this dataset. In addition to changes in patient and tumour characteristics, between a third and a half of the improvement can be explained by changes in therapy. Recent meta-analyses have shown that breast cancer outcomes are improved with better adjuvant chemotherapy, such as combining anthracyclines and taxanes or more dose-intense regimens.^17^, ^18^ For patients with oestrogen receptor-positive cancers, more effective adjuvant endocrine therapies, such as aromatase inhibitors,^2^, ^19^ and, possibly, longer endocrine therapy duration,^20^, ^21^ have been introduced into standard care. Likewise, adjuvant anti-HER2 therapies, such as trastuzumab,^9^ have greatly improved outcomes for patients with HER2-positive disease. Prospective randomised clinical trials demonstrated significant reductions in bone recurrence from the use of bisphosphonates.^22^ This conclusion is supported by the observation that breast cancer mortality in the USA, and elsewhere, has steadily declined over the past three decades.^23^, ^24^ Overall, adjustment reduced the apparent improvement in recurrence rates in women diagnosed in 2005–09 compared with 1990–99 by about a half. Our study reflected current standards of care by excluding patients assigned to take endocrine therapy for less than 5 years. Had we included patients with shorter treatment, it is likely that the unadjusted improvements in outcomes for patients with oestrogen receptor-positive disease diagnosed in 2000–09 versus 1990–99 would have been even greater. More-recently introduced adjuvant treatments, such as immune checkpoint inhibitors, inhibitors of poly (ADP-ribose) polymerase, and novel anti-HER2 treatments, have the potential to further reduce distant tumour recurrence rates.

These data have direct implications. First, they can guide discussions between clinicians and patients on the use of adjuvant therapies in the modern era. Our results suggest that the absolute benefit of continuing endocrine therapy will be lower than that calculated from previous analyses, such as our previous report on oestrogen receptor-positive disease.^3^ If we assume that extended endocrine therapy beyond 5 years reduces the subsequent distant recurrence by approximately a quarter,^20^, ^21^ clinicians should use our updated risk estimates rather than our previous report^3^ to estimate the absolute potential benefit. A woman who had node-negative breast cancer and who discontinues adjuvant endocrine therapy after 5 years probably has an annual risk of approximately 0.9%, or about 4.5% over the period of 5–10 years after diagnosis (figure 2C). In this case, the estimated reduction of about a quarter with extended endocrine therapy will, at most, benefit about 1% of patients. This potential benefit must be considered alongside the known ongoing side-effects that affect quality of life and the potential risk of life-threatening toxicities, such as thrombosis or endometrial cancer with tamoxifen and osteoporosis and fracture with aromatase inhibitors (somewhat mitigated with the use of bisphosphonates).^2^, ^19^

However, despite improvements in outcomes, women with oestrogen receptor-positive breast cancer and four to nine positive nodes still have a substantial risk of late recurrence after 5 years of adjuvant endocrine therapy of about 2·7% per year, or about 13% over the period of 5–10 years after diagnosis. A 25% reduction from extended endocrine therapy would equate to about a 3% absolute reduction over this period (and more over a longer period of follow-up).

In addition to implications regarding routine patient care, the results of this analysis guide the design of future trials. Investigators must consider the expected case mix when planning trials, to obtain an accurate estimate of the control-group event rate. Our data suggest that, rather than using overall outcomes from previous trials, it would be preferable to model event rates using expected numbers of participants by oestrogen-receptor status and nodal status (figure 2C–H), and potentially by tumour size as well. Consequently, future trials might need to be larger than previously anticipated. Alternatively, clinical investigators might need to collect longer follow-up.

In summary, despite the limitations of using data derived only from participants in randomised trials, our data strongly suggest that long-term estimates of the annual risk of distant recurrence in women with early-stage breast cancer are lower for patients diagnosed since 2000 than in earlier decades. Although most of the recurrences for oestrogen receptor-negative cancers occur during the first 5 years, for those with oestrogen receptor-positive disease the risk persists at a constant rate beyond 5 years. However, when making absolute estimates of either distant recurrence or treatment effect, improved outcomes in the modern era must be accounted for.

Correspondence to: EBCTCG Secretariat, Clinical Trial Service Unit, Nuffield Department of Population Health, Oxford OX3 7LF, UK

### EBCTCG Secretariat

### Contributors

### Data sharing

Data in these analyses have been provided under licence from the original trialists who retain rights over the data. Please see the EBCTCG data sharing policy at https://www.ctsu.ox.ac.uk/research/the-early-breast-cancer-trialists-collaborative-group-ebctcg.

**This online publication has been corrected. The corrected version first appeared at thelancet.com on May 22, 2025**

## Declaration of interests

DFH reports support unrelated to this study but provided to his institution in the past 24 months during conduct and analysis of this study from Astra Zeneca, Menarini Silicon Biosystems, and Pfizer. DFH reports personal income related to consulting or advisory board activities from Artera AI, Arvinas, Biotheranostics an Hologic Company, BioVeca, Cellworks, Centrix, Cepheid, Delphi Diagnostics, EPIC Sciences, EXACT Sciences, Freenome, Guardant, L-Nutra, Macrogenics, Microbiologics, Oncocyte, Predictus BioSciences, Stratipath, Tempus, Turnstone Biologics, and Xilis. The University of Michigan holds a patent for which DFH is the named investigator and which was licensed to Menarini Silicon Biosystems from whom UM and DFH have received annual royalties, ceasing on Jan 1, 2021. DFH reports personally held stock options from InBiomotion, Cellworks, and Xilis. SMS reports personal income related to consulting or advisory board activities from AstraZeneca, Daiichi-Sankyo, Genentech/Hoffman LaRoche, Biotheranostics, Natera, and Sanofi; in-kind third party writing from AstraZeneca and Genentech Hoffman La Roche; in-kind travel from Genentech/Hoffman La Roche, Sanofi, and Daiichi-Sankyo; research to institution from Kailos Genetics and Genentech; and is a member of the board of directors of Seagen with stock, stock options, and stipend; and is on the scientific advisory board of and receives consulting fees from Napo Pharmaceuticals. The institutions of RDG receive partial support for his salary from Roche, AstraZeneca, and Merck. JB reports institutional funding from Amgen, AstraZeneca, Bayer, Merck, Pfizer, Roche, and Sanofi-Aventis; stock holding in Stratipath; is a Chairperson for Coronis and Asklepios Cancer Research; receives honoraria from Roche and AstraZeneca for chairmanship; and lectures at scientific meetings and consultations for Stratipath.
