# Supplementary material for: Reductions in recurrence in women with early breast cancer entering clinical trials between 1990 and 2009: a pooled analysis of 155 746 women in 151 trials
Source: Lancet. 2024 Oct 12;404(10461):1407–18. doi: 10.1016/S0140-6736(24)01745-8 (PMC12979726; doi:10.1016/S0140-6736(24)01745-8)

THE LANCET

Supplementary appendix

This appendix formed part of the original submission and has been peer reviewed. We post it as supplied by the authors.

Supplement to: Early Breast Cancer Trialists' Collaborative Group. Reductions in the recurrence in women with early breast cancer entering clinical trials between 1990 and 2009: a pooled analysis of 155 746 women in 151 trials. *Lancet* 2024; **404:** 1407–14.

Supplementary online material for:

Reductions in recurrence rates in women with early breast cancer entering clinical trials between 1990 and 2009: analyses of 155,746 women in 151 trials

Early Breast Cancer Trialists' Collaborative Group (EBCTCG)

July 11, 2024

Contents list

Page nos.

3 *Writing committee membership, Responsibilities, and EBCTCG steering committee*
4 *EBCTCG collaborators (1985-2023)*
11 *Table S1:* Characteristics of the trials and of the women included
20 *Supp Table S2:* Main references for the trials
35 *Figure S1*: Changes in trial population over time13
37 *Figure S2*: Improvements over time split by nodal status (1990-9 vs 2000-4 vs 2005-9)
39 *Figure S3*: Improvements over time split by TN status – women with ER-positive tumours
40 *Figure S4:* Improvements over time split by TN status – women with ER-negative tumours
41 *Figure S5*: Distant recurrence by nodal status, excluding women with HER2-positive tumours
42 *Figure S6*: Effect of adjustment on rate ratios over time, excluding women with HER2-positive tumours
43 *Figure S7*: Distant recurrence by nodal status, women with confirmed HER2-negative tumours only
44 *Figure S8*: Effect of adjustment on rate ratios over time, women with confirmed HER2-negative tumours only
45 *Figure S9*: Distant recurrence by nodal status, women with confirmed HER2-positive tumours only
46 *Figure S10*: Effect of adjustment on rate ratios over time, women with ER-positive tumours, 5 years of endocrine therapy scheduled

Writing committee

Robert Hills, Hongchao Pan, Jeremy Braybrooke, Richard Gray, Richard Peto, Rosie Bradley, Hui Liu, Carolyn Taylor, Paul McGale, David Dodwell (all from EBCTCG Secretariat, Clinical Trial Service Unit, Nuffield Department of Population Health, University of Oxford), Daniel F Hayes (University of Michigan Rogel Cancer Center), Mitch Dowsett (Royal Marsden Hospital and Institute of Cancer Research, London), Richard D Gelber (Department of Data Science, Dana-Farber Cancer Institute, Harvard Medical School, Boston), Jonas Bergh (Karolinska Comprehensive Cancer Center and Karolinska University Hospital, Stockholm), Sandra M. Swain (Georgetown Lombardi Comprehensive Cancer Center and MedStar Health, Washington, DC).

Responsibilities

The writing committee designed the study and drafted the report, circulated it for comment to the Early Breast Cancer Trialists' Collaborative Group (EBCTCG), then revised it. The data were analyzed by the EBCTCG Secretariat within the Nuffield Department of Population Health, Oxford, UK (HP, JB, RG, RP, RB, RH, HL, and DD), who vouch for the data and the analysis. Funders had no role in study design, data collection, data analysis, data interpretation or report writing. The EBCTCG secretariat had full access to the data and the writing committee had full access to analyses and final responsibility for deciding to submit for publication. There are no commercial sponsors or confidentiality agreements.

EBCTCG steering committee

J Bergh (co-chair), S Swain (co-chair), K Albain, S Anderson, R Arriagada, J Bartlett, E Bergsten-Nordström, J Bliss, R Bradley*, E Brain, J Braybrooke*, D Cameron, L Carey, M Clarke*, R Coleman, J Cuzick, N Davidson, L del Mastro, J Dignam, D Dodwell*, M Dowsett, B Ejlertsen, P Francis, JA Garcia-Saenz, R Gelber, M Gnant, M Goetz, P Goodwin, R Gray*, P Halpin-Murphy, D Hayes, C Hill, RK Hills*, R Jagsi, W Janni, S Loibl, E MacKinnon*, T Mamounas, S McIntosh, P McGale*, H Mukai, V Nekljudova, L Norton, H Pan*, R Peto*, M Piccart, P Poortmans, K Pritchard, V Raina, D Rea, M Regan, J Robertson, E Rutgers, R Salgado, D Slamon, T Spanic, J Sparano, G Steger, G Tang, C Taylor*, M Toi, A Tutt, G Viale, X Wang, T Whelan, N Wilcken, N Wolmark, K-D Yu. *EBCTCG Secretariat, NDPH.

EBCTCG collaborators, listed alphabetically by institution or group and then alphabetically by name

AARTM 048/13/2000 Multicentre Study Group, Spain—J A Alberro, B Ballester, P Deulofeu, R Fábregas, M Fraile, J M Gubern, J Janer, A Moral, J L de Pablo, G Peñalva, P Puig, M Ramos, R Rojo, P Santesteban, C Serra, M Solà, L Solarnau, J Solsona, E Veloso, S Vidal.

ACETBC, Tokyo, Japan—O Abe, R Abe, K Enomoto, K Kikuchi, H Koyama, H Masuda, Y Nomura, Y Ohashi, K Sakai, K Sugimachi, M Toi, T Tominaga, J Uchino, M Yoshida.

Addenbrooke's Hospital, Cambridge, UK—C E Coles, J L Haybittle.

AGO Breast Study Group (AGO-B), Germany—V Möbus.

American College of Surgeons Oncology Group (ACOSOG), U.S.A—A U Buzdar, K K Hunt, V J Suman.

Anglo-Celtic Cooperative Oncology Group, UK—J Crown, C F Leonard, J Mansi.

ARCOSEIN Group, France—G Calais, P Garaud.

Association Européenne de Recherche en Oncologie (AERO), France—C Delbaldo, P Piedbois, E Quinaux.

ATLAS Trial Collaborative Study Group, Oxford, UK—J Braybrooke, C Davies, R Gray, H C Pan, R Peto, J Sayer.

Auckland Breast Cancer Study Group, New Zealand—V J Harvey, I M Holdaway, R G Kay, B H Mason.

Australia & New Zealand Breast Cancer Trials Group, Newcastle, Australia—J F Forbes, P A Francis, N Wilcken.

Austrian Breast Cancer Study Group, Vienna, Austria—M Balic, R Bartsch, C Fesl, F Fitzal, H Fohler, M Gnant, R Greil, R Jakesz, C Marth, B Mlineritsch, G Pfeiler, C F Singer, L Sölkner, G G Steger, H Stöger.

Beatson Oncology Centre, Glasgow, UK—P Canney, H M A Yosef.

Belgian Adjuvant Breast Cancer Project, Liège, Belgium—C Focan.

Bergen Breast Cancer Group, Norway—H P Eikesdal, P E Lønning.

Berlin-Buch Akademie der Wissenschaften, Germany—U Peek.

Birmingham General Hospital, UK—G D Oates, J Powell.

Bordeaux Institut Bergonié, France—M Durand, L Mauriac.

Bordet Institute, Brussels, Belgium—J Crown, A Di Leo, S Dolci, V van Dooren, D Larsimont, J M Nogaret, C Philippson, M J Piccart.

Bradford Royal Infirmary, UK—M B Masood, D Parker, J J Price.

Breast Cancer Now Toby Robins Research Centre, Institute of Cancer Research, London, UK—A Tutt.

Breast Cancer Study Group of the Comprehensive Cancer Centre, Limburg, Netherlands—P S G J Hupperets.

Breast Cancer Trials Australia & New Zealand, Newcastle, Australia—B H Chua, J F Forbes, P A Francis, N Wilcken.

British Association of Surgical Oncology BASO II Trialists, London, UK—T Bates, R W Blamey, U Chetty, I O Ellis, E Mallon, D A L Morgan, J Patnick, S Pinder.

British Columbia Cancer Agency, Vancouver, Canada—C Lohrisch, A Nichol.

Canadian Cancer Trials Group, Kingston, Ontario, Canada—J M S Bartlett, V H Bramwell, B E Chen, S K L Chia, K Gelmon, P E Goss, M N Levine, W Parulekar, J L Pater, K I Pritchard, E Rakovitch, L E Shepherd, D Tu, T Whelan.

Cancer and Leukemia Group B, Washington DC, USA—D Berry, G Broadwater, C Cirrincione, H Muss, L Norton, L N Shulman, R B Weiss.

Cancer Care Ontario, Canada—H T Abu-Zahra.

Cancer Research Centre of the Russian Academy of Medical Sciences, Moscow, Russia—A Karpov, S M Portnoj.

Cancer Research UK Clinical Trials Unit (CRCTU), NCRI, Birmingham, UK—S Bowden, I Fernando, M Lee, C Poole, D Rea, D Spooner.

Cardiff Trialists Group, UK—P J Barrett-Lee, R E Mansel, I J Monypenny.

Case Western Reserve University, Cleveland, OH, USA—N H Gordon.

Central Oncology Group, Milwaukee, WI, USA—H L Davis.

Centre for Cancer Prevention, Wolfson Institute of Preventive Medicine, Queen Mary, University of London, UK—J Cuzick, I Sestak.

Centre Léon-Bérard, Lyon, France—Y Lehingue, P Romestaing.

Centre Paul Lamarque, Montpellier, France—J B Dubois.
Centre Regional François Baclesse, Caen, France—T Delozier, B Griffon, J Mace Lesec'h.
Centro Oncologico, Trieste, Italy—G Mustacchi.
Charles University in Prague, First Faculty of Medicine, Department of Oncology of the First Faculty of Medicine and General Teaching Hospital, Czech Republic—L Petruzelka, O Pribylova.
Cheltenham General Hospital, UK—J R Owen.
Chemo N0 Trial Group, Germany—N Harbeck, F Jänicke, C Meisner, M Schmitt, C Thomssen.
Chicago University, IL, USA—P Meier.
Chinese Academy of Medical Sciences, Beijing, People's Republic of China (in collaboration with the Oxford Nuffield Department of Population Health (NDPH))—Y Shan, Y F Shao, X Wang, B Xu, D B Zhao (NDPH: Z M Chen, H C Pan).
Christie Hospital and Holt Radium Institute, Manchester, UK—A Howell, R Swindell.
Clinical Trials Research Unit (CTRU), University of Leeds, UK—G Booth, J Brown, J Emmerson.
Coimbra Instituto de Oncologia, Portugal—J Albano, C F de Oliveira, H Gervásio, J Gordilho.
Cookridge Hospital, Leeds, UK—D Dodwell.
Copenhagen Breast Cancer Trials, Copenhagen, Denmark—B Ejlertsen, M-B Jensen, H Mouridsen.
Dana-Farber Cancer Institute, Boston, MA, USA—R S Gelman, J R Harris, C Henderson, C L Shapiro, E Winer.
Danish Breast Cancer Cooperative Group, Copenhagen, Denmark— P Christiansen, B Ejlertsen, M Ewertz, M-B Jensen, A Knoop, H T Mouridsen (deceased), B V Offersen, T F Tvedskov.
Düsseldorf University, Germany—T Fehm, H J Trampisch.
Dutch Breast Cancer Trialists' Group (BOOG)— S Linn, H M Oosterkamp, P Peer, E J Rutgers, V Tjan-Heijnen, E van Leeuwen-Stok, A G J van Rossum, E van Werkhoven, S Vliek.
Dutch Working Party for Autologous Bone Marrow Transplant in Solid Tumours, Amsterdam & Groningen, Netherlands—O Dalesio, E G E de Vries, S Rodenhuis, H van Tinteren .
Eastern Cooperative Oncology Group, Boston, MA, USA—R L Comis, N E Davidson, R Gray, N Robert, G Sledge, L J Solin, J A Sparano, D C Tormey, W Wood.
Edinburgh Breast Unit, UK—D Cameron, U Chetty, J M Dixon, P Forrest, W Jack, I Kunkler.
Elim Hospital, Hamburg, Germany—J Rossbach.
Erasmus MC/Daniel den Hoed Cancer Center, Rotterdam, Netherlands—J G M Klijn, A D Treurniet-Donker, W L J van Putten.
European Cooperative Trial in Operable Breast Cancer (ECTO)—W Eiermann, L Gianni, P Valagussa.
European Institute of Oncology, Milan, Italy—V Galimberti, N Rotmensz, U Veronesi, G Viale.
European Organization for Research and Treatment of Cancer, Brussels, Belgium—H Bartelink, N Bijker, J Bogaerts, H Bonnefoi, F Cardoso, T Cufer, J P Julien, M Piccart, C Poncet, P M Poortmans, E Rutgers, C J H van de Velde.
Evanston Hospital, IL, USA—M P Cunningham.
Federation Nationale des Centres de Lutte Contre le Cancer (FNCLCC) Breast Group, France—T Delozier, M Spielman, J Mace Lesec'h.
Finnish Breast Cancer Group, Finland—R Huovinen, H Joensuu.
Fondazione Maugeri Pavia, Italy—A Costa.
Fondazione Michelangelo, Milan, Italy—G Bonadonna, L Gianni, P Valagussa.
Fox Chase Cancer Center, Philadelphia, PA, USA—L J Goldstein.
French Adjuvant Study Group (GFEA), Guyancourt, France—J Bonneterre, P Fargeot, P Fumoleau, P Kerbrat, E Luporsi, M Namer.
Fudan University Shanghai Cancer Center, Shanghai, China—Z M Shao, K D Yu.
GEICAM, Spanish Breast Cancer Group, Spain—E Carrasco, M Martin, M A Segui.
German Adjuvant Breast Group (GABG), Frankfurt, Germany—W Eiermann, J Hilfrich, W Jonat, M Kaufmann, R Kreienberg, M Schumacher.

German Breast Cancer Study Group (BMFT), Freiburg, Germany—G Bastert, H Rauschecker, R Sauer, W Sauerbrei, A Schauer, M Schumacher.
German Breast Group (GBG), Neu-Isenburg, Germany—J U Blohmer, S D Costa, H Eidtmann, B Gerber, C Jackisch, S Kümmel, S Loibl, V Nekljudova, G von Minckwitz.
Ghent University Hospital, Belgium—A de Schryver, L Vakaet.
GIVIO Interdisciplinary Group for Cancer Care Evaluation, Chieti, Italy—M Belfiglio, A Nicolucci, F Pellegrini, M C Pirozzoli, M Sacco, M Valentini.
Glasgow Victoria Infirmary, UK—C S McArdle, D C Smith, S Stallard.
Groote Schuur Hospital, Cape Town, South Africa—D M Dent, C A Gudgeon, A Hacking, E Murray, E Panieri, ID Werner.
Gruppo Interdisciplinare Veneto di Oncologia Mammaria (GIVOM), Italy—G L De Salvo, P Del Bianco, G Zavagno.
Grupo Oncológico Cooperativo del Sur (GOCS), Argentina—B Leone, C T Vallejo, A Zwenger.
Gruppo Oncologico Clinico Cooperativo del Nord Est, Aviano, Italy—E Galligioni.
Gruppo Oncologico Dell'Italia Meridionale (GOIM), Rome, Italy—M Lopez.
Guadalajara Hospital de 20 Noviembre, Mexico—A Erazo, J Y Medina.
Gunma University, Japan—J Horiguchi, H Takei.
Guy's Hospital, London, UK—I S Fentiman, J L Hayward, R D Rubens, D Skilton.
Heidelberg University I, Germany—H Scheurlen.
Heidelberg University II, Germany—M Kaufmann, H C Sohn.
Helios Klinikum Berlin-Buch, Germany—M Untch.
Hellenic Breast Surgeons Society, Greece—U Dafni, C Markopoulos.
Hellenic Cooperative Oncology Group, Athens, Greece—G Fountzilas, G-A Koliou.
Hellenic Oncology Research Group, Greece—D Mavroudis.
Helsinki Deaconess Medical Centre, Finland—P Klefstrom.
Helsinki University, Finland—C Blomqvist, T Saarto.
Herceptin-Adjuvant (HERA)/Breast International Group (BIG)—D Cameron, E de Azambuja, R Gelber, M Piccart, M Regan.
Hospital del Mar, Barcelona, Spain—M Gallen.
Humanitas Cancer Center, Milan, Italy—G Canavese, C Tinterri.
Innsbruck University, Austria—R Margreiter.
Institut Claudius Regaud, Toulouse, France—B de Lafontan, J Mihura, H Roché.
Institut Curie, Paris, France—B Asselain, R J Salmon, J R Vilcoq.
Institut Curie - Hôpital René Huguenin, Paris, St Cloud, France—E Brain, B de La Lande, E Mouret-Fourme.
Institut Gustave-Roussy, Paris, France—F André, R Arriagada, S Delaloge, C Hill, S Koscielny, S Michiels, P Pélissier, C Rubino.
Institute of Cancer Research Clinical Trials and Statistics Unit (ICR-CTSU, NCRI), UK—R A'Hern, J Bliss, P Ellis, L Kilburn, J R Yarnold.
Instituto de Investigación Sanitaria Gregorio Marañón, Universidad Complutense, GEICAM, CIBERONC, Madrid, Spain—M Martin.
Instituto Nacional de Cancer, Brazil—J Bines, R M B Sarmento.
Integraal Kankercentrum, Amsterdam, Netherlands—J Benraadt, M Kooi, A O van de Velde, J A van Dongen, J B Vermorken.
International Atomic Energy Agency (IAEA), Vienna, Austria—G W Jones, E Rosenblatt, E Zubizarreta.
International Breast Cancer Study Group (IBCSG), Bern, Switzerland—M Castiglione, A Coates, M Colleoni, J Collins, J Forbes, R D Gelber, A Goldhirsch, J Lindtner, O Pagani, K N Price, M M Regan, C M Rudenstam, H J Senn, B Thuerlimann.

International Collaborative Cancer Group, London, UK—J M Bliss, C E D Chilvers, R C Coombes, M Espie, E Hall, L Kilburn, M Marty.
International Drug Development Institute, Louvain-la-Neuve, Belgium—M Buyse.
International TABLE Study Group, Berlin, Germany—K Possinger, P Schmid, M Untch, D Wallwiener.
ISD Cancer Clinical Trials Team (incorporating the former Scottish Cancer Therapy Network), Edinburgh, UK—L Foster, W D George, H J Stewart, P Stroner.
Israel NSABC, Tel Aviv, Israel—R Borovik, H Hayat, M J Inbar, T Peretz, E Robinson.
Istituto Nazionale per lo Studio e la Cura dei Tumori, Milan, Italy—G Bonadonna, C Brambilla, T Camerini, F Formelli, G Martelli, M G Di Mauro, A Rossi, P Valagussa.
Istituto Nazionale Tumori IRCCS Fondazione Pascale, Napoli, Italy—C Gallo, F Perrone.
Istituto Scientifico Romagnolo per lo Studio e la Cura dei Tumori, Meldola, Italy—D Amadori, F Boccardo, O Nanni, A Rubagotti, E Scarpi.
Italian Cooperative Chemo-Radio-Surgical Group, Bologna, Italy—A Martoni, F Pannuti.
Italian Oncology Group for Clinical Research (GOIRC), Parma, Italy— R Camisa, A Musolino, R Passalacqua.
Japan Breast Cancer Research Group (JBCRG), Japan—H Masuda, M Toi, T Ueno.
Japan Clinical Oncology Group–Breast Cancer Study Group, Japan—H Iwata, T Shien.
Japanese Association for Treatment of Breast Cancer with Zoladex, Japan—S Mitsuyama, Y Nomura, S Ohno.
Japanese Foundation for Multidisciplinary Treatment of Cancer, Tokyo, Japan—O Abe, T Ikeda, K Inokuchi, K Kikuchi, K Sawa.
Kanagawa Breast Oncology Group, Japan—T Ishikawa, K Narui.
Kawasaki Medical School, Japan—H Sonoo.
Klinikum Bayreuth, Germany—M Sadoon, A H Tulusan.
Kobe Breast Cancer Oncology Group, Japan—N Kohno, K Matsumoto, M Miyashita, S Takao.
japanese
Korean Cancer Study Group (KCSG), Seoul, Republic of Korea—J-H Ahn, K H Jung.
Korean Radiation Oncology Group, Seoul (KROG), Republic of Korea—C-O Suh.
Krakow Institute of Oncology, Poland—S Korzeniowski, J Skolyszewski.
Kumamoto University Group, Japan—M Ogawa, J Yamashita.
Leiden University Medical Center, Netherlands—E Bastiaannet, G J Liefers, C J H van de Velde.
Leuven Akademisch Ziekenhuis, Gasthuisberg, Belgium—R Christiaens, P Neven, R Paridaens, W Van den Bogaert.
Ludwig-Maximilians University, Munich, Germany—S Braun, H Sommer.
Marseille Laboratoire de Cancérologie Biologique APM, France—P Martin, S Romain.
Medical University Vienna – General Hospital - Department of Obstetrics and Gynaecology and Department of Medicine I, Vienna, Austria—M Janauer, M Seifert, P Sevelda, C C Zielinski.
Memorial Sloan-Kettering Cancer Center, New York, NY, USA—T Hakes, C A Hudis, L Norton, R Wittes.
Metaxas Memorial Cancer Hospital, Athens, Greece—P Foroglou, G Giokas, D Kondylis, B Lissaios (deceased).
Mexican National Medical Center, Mexico City, Mexico—R de la Huerta, M G Sainz.
Multicentre Group, Germany—S Kümmel, M Reinisch.
National Cancer Center, Goyang, South Korea—K S Lee, B-H Nam, J Ro.
National Cancer Institute, Bethesda, MD, USA—K Camphausen, D Danforth, A Lichter, M Lippman, D Smart, S Steinberg.
National Cancer Institute, Naples, Italy—A de Matteis, F Perrone.
National Cancer Institute of Bari, Italy—C D'Amico, M Lioce, A Paradiso.
National Kyushu Cancer Center, Japan—Y Nomura, S Ohno.
National Surgical Adjuvant Breast and Bowel Project (NSABP), Pittsburgh, PA, USA—S Anderson, H Bandos, A Brown (deceased), J Bryant (deceased), R Cecchini, J Costantino, J Dignam, B Fisher, C Geyer, E P Mamounas, S Paik, C Redmond, E Romond, S Swain, G Tang, M A Torres, L Wickerham, N Wolmark, G Yothers.

National Surgical Adjuvant Study Group (N-SAS-BC), Japan—T Aihara, Y Hozumi, Y Nomura.
Neo-tAnGo Trial Group, UK—H Earl, L Hiller, A-L Vallier.
Nolvadex Adjuvant Trial Organisation, London, UK—M Baum, I M Jackson (deceased), M K Palmer.
North Central Cancer Treatment Group (NCCTG), Mayo Clinic, Rochester, MN, USA—A Moreno-Aspitia, J N Ingle, E Perez, V J Suman.
North Sweden Breast Cancer Group, Umeå, Sweden—A Andresson, N O Bengtsson, H Jonsson, M Sund.
North-West Oncology Group (GONO), Italy—L Del Mastro, M Venturini.
North-Western British Surgeons, Manchester, UK—J P Lythgoe, R Swindell.
Northwick Park Hospital, London, UK—M Kissin.
Norwegian Breast Cancer Group, Oslo, Norway—B Erikstein, E Hannisdal, A B Jacobsen, K V Reinertsen, J E Varhaug.
Norwegian Radium Hospital, Oslo, Norway—B Erikstein, S Gundersen, M Hauer-Jensen, H Høst, A B Jacobsen, R Nissen-Meyer.
Nottingham City Hospital, UK—R W Blamey, A K Mitchell, D A L Morgan, J F R Robertson.
Nuffield Department of Population Health (NDPH), Oxford, UK (ie, members of the Oxford-based EBCTCG Secretariat)—G Beake, R Berry, C Boddington, R Bradley, J Braybrooke, J A Burrett, M Clarke, D Cutter, C Davies, L Davies, D Dodwell, F Duane, V Evans, L Gettins, J Godwin, R Gray, R Hills, S James, A Kerr, H Liu, Z Liu, E MacKinnon, G Mannu, P McGale, T McHugh, P Morris, M Nakahara, H C Pan, R Peto, S Read, E Straiton, C Taylor, H Taylor, Y Wang, Z Wang.
Oita Prefectural Hospital, Japan—H Ueo.
Oncofrance, Paris, France—M Di Palma, G Mathé (deceased), J L Misset.
Ontario Clinical Oncology Group, Hamilton, Canada—M Levine, K I Pritchard, T Whelan.
Osaka City University, Japan—K Morimoto.
Osaka National Hospital, Japan—K Sawa, Y Takatsuka.
Ospedale Policlinico San Martino, Genova, Italy—D Bedognetti**,** C Bighin, P Bruzzi, L Del Mastro, B Dozin, S Pastorino, P Pronzato, M R Sertoli.
Oxford University Hospitals NHS Foundation Trust, Churchill Hospital, Oxford, UK—E Crossley, A Harris, D Talbot, M Taylor.
PACS Study Group, France—T Bachelot, S Delaloge, T Delozier, J Lemonnier, A-L Martin, S Michiels, H Roché, M Spielmann.
Parma Hospital, Italy—G Cocconi, B di Blasio.
Petrov Research Institute of Oncology, St Petersburg, Russia—V Ivanov, R Paltuev, V Semiglazov.
Piedmont Oncology Association, Winston-Salem, NC, USA—J Brockschmidt, M R Cooper.
Pretoria University, South Africa—C I Falkson.
ProBONE study group, Marburg, Germany—P Hadji.
Royal Marsden NHS Trust, London and Sutton, UK—R A'Hern, M Dowsett, A Makris, M Parton, K Pennert, T J Powles, I E Smith, J R Yarnold.
SABRE trial group (international)—G Clack, C Van Poznak.
St George Hospital, Sydney, Australia—L Browne, P Graham.
St George's Hospital, London, UK—J C Gazet.
St Luke's Hospital, Dublin, Ireland—N Corcoran.
Sardinia Oncology Hospital A Businico, Cagliari, Sardinia—N Deshpande, L di Martino.
Samuel Oschin Comprehensive Cancer Center, Los Angeles, USA—A E Giuliano.
SASIB International Trialists, Cape Town, South Africa—P Douglas, A Hacking, H Høst, A Lindtner, G Notter.
Saskatchewan Cancer Foundation, Regina, Canada—A J S Bryant, G H Ewing, L A Firth, J L Krushen-Kosloski.
Scandinavian Adjuvant Chemotherapy Study Group, Oslo, Norway—R Nissen-Meyer.
Shanghai Jiao Tong University School of Medicine, China—X Chen, K Shen.
South Sweden Breast Cancer Group, Lund, Sweden—H Anderson, F Killander, P Malmström, L Rydén.

South-East Sweden Breast Cancer Group, Linköping, Sweden—L-G Arnesson, J Carstensen, M Dufmats, H Fohlin, B Nordenskjöld, M Söderberg, M Sundqvist.
South-Eastern Cancer Study Group and Alabama Breast Cancer Project, Birmingham, AL, USA—J T Carpenter.
Southampton Oncology Centre, UK—R I Cutress, G T Royle, P D Simmonds.
Southwest Oncology Group, San Antonio, TX, USA—K Albain, W Barlow, G T Budd, J Gralow, D Hayes, G Hortobagyi, R Jagsi, S Martino, L Pusztai, P Sharma, A Thompson.
Stockholm Breast Cancer Study Group, Sweden—J Bergh, T Bondesson, F Celebioglu, K Dahlberg, T Fornander, I Fredriksson, J Frisell, E Göransson, M Iiristo, U Johansson, E Lenner, L Löfgren, P Nikolaidis, L Perbeck, S Rotstein, K Sandelin, L Skoog, G Svane, E af Trampe, C Wadström.
SUCCESS Study Group, Germany—T Friedl, W Janni, H Sommer.
SweBCG 91 RT trial group, Sweden—P-O Bendahl, E Holmberg, P Karlsson, F Killander, E Nimeus. Swiss Group for Clinical Cancer Research (SAKK), Bern, and OSAKO, St Gallen, Switzerland—M Castiglione, A Goldhirsch, R Maibach, H J Senn, B Thürlimann.
Tamoxifen Exemestrane Adjuvant Multinational (TEAM) trial—J M S Bartlett, E Bastiaannet, P Hadji, Y Hozumi, D Rea, C J H van de Velde.
TACT Trial Group, London, UK—P Barrett-Lee, J M Bliss, P Ellis, L Kilburn.
Tampere University Hospital, Finland—K Holli, K Rouhento.
Tel Aviv Sourasky Medical Center, Sackler School of Medicine, Tel Aviv University, Israel—T Safra.
Tel Aviv University, Israel—H Brenner, A Hercbergs.
Texas Oncology-Baylor Charles A. Sammons Cancer Center, US Oncology Network, Dallas, TX, USA—J L Blum.
Tokyo Cancer Institute Hospital, Japan—M Yoshimoto.
Toronto-Edmonton Breast Cancer Study Group, Canada—A H G Paterson, K I Pritchard.
Toronto Princess Margaret Hospital, Canada—A Fyles, J W Meakin, T Panzarella, K I Pritchard.
TRIO (formerly Breast Cancer International Research Group, BCIRG)—V Bee, J Crown, H Fung, J Mackey, M Martin, M Press, D Slamon.
Tunis Institut Salah Azaiz, Tunisia—J Bahi.
UCBG, French Breast Cancer Intergroup UNICANCER, France—S Delaloge, W Jacot, J Lemonnier, A L Martin, M Spielmann.
UK Multicentre Cancer Chemotherapy Study Group, London, UK—M Reid, M Spittle.
UK/ANZ DCIS Trial—H Bishop, N J Bundred, J Cuzick, I O Ellis, I S Fentiman, J F Forbes, S Forsyth, W D George, S E Pinder, I Sestak.
UK/Asia Collaborative Breast Cancer Group, London, UK—G P Deutsch, R Gray, D L W Kwong, V R Pai, R Peto, F Senanayake.
Universitätsklinikum Ulm, Germany—T Friedl, W Janni.
University and Istituto Nazionale per la Ricerca sul Cancro, Genoa, Italy on behalf of GROCTA trialists—F Boccardo, A Rubagotti.
University College London, UK—M Baum, S Forsyth, A Hackshaw, J Houghton, J Ledermann, K Monson, JS Tobias.
University Federico II, Naples, Italy—C Carlomagno, M De Laurentiis, S De Placido.
University Hospitals, Leuven, Leuven, Belgium—H Wildiers.
University of Hull and Lincoln County Hospital, UK—O Eremin, L G Walker.
University Medical Center Schleswig-Holstein, Campus Kiel, Germany—C Schem.
University of Edinburgh, UK—L Williams.
University of Leeds, UK—R Bell, D Cameron, R E Coleman, D Dodwell, S Hinsley, H C Marshall.
University of Michigan, USA—D Hayes, L J Pierce.
University of Padua, Padova, Italy—S M M Basso, F Lumachi.
University of Saarland, Germany—E Solomayer.
University of Sheffield, UK—R E Coleman, J M Horsman, J Lester, M C Winter.

University of Texas MD Anderson Cancer Center, Houston, TX, USA—A U Buzdar, L Hsu.
University of West Indies, Jamaica and Bahamas—G W Jones.
University of Wisconsin, USA—R R Love.
Uppsala-Örebro Breast Cancer Study Group, Sweden—J Ahlgren, H Garmo, L Holmberg, G Liljegren, H Lindman, F Wärnberg.
U.S. Oncology, Houston, USA—L Asmar, J L Blum, S E Jones, J O'Shaughnessy, N Robert.
Warwick Clinical Trials Unit, University of Warwick, UK—J Dunn, H Earl, L Hiller.
Washington University, St Louis, Missouri, USA—R Aft.
West German Study Group (WSG), Germany—O Gluz, N Harbeck, C Liedtke, U Nitz.
West of Scotland Breast Trial Group, Glasgow, UK—A Litton.
West Sweden Breast Cancer Study Group, Gothenburg, Sweden—J Heiman, E Holmberg, P Karlsson, B K Linderholm, F Wärnberg.
Western Cancer Study Group, Torrance, CA, USA—R T Chlebowski.
Würzburg University, Germany—H Caffier.
Z-FAST, ZO-FAST & E-ZO-FAST study groups (international)—A M Brufsky, R E Coleman, H A Llombart, on behalf of Novartis Pharmaceuticals.
ZIPP trial (Cancer Research UK; GIVIO, Italy; South East Sweden, and Stockholm)—M Baum, T Fornander, A Hackshaw, A Nicolucci, B Nordenskjold, R Sainsbury.

***Table S1:* Characteristics of trials and women in analyses** ET: endocrine therapy, AI: aromatase inhibitor, Tam: tamoxifen

Trial code	Trial name	Comparison	Numbers with ER-positive disease in analyses by period			Numbers with ER-negative disease in analyses by period		
			1990-9	2000-4	2005-9	1990-9	2000-4	2005-9
1979T	Umeå part of SESBCG-Örebro-Karlstad Study	Tam 5 years vs. 1 - 2 years	168	-	-	67	-	-
1982R	IKA C8209 Amsterdam	Tam 1 year vs. Control	-	-	-	49	-	-
1984K	GUN-3 Naples	3(CMFq4; [E75×2V1·4×2]q3) vs. 6(C100×14M40×2F600×2)q4	-	-	-	21	-	-
1984Q	ABCSG Trial 1	1V1A20; 1C300x3M25x2F600x2 vs. Control	-	-	-	104	-	-
1984R	DFCI Lumpectomy RT Seq Trial	RT;CMFAP vs. CMFAP;RT	25	-	-	22	-	-
1985A	CALGB Study CLB-8541	4F600x2A60C600 vs. 6F400x2A40C400)q4	98	-	-	85	-	-
1985M	Bordeaux Patey Mastectomy Neo vs Adj Chemo	3E50V1000M20; 3Mit10Tt20Vd4000 pre vs. post	-	-	-	21	-	-
1986A	E.O.R.T.C. Trial 10854	1F500A50C600 36hr post-surgery vs. Control	-	-	-	10	-	-
1986H	IBCSG Trial VI	3CMF; CMF (m6,9,12 vs. m9,12,15)	365	-	-	78	-	-
1986L	M.D. Anderson Study 86-12	4CAF;4MVb vs 6CAF	20	-	-	101	-	-
1986P	French Adjuvant Study Group GFEA 01	3(FE75C) vs. 3(F500E50C500)	-	-	-	180	-	-
1987A	C.R.C. Under 50s Trial (part of `ZIPP')	Tam ± Gos 2y+	-	-	-	255	-	-

1987C	N.S.A.B.C. Israel Mastectomy or Lumpectomy	(CMF vs. C+Mit+F)q3	-	-	-	14	-	-
1988A	NSABP Protocol B-18	4A60C600 pre vs. post	135	-	-	111	-	-
1988B	NSABP Protocol B-19	MFC q1 x 6 vs. (MF + Folinic Acid) q1 x6	-	-	-	243	-	-
1988D	IBCSG Trial IX	3(C100×14M40×2F600×2)q4	1130	-	-	260	-	-
1988R	Jules Bordet Chemo High vs. Std	8(E100C830) vs. 8(E60C500)	274	-	-	83	-	-
1989&	Marsden Bisphosphonate Trial	Clodronate vs. Control	156	-	-	85	-	-
1989@	Bari Chemotherapy Trial	6(F500×2E50C500) vs. Control	-	-	-	64	-	-
1989B	SWOG 8814 / INT 0100	6 FAC;Tam 5yr vs. 6FAC+Tam 5yr vs. Tam 5yr	1713	-	-	1054	-	-
1989C	Gustave-Roussy FNCLCC Trial	6F500A/E50C500q3-4/? ± Ov Irr or Oopho	-	-	-	88	-	-
1989D	Gustave-Roussy Chemotherapy Trial	6(F500[A50/E50]C500)q3-4 vs. Control	-	-	-	112	-	-
1989E	G.R.O.C.T.A. IV	Tam 5 yr vs. 1 - 2 yr	68	-	-	48	-	-
1989F	ECOG EST 5188 / INT 0101	6C100x14M30x2F500x2; ± (Gos 5yr ± Tam 5yr)	716	-	-	-	-	-
1989G	ECOG EST 3189 = INT 0108	16C100x7A40F600/300x2V1M100qw vs. 6C100x14A30x2F1000q4	-	-	-	438	-	-
1989M	NSABP Protocol B-22	2A60C1200q3;2A60q3 vs. 4A60C600q3 vs. 4A60C1200	549	-	-	429	-	-
1989N	E.O.R.T.C. Trial 10882/22881	e-/Tang/Imp 16 Gy- 50Gy ± 10-15Gy boost	-	-	-	709	-	-
1989P	DBCG 89c	Tam 2yr vs. Tam 1yr vs. Tam 6m + Megestrol Acetate 6m	144	-	-	-	-	-
1989W	Umeå part of DBCG89d-Umeå-	(9FE60C vs. 9(C600M40F600)) ± Pamidronate	-	-	-	263	-	-

	Uppsala-Örebro Trial							
1990*	GABG Germany Clodronate Trial	Clodronate 2yr vs. Control	-	-	-	33	-	-
1990@	Helsinki Toremifene Trial	(Tor vs. Tam) x 3yr	-	-	-	57	-	-
1990C	French Adjuvant Study Group GFEA 05	6(FE100C) vs. 6(F500E50C500)	-	-	-	272	-	-
1990F	Zoladex Versus CMF (ZEBRA)	Gos 2y vs. 6 CMF	-	-	-	143	-	-
1990K	Sto 6 Stockholm	Tam ± Megace ((160mg/d) x 3m) q6m x 4)	-	-	-	67	-	-
1990L	Sto 7 Stockholm	Tam 2 year vs. Control	-	-	-	277	-	-
1990S	IBCSG Trial VIII	(6(C100×14M40×2F600×2)q4 ± Gos 18m vs. 2yr) vs. Control	-	-	-	197	-	-
1990W	ABCSG Study V	Gos 3y Tam 5y vs. 6 C600x2M40x2F600x2	1995	-	-	-	-	-
1990Z	GOIRC SANG 2	4(E30×4)q4 vs. 6(C600×2M40×2F600×2)q4	-	-	-	39	-	-
1991C	E.O.R.T.C. Trial 10901	6CMF or 4-6CAF or CEF or FAC or FEC ± Tam x 3 yr	-	-	-	103	-	-
1991H	NSABP Protocol B-23	(4A60C600 vs. 6(C100×14M40×2F600×2)q4) ± Tam 10yr	27	-	-	1418	-	-
1991P	SweBCG 91-RT	MV RT 48-54Gy Br/wall vs. Control	-	-	-	125	-	-
1991Q	G.O.C.S.I. MAM1	(4A75; 6CMFq4 vs. 6(C100×14M40×2F600×2)q4) ± (Gos 3yr Tam 2yr)	-	-	-	50	-	-
1992D	IKA C9203 Amsterdam	Tam 3yr ± 4(E90C600)	-	-	-	13	-	-
1992E	GONO-MIG 1 Genova	6F600E60C600 (q2 vs. q3)	561	-	-	324	-	-
1992F	NSABP Protocol B-25	4A60C2400q3 vs. 4A60C1200q3 vs. 2A60C2400;2A60q3	485	-	-	549	-	-
1992N	C/9/91 / HMFEC London	(8(F600E75C600) vs. 8(F600E50C600)) ± Tam 5yr	123	7	-	110	3	-
1992Q	FBCG92-01 Finland	(Tor vs. Tam) x 3yr	-	-	-	185	-	-

1993B	MA.12 Canada	CMF/FEC/AC ± Tam 5yr	13	-	-	25	-	-
1993C	GABG-4-A-93 Germany	Gos 2yr vs. 3C500x2M40x2F600x2	40	3	-	715	86	-
1993H	IBCSG Trial 11-93	Ovarian Ablation then Tam 5yr ± 4([A60/E90]C600)	160	-	-	-	-	-
1993J	Fondazione Michelangelo High-Dose Therapy Trial	(1C7000];1M80002E120;Tt600Me160-180(BMT/SCT) vs.3E120;6C600M40F600) + Tam 5yr	91	-	-	33	-	-
1993L	IBCSG Trial 10-93	(± Ax Dis);Tam 5 yr	140	25	-	12	7	-
1993M	IBCSG Trial 12a-93	(Tam vs. Tor) ±4AC (concurrent vs. sequential)	384	-	-	-	-	-
1993N	IBCSG 13-93	(AC;16 wk rest;CMF vs. AC;CMF) ± Tax 5yr	307	-	-	275	-	-
1993P	IBCSG 14a-93	4ACq3;3CMFq4;Tam 5yr vs. 4ACq3;16wk-off;3CMFq4;Tam 5yr	464	-	-	229	-	-
1993Q	IBCSG 15-95	(PSBCS+3E200C4000q3 vs. 4EC/ACq3;3CMFq4);Tam 5yr	10	2	-	53	2	-
1993S	Chemo-N0-Trial Germany	6(C500×2M40×2F600×2)q4 vs. Control	-	-	-	66	-	-
1993V	M93SCB high dose	4FEC;(C6000Tt480carbP1600;ASCT vs. 1FEC);RT +Tam	339	-	-	128	-	-
1994B	MD Anderson Protocol 94-00	4P250 pre; 4FAC post vs. 4FAC pre; 4FAC post	15	-	-	21	-	-
1994C	C.A.L.G.B. Study 9343	Lumpec + Br RT + Tam 5y vs Lumpec + Tam 5y	245	-	-	-	-	-
1994D	C.A.L.G.B. Study 9344 / Intergroup 0148	4(A90 + filgrastim vs. A75 vs. 60)C600; ± 4P175	1435	-	-	812	-	-
1994H	Huguenin FNCLCC Radiotherapy Trial	4CMzFq3+RT vs. 4FECq3;RT	271	-	-	103	-	-
1994J	GOIRC SANG 2B R1	6(C600 c1,2,4,5 M40 c1,3,4,6 F600 c2,3,5,6 (rotational weave) E40V1·4E40×2)q4 e) vs. 6(C600×2M40×2F600×2)q4	312	22	-	99	1	-
1994R	PEGASE 01	4FECq3±(1CMzMel+PBSCT)	-	-	-	67	-	-

1994S	ECOG EST3193/INT0142	Tam ± Gos/Lpr/Ooph/Ov Irr	330	-	-	-	-	-
1995K	NSABP Protocol B-28	4AC; 4P225 vs. 4A60C600<br>P225×4 A240 vs. A240 0	982	-	-	790	-	-
1995M	GEICAM 9401	4 EC; Tam 5yr	156	28	-	19	-	-
1995R	ECOG E-EB193 / INT-0151	Tam ± fenretinide	308	-	-	-	-	-
1995S	Protocol CNR-9502 Milan	(RT to B ± AD) + Tam 5yr	136	8	-	19	1	-
1995T	HORG Docetaxel Trial	4D100; 4EC vs. 6(F700E75C700)	76	171	-	25	49	-
1996A	NEAT Trial UCRI	4E100; 4CMFq4 vs. 6(C100×14M40×2F600×2)q4	-	-	-	334	152	-
1996E	ABCSG Study IX	Tam 5yr ± 4(F600E60C600)	150	46	-	-	-	-
1996J	ATAC Trial CRUK	(Ana vs. Tam vs. Tam+Ana) 5yr	3710	30	-	371	1	-
1996R	HCFU Trial Japan	CMF+Tam vs. FC+Tam	-	-	-	38	-	-
1996U	ARCOSEIN Study	3CMzFq3+RT; 3CMzFq3 vs. 6CMzFq3;RT	140	16	-	110	7	-
1996W	ECTO Milan	4E60P200; 4C600x2M40x2F600x2 (pre vs. post) vs. 4(C600×2M40×2F600×2)q4 (post)	179	230	-	80	124	-
1996X	GONO-MIG 5	4E90P175 vs. 6(F600E60C600)	737	240	-	-	-	-
1996Y	ABCSG Trial VIII	Tam 2yr; (Ana vs Tam) 3yr then RT vs. Control	1098	2105	-	-	-	-
1997A	C/14/96 DEVA ICCG	3Eq4; 3D100 vs. 6(E50×2)q4	89	341	34	26	58	6
1997D	C.A.L.G.B. Study 9741 / Intergroup C9741	((4A60;4P175;4C600) or (4AC\;4P175))[q2 vs. q3]<br>((4A60;4P175;4C600) vs. (4A60C600;4P175))[q2 (+ filgrastim) vs. q3]	1018	-	-	480	-	-
1997J	PACS 01 France	3FEC; 3D100 vs 6(F500E100C500)<br>D100×3 E300 vs E600 E300	861	117	-	329	28	-
1997L	BCIRG 001 / GEICAM 9703 (RP56976-V-316)	6D75AC vs 6(F500A50C500)	941	-	-	297	-	-
1997N	USON Trial 97-35	4(D75C600 vs. AC) q3	640	1	-	280	-	-
1997R	HeCOG HE 1097	3Eq2; 3P250q2; 3CMFq2 vs. 4(E110)q2; 4(C840M50F840)q2	115	48	-	29	15	-

1997U	IBIS 03 Italy	4E100q3;4C600M40F600 vs. 4C600M40F600;4E100q3 vs. 6C600M40F600q4	243	543	-	43	178	-
1997V	IBIS 02 Italy	4P175;4E75Vb25x2 vs. 4E100;4C600M40x2F600x2	41	75	-	14	11	-
1998@	NAFTA USA	(Tor vs Tam) x 5yr	277	664	-	-	-	-
1998D	BIG 02-98 / TAX 315 / GEICAM 9803	(4(A50D75) vs. 4(A75D100) vs. 4(A75C100) vs. 4(A60C600)); 3(C100×14M40×2F600×2)q4	407	1282	-	165	464	-
1998K	MD Anderson Protocol 98-240	(12P150/175 q1 vs. 4P225 q3); 4C500A50F500q3 pre	40	88	-	20	51	-
1998M	IBCSG 18-98 / BIG 01-98 / FEMTA	Let 5yr vs (Let 2yr; Tam 3yr) vs. Tam 2yr; (Let vs Tam) 3yr	1652	5228	-	33	36	-
1998T	ECOG EST2197	4AD60 vs. 4A60C600	1655	168	-	791	89	-
1999%	GEICAM 9805 / TARGET 0	6D75AC vs. 6(F500A50C500)	48	568	-	18	331	-
1999G	NSABP Protocol B-30	(4A50-60C600D60-75 vs. 4A50-60C600;4D100 vs. 4A50-60D60-75)q3	-	2421	-	-	998	-
1999K	GEICAM 9906	4FEC; 8(P100)q1 vs. 6(F600E90C600)	5	869	-	1	148	-
1999N	RAPP-01 France	4A50D75q3 vs. 4A60C600q3	46	387	-	10	107	-
1999V	ECOG E1199/Intergroup	4ACq3; (12P80q1 vs. 4P175q3 vs. 4D100q3 vs. 12D35q1)	22	3054	-	9	1188	-
1999W	PRIME Scotland	Br [MV RT 40-50Gy 20-25f + BST	-	72	-	-	-	-
2000=	USON Trial 99-016	4A50P200 q3;12P175 q1 vs. 4AC;4P175 q3	-	968	-	-	558	-
2000A	Fondazione Michelangelo GMB/99/02	4(A60P200)q3;(6P100x2 vs. 4C600M40x2F600x2) q3 vs.4(E75V25x2)q3;(6P100x2q3 vs. 4C600M40x2F600x2q4)	-	220	-	-	82	-
2000C	NSABP Protocol B-31	4A60C600q3; 4P175q3 / 12P80q1m ± Trast 1yr	-	872	-	-	730	-
2000E	FinHer / FBCG 00-01	3D(80/100); 3FEC vs 3Vb25×3; 3(F600E60C600)	-	626	-	-	230	-
2000F	MA.21 Canada	(6E120C830q2 vs. 4AC q3); 4P175q3 vs. 6(C75×14E60×2F500×2)q4	-	961	98	-	640	79
2000H	BCIRG 005 (GMA TAX301)	(4AC;4D100) vs. 6ADC q3	-	2005	-	-	507	-

2000L	NSABP Protocol B-34	Clodronate vs. Control	-	2371	-	-	661	-
2000U	AERO-B2000 France	4FEC; 4P175 vs. 6(F500E100C500)	-	478	-	-	121	-
2000X	HeCOG HE 10/00	((3E110;3P250)q2 vs. 4E83P187 q3); 3CMFq2	-	556	42	-	171	7
2000~	NCCTG / Intergroup N9831	4AC q3;12P80 q1 ± 12mths Trast q1 (Sequential or Concurrent)	-	1301	-	-	1165	-
2001=	HORG CT/01.04	4E90;4D75q3 vs. 6E75D75 q3	-	101	152	-	71	85
2001B	HERA / BIG 01-01	Chemo; 12 or 24mths Trast q3 vs. not	-	1993	34	-	1721	27
2001E	PACS 04 France	6E75D75 vs. 6(F500E100C500)	-	1998	-	-	534	-
2001F	TACT ICR-CTSU	4(F600E60C600); 4D100 vs 8(F600E60C600)	-	1257	-	-	724	-
2001M	BCIRG 006 (GMA TAX302)	(6D75cisP75/carbPAUC6)q3; Trast vs. 4AC q3;4D100q3 ± 12mths Trast	-	1323	-	-	1217	-
2001P	CALGB 49907 / MAC.1	6Cap2000x14 increased to 2500 q3 vs. 6CMFq4/4ACq3	-	133	153	-	39	75
2001Q	SBG 2000-1 / SBG CEF-60 Sweden	6F600E[75-90]C[900-1200] vs. 6F600E60C600	-	550	-	-	258	-
2001X	TEAM	Exe 5yr vs. (Tam 2.5-3yr; Exe to yr 5)	-	2996	928	-	-	-
2002D	GBG 42 / NNBC 3-Europe	3FEC; 3D100 vs. 6(F500E100C500)	-	112	1540	-	41	702
2002H	M.D. Anderson ID01-580	(4D75Cap2000x14q3 vs. 12P80q1);4FECq3	-	-	-	-	40	45
2002N	PACS 05 France	6 vs. 4 F500E100C500q3	-	490	444	-	291	159
2002W	USON 01062 / N017629	4ACq3;(4D75Cap1650x14q3 vs. 4D100q3)	-	1050	369	-	651	208
2002X	CALGB 40101	4 vs. 6(P80/175 vs. AC)q2	-	850	1522	-	497	682
2003)	GIM 6 / PROMISE	(neo/adj(C100x14M40x2F600x2)/?)±Trp q4	-	39	156	-	5	41
2003<	TEXT / IBCSG 25-02 / MAC.5 / BIG 03-02	Trip + (Exe vs Tam) 5yr	-	198	1779	-	3	10
2003E	SOFT / IBCSG 24-02 / MAC.4 / BIG 02-02	Tam 5yr vs. (Exe vs Tam) 5yr ± Trp/Ooph/Ov.Irr.	-	99	2376	-	3	26
2003G	MA.27 / IBCSG 30-04	(Exe vs. Ana) 5yr ± Celecoxib 3yr	-	1292	4666	-	-	-
2003Q	GIM 2 Italy	(4E90C600F600;4P175)[q2 vs. q3]	-	394	864	-	98	166

2003S	Elderly Breast Cancer-Docetaxel Adjuvant Study (ELDA)	4 to 6(D35x3 vs. C600M40F600x2) q4	-	17	130	-	8	30
2003U	GEICAM 2003-02	4FEC; 8(P100)q1 vs 6(F500E50C500)	-	130	499	-	134	378
2003W	AZURE / BIG 1-04	Zoledronic + Calcium/VitD vs. Calcium/VitD	-	-	-	-	126	304
2004=	SBG 2004-1 Phase II	(4EC;4D)q2 or (4E100C825;4D80)q2 6ADCq3	-	1	49	-	-	32
2004A	FinXX	3D60Cap1800x15q3;3C600E75Caps1800x15q3 v 3D80q3;3C600E75F600q3	-	246	780	-	85	202
2004B	ABCSG Study XVI / SALSA	Tam 5yr; Ana (5yr vs. 2yr)	-	441	2149	-	-	-
2004D	NSABP Protocol B-38	(4AC;4P175)q2 vs. 6ADCq3 vs. AC;4PGemq2	-	-	3160	-	-	789
2004F	GEICAM 2003-10	(4EC;4D100)q3 vs. (4E90D75;4Cap2500x14) q3	-	240	718	-	60	133
2004N	MATADOR / BOOG 2005-02 / CKTO 2004-04	4 vs. 6(D75A50C600q3 vs. A60C600q2)	-	3	251	-	2	50
2004X	GAIN / GBG 33	(4E112.5C600q2;10P67.5q1 4Cap2000x14q3 vs. 3E150q2;3P225q2;3C2500-2000q2)±Ibdx2yr	-	68	1893	-	21	598
2004]	HOBOE	(Trip + (Let vs. Tam) 5yr) ± Zoledronic	-	71	349	-	-	-
2005C	HeCOG HE 10/05	3E110q2; 3P200q2 ;3CMFq2 vs. 3E110q2;3C840M57F840q2;(9D35 vs. 9P80)q1	-	-	611	-	-	181
2005P	TACT2	4E100 [q2 vs. q3];4CMF q4 ± 4Cap2500x14 q3	-	-	2234	-	-	979
2006%	CIBOMA 2004-01 / GEICAM 2003-11	neo Anth/Tax ± 8Cap2000x14q3	-	-	-	-	-	500
2006L	TAILORx	Tam 5yr ± 4D75C600 (89%) or 4AC;4P175 or 4-6D75A50C500	-	-	4205	-	-	-
2006Z	EORTC 10041 / BIG 3-04 / MINDACT	6(D75Cap825) q3 vs. 6F(E/A)C	-	-	3016	-	-	378
2007$	ECOG E5103	4(AC+Bev)q2/3;12Pq1+4Bevq3 ±10Bev vs 4AC;12P	-	-	1544	-	-	1144
2007&	USO 06090 / 11271	6(D75C600A50 vs. D75C600) q3	-	-	812	-	-	365

2007B	SBG 2004-1 Phase III / ABCSG-25 / GBG 53 / PANTHER	(4E100C825;4D80)q2 vs. (3F500E100C500;3D100)q3	-	-	663	-	-	159
2007E	TEAM IIb/BOOG 2006-04b	Ibandronate vs. Control	-	-	289	-	-	-
2007R	AERAS NSAS-BC 05	Ana (10yr vs. 5yr)	-	-	48	-	-	-
2007S	FATA-GIM3	(Ana vs. Exe vs. Let 5yr) vs. Tam 2yr; (Ana vs. Exe vs. Let 3yr)	-	-	1297	-	-	-
2009$	GBG 52 / ICE II Phase II	6(NabP100Cap2000) q3 vs. 4(E90C600) q3/6(C500M40F600) q4	-	186	-	-	52	-
2009J	NSABP B-46-I	6(DC+Bev)q3;11Bev vs 6(D75C600) q3 vs. 6(D75A50C500) q3	-	-	-	-	499	-
2009M	WSG Plan B	4EC;4D100q3 vs. 6(D75C600) q3	-	-	382	-	-	105
2009X	TITAN / SCRA BRE145	4AC; (4Ix40 v 12P80) q3	-	-	-	-	-	185

Table S2: Main references for trials

Trial code	Trial name	Main reference
1979T	Umeå part of SESBCG-Örebro-Karlstad Study	Swedish Breast Cancer Cooperative Group. Randomized trial of two versus five years of adjuvant tamoxifen for postmenopausal early stage breast cancer. *J Natl Cancer Inst*. 1996; 88(21):1543-1549.
1982R	IKA C8209 Amsterdam	Vermorken JB, Burgers JMV, Taat CW, van de Slee PHTJ, Hennipman A, Nortier JWR, et al. Adjuvant tamoxifen in breast cancer: interim results of a Comprehensive Cancer Center Amsterdam (CCCA) trial. *Breast Cancer Res Treat*. 1998; 50(3):283, A329.
1984K	GUN-3 Naples	De Placido S, Perrone F, Carlomagno C, Morabito A, Pagliarulo C, Lauria R, et al. CMF vs alternating CMF/EV in the adjuvant treatment of operable breast cancer. A single centre randomised clinical trial (Naples GUN-3 study). *Br J Cancer*. 1995; 71(6):1283-1287.
1984Q	ABCSG Trial 1	Jakesz R, Samonigg H, Gnant M, Kubista E, Steindorfer P, Hausmaninger H, et al. Very low-dose adjuvant chemotherapy in steroid receptor negative stage I breast cancer patients. *Eur J Cancer*. 1998; 34(1):66-70.
1984R	DFCI Lumpectomy RT Seq Trial	Bellon JR, Come SE, Gelman RS, Henderson IC, Shulman LN, Silver BJ, et al. Sequencing of chemotherapy and radiation therapy in early-stage breast cancer: Updated results of a prospective randomized trial. *J Clin Oncol.* 2005; 23(9):1934-1940.
1985A	CALGB Study CLB-8541	Muss HB, Woolf S, Berry D, Cirrincione C, Weiss RB, Budman D, et al. Adjuvant chemotherapy in older and younger women with lymph node-positive breast cancer. *JAMA*. 2005; 293(9):1073-1081.
1985M	Bordeaux Patey Mastectomy Neo vs Adj Chemo	Mauriac L, MacGrogan G, Avril A, Durand M, Floquet A, Debled M, et al. Neoadjuvant chemotherapy for operable breast carcinoma larger than 3 cm: a unicentre randomized trial with a 124-month median follow-up. Institut Bergonie Bordeaux Groupe Sein (IBBGS). *Ann Oncol*. 1999; 10(1):47-52.
1986A	E.O.R.T.C. Trial 10854	Elkhuizen PH, van Slooten HJ, Clahsen PC, Hermans J, van de Velde CJ, van den Broek LC, et al. High local recurrence risk after breast-conserving therapy in node-negative premenopausal breast cancer patients is greatly reduced by one course of perioperative chemotherapy: A European Organization for Research and Treatment of Cancer Breast Cancer Cooperative Group Study. *J Clin Oncol*. 2000; 18(5):1075-1083.
1986H	IBCSG Trial VI	Pagani O, O'Neill A, Castiglione M, Gelber RD, Goldhirsch A, Rudenstam CM, et al. Prognostic impact of amenorrhoea after adjuvant chemotherapy in premenopausal breast cancer patients with axillary node involvement: results of the International Breast Cancer Study Group (IBCSG) Trial VI. *Eur J Cancer*. 1998; 34(5):632-640.<br><br>Karlsson P, Cole BF, Price KN, Gelber RD, Coates AS, Goldhirsch A, et al. Timing of Radiation Therapy and Chemotherapy After Breast-Conserving Surgery for Node-Positive Breast Cancer: Long-Term Results From International Breast Cancer Study Group Trials VI and VII. *Int J Radiat Oncol Biol Phys.*2016; 96(2):273-279.

1986L	M.D. Anderson Study 86-12	Assikis V, Buzdar A, Yang Y, Smith T, Theriault R, Booser D, et al. A phase III trial of sequential adjuvant chemotherapy for operable breast carcinoma: final analysis with 10-year follow-up. *Cancer*. 2003; 97(11):2716-2723.
1986P	French Adjuvant Study Group GFEA 01	Fumoleau P, Kerbrat P, Romestaing P, Fargeot P, Bremond A, Namer M, et al. Randomized trial comparing six versus three cycles of epirubicin-based adjuvant chemotherapy in premenopausal, node-positive breast cancer patients: 10-year follow-up results of the French Adjuvant Study Group 01 trial. *J Clin Oncol*. 2003; 21(2):298-305.
1987A	C.R.C. Under 50s Trial (part of `ZIPP')	Baum M, Hackshaw A, Houghton J, Rutqvist, Fornander T, Nordenskjold B, et al. Adjuvant goserelin in pre-menopausal patients with early breast cancer: Results from the ZIPP study. *Eur J Cancer*. 2006; 42:895-904.
1987C	N.S.A.B.C. Israel Mastectomy or Lumpectomy	Ron IG, Wigler N, Borovik R, Brufman G, Rizel S, Shani A, et al. CMF (cyclophosphamide, methotrexate, 5-fluorouracil) versus cnf (cyclophosphamide, mitoxantrone, 5-fluorouracil) as adjuvant chemotherapy for stage II lymph-node positive breast cancer: a phase III randomized multicenter study. *Am J Clin Oncol*. 2001; 24(4):323-327.
1988A	NSABP Protocol B-18	Wolmark N, Wang J, Mamounas E, Bryant J, Fisher B. Preoperative chemotherapy in patients with operable breast cancer: nine-year results from National Surgical Adjuvant Breast and Bowel Project B-18. *J Natl Cancer Inst Monogr*. 2001; (30):96-102.
1988B	NSABP Protocol B-19	Fisher B, Jeong JH, Anderson S, Wolmark N. Treatment of axillary lymph node-negative, estrogen receptor-negative breast cancer: updated findings from National Surgical Adjuvant Breast and Bowel Project clinical trials. *J Natl Cancer Inst*. 2004; 96(24):1823-1831.
1988D	IBCSG Trial IX	Aebi S, Sun Z, Braun D, Price KN, Castiglione-Gertsch M, Rabaglio M, et al. Differential efficacy of three cycles of CMF followed by tamoxifen in patients with ER-positive and ER-negative tumors: Long-term follow up on IBCSG Trial IX. *Ann Oncol*. 2011; 22(9):1981-1987
1988R	Jules Bordet Chemo High vs. Std	de Azambuja E, Paesmans M, Beauduin M, Vindevoghel A, Cornez N, Finet C, et al. Long-term benefit of high-dose epirubicin in adjuvant chemotherapy for node-positive breast cancer: 15-year efficacy results of the Belgian multicentre study. *J Clin Oncol*. 2009; 27(5):720-725.
1989&	Marsden Bisphosphonate Trial	Powles T, Paterson S, Kanis JA, McCloskey E, Ashley S, Tidy A, et al. Randomized, placebo-controlled trial of clodronate in patients with primary operable breast cancer. *J Clin Oncol*. 2002; 20(15):3219-3224.
1989@	Bari Chemotherapy Trial	Paradiso A, De Lena M, Sambiasi M, Mangia A, Brandi M, Schittulli F. Adjuvant hormonetherapy for slow proliferating node-negative breast cancer patients. Results of the phase III trial of NCl-Bari. *Breast*. 2003; 12 Suppl 1:S40, P90
1989B	SWOG 8814 / INT 0100	Albain KS, Barlow WE, Ravdin PM, Farrar WB, Burton GV, Ketchel SJ, et al. Adjuvant chemotherapy and timing of tamoxifen in postmenopausal patients with endocrine-responsive, node-positive breast cancer: a phase 3, open-label, randomised controlled trial. *Lancet*. 2009; 374(9707):2055-2063.

1989C	Gustave-Roussy FNCLCC Trial	Arriagada R, Le MG, Spielmann M, Mauriac L, Bonneterre J, Namer M, et al. Randomized trial of adjuvant ovarian suppression in 926 premenopausal patients with early breast cancer treated with adjuvant chemotherapy. *Ann Oncol*. 2005; 16(3):389-396
1989D	Gustave-Roussy Chemotherapy Trial	Arriagada R, Spielmann M, Koscielny S, Le Chevalier T, Delozier T, Ducourtieux M, et al. Patterns of failure in a randomized trial of adjuvant chemotherapy in postmenopausal patients with early breast cancer treated with tamoxifen. *Ann Oncol*. 2002; 13(9):1378-1386.
1989E	G.R.O.C.T.A. IV	Rubagotti A, Perrotta A, Casella C, Boccardo F. Risk of new primaries after chemotherapy and/or tamoxifen treatment for early breast cancer. *Ann Oncol*. 1996; 7:239-244.
1989F	ECOG EST 5188 / INT 0101	Davidson NE, O'Neill AM, Vukov AM, Osborne CK, Martino S, White DR, et al. Chemoendocrine therapy for premenopausal women with axillary lymph node-positive, steroid hormone receptor-positive breast cancer: results from INT 0101 (E5188). *J Clin Oncol*. 2005; 23(25):5973-5982.
1989G	ECOG EST 3189 = INT 0108	Fetting JH, Gray R, Fairclough DL, Smith TJ, Margolin KA, Citron ML, et al. Sixteen-week multidrug regimen versus cyclophosphamide, doxorubicin, and fluorouracil as adjuvant therapy for node-positive, receptor-negative breast cancer: an intergroup study. *J Clin Oncol*. 1998; 16(7):2382-2391.
1989M	NSABP Protocol B-22	Fisher B, Anderson S, Wickerham DL, DeCillis A, Dimitrov N, Mamounas E, et al. Increased intensification and total dose of cyclophosphamide in a doxorubicin-cyclophosphamide regimen for the treatment of primary breast cancer: findings from National Surgical Adjuvant Breast and Bowel Project B-22. *J Clin Oncol*. 1997; 15(5):1858-1869.
1989N	E.O.R.T.C. Trial 10882/22881	Bartelink H, Horiot JC, Poortmans P, Struikmans H, van den Bogaert W, Barillot I, et al. Impact of radiation dose on local control, fibrosis and survival after breast conserving treatment: 10 years results of the EORTC trial 22881-10882. *Breast Cancer Res Treat*. 2006; 100(Suppl 1):S8, A10.
1989P	DBCG 89c	Adjuvant one year tamoxifen versus two years tamoxifen in post-menopausal receptor positive/unknown high-risk (node-positive or T > 5 cm) patients. DBCG 89C. in: Piccart, M. J.; Goldhirsch, A., and on behalf of the Breast International Group . *An overview of recent and ongoing adjuvant clinical trials for breast cancer.* 2nd Edition ed. Breast International Group; 2000: 91.
1989W	Umeå part of DBCG89d-Umeå-Uppsala-Örebro Trial	Adjuvant therapy of premenopausal women with lymph node-positive breast cancer. DBCG 89 B + 89 D + CSB II-2. in: Piccart, M. J.; Goldhirsch, A., and on behalf of the Breast International Group. *An overview of recent and ongoing adjuvant clinical trials for breast cancer.* 2nd Edition ed. Breast International Group; 2000: 192-3.
1990*	GABG Germany Clodronate Trial	Diel IJ, Jaschke A, Solomayer EF, Gollan C, Bastert G, Sohn C, et al. Adjuvant oral clodronate improves the overall survival of primary breast cancer patients with micrometastases to the bone marrow - a long-term follow-up. *Ann Oncol*. 2008; 19(12):2007-2011.
1990@	Helsinki Toremifene Trial	Saarto T, Blomqvist C, Ehnholm C, Taskinen MR, Elomaa I. Antiatherogenic effects of adjuvant antiestrogens: a randomized trial comparing the effects of tamoxifen and toremifene on plasma lipid levels in postmenopausal women with node-positive breast cancer. *J Clin Oncol*. 1996; 14:429-433.
1990C	French Adjuvant Study Group GFEA 05	Bonneterre J, Roche H, Kerbrat P, Bremond A, Fumoleau P, Namer M, et al. Epirubicin increases long-term survival in adjuvant chemotherapy of patients with poor-prognosis, node-positive, early breast

		cancer: 10-year follow-up results of the French Adjuvant Study Group 05 randomized trial. *J Clin Oncol*. 2005; 23(12):2686-2693.
1990F	Zoladex Versus CMF (ZEBRA)	Kaufmann M, Jonat W, Blamey R, Cuzick J, Namer M, Fogelman I, et al. Survival analyses from the ZEBRA study: goserelin (Zoladex) versus CMF in premenopausal women with node-positive breast cancer. *Eur J Cancer*. 2003; 39(12):1711-1717.
1990K	Sto 6 Stockholm	Dar HA, Johansson A, Nordenskjöld A, Yau C, Benz C, Essermann L, et al. 20-year tamoxifen benefit in ER-positive/HER2-negative breast cancer patients in randomized clinical trials. *Ann Oncol.* 2022; 33:S153-S154 A168P.<br><br>Lofgren L, Wallberg B, Wilking N, Fornander T, Rutqvist LE, Carlstrom K, et al. Tamoxifen and megestrol acetate for postmenopausal breast cancer: diverging effects on liver proteins, androgens, and glucocorticoids. *Med Oncol*. 2004; 21(4):309-318.
1990L	Sto 7 Stockholm	Khoshnoud MR, Fornander T, Johansson H, Rutqvist L-E. Long-term pattern of disease recurrence among patients with early-stage breast cancer according to estrogen receptor status and use of adjuvant tamoxifen. *Breast Cancer Res Treat*. 2008; 107(1):71-78.<br><br>Dar HA, Johansson A, Nordenskjöld A, Yau C, Benz C, Essermann L, et al. 20-year tamoxifen benefit in ER-positive/HER2-negative breast cancer patients in randomized clinical trials. *Ann Oncol.* 2022; 33:S153-S154 A168P.
1990S	IBCSG Trial VIII	Karlsson P, Sun Z, Braun D, Price KN, Castiglione-Gertsch M, Rabaglio M, et al. Long-term results of International Breast Cancer Study Group Trial VIII: adjuvant chemotherapy plus goserelin compared with either therapy alone for premenopausal patients with node-negative breast cancer. *Ann Oncol*. 2011; 22(10):2216-2226.
1990W	ABCSG Study V	Jakesz R, Hausmaninger H, Kubista E, Gnant M, Menzel C, Bauernhofer T, et al. Randomized adjuvant trial of tamoxifen and goserelin versus cyclophosphamide, methotrexate, and fluorouracil: evidence for the superiority of treatment with endocrine blockade in premenopausal patients with hormone-responsive breast cancer--Austrian Breast and Colorectal Cancer Study Group Trial 5. *J Clin Oncol*. 2002; 20(24):4621-4627.
1990Z	GOIRC SANG 2	Colozza M, Sidoni A, Mosconi AM, Cavaliere A, Bisagni G, Gori S, et al. HER2 overexpression as a predictive marker in a randomized trial comparing adjuvant cyclophosphamide/methotrexate/5-fluorouracil with epirubicin in patients with stage I/II breast cancer: long-term results. *Clin Breast Cancer*. 2005; 6(3):253-259.
1991C	E.O.R.T.C. Trial 10901	Morales L, Canney P, Dyczka J, Rutgers E, Coleman R, Cufer T, et al. Postoperative adjuvant chemotherapy followed by adjuvant tamoxifen versus nil for patients with operable breast cancer: a randomised phase III trial of the European Organisation for Research and Treatment of Cancer Breast Group. *Eur J Cancer*. 2007; 43(2):331-340

1991H	NSABP Protocol B-23	Fisher B, Anderson S, Tan Chiu E, Wolmark N, Wickerham DL, Fisher ER, et al. Tamoxifen and chemotherapy for axillary node-negative, estrogen receptor-negative breast cancer: findings from National Surgical Adjuvant Breast and Bowel Project B-23. *J Clin Oncol*. 2001; 19(4):931-942.
1991P	SweBCG 91-RT	Sjöström M, Lundstedt D, Hartman L, Holmberg E, KIllander F, Kovács A, et al. Response to radiotherapy after breast-conserving surgery in different breast cancer subtypes in the Swedish Breast Cancer Group 91 Radiotherapy randomized clinical trial. *J Clin Oncol*. 2017; 35(28):3222-3229.
1991Q	G.O.C.S.I. MAM1	De Placido S, De Laurentiis M, De Lena M, Lorusso V, Paradiso A, D'Aprile M, et al. A randomised factorial trial of sequential doxorubicin and CMF vs CMF and chemotherapy alone vs chemotherapy followed by goserelin plus tamoxifen as adjuvant treatment of node-positive breast cancer. *Br J Cancer*. 2005; 92(3):467-474.
1992D	IKA C9203 Amsterdam	n/a
1992E	GONO-MIG 1 Genova	Blondeaux E, Lambertini M, Michelotti A, Conte B, Benasso M, Dellepiane C, et al. Dose-dense adjuvant chemotherapy in early breast cancer patients: 15-year results of the Phase 3 Mammella InterGruppo (MIG)-1 study. *Br J Cancer*. 2020; 122(11):1611-1617.
1992F	NSABP Protocol B-25	Fisher B, Anderson S, DeCillis A, Dimitrov N, Atkins JN, Fehrenbacher L, et al. Further evaluation of intensified and increased total dose of cyclophosphamide for the treatment of primary breast cancer: findings from National Surgical Adjuvant Breast and Bowel Project B-25. *J Clin Oncol*. 1999; 17(11):3374-3388.
1992N	C/9/91 / HMFEC London	Coombes RC, Kilburn LS, Tubiana-Mathieu N, Olmos T, Van Bochove A, Perez-Lopez FR, et al. Epirubicin dose and sequential hormonal therapy-Mature results of the HMFEC randomised phase III trial in premenopausal patients with node positive early breast cancer. *Eur J Cancer*. 2016; 60:146-153
1992Q	FBCG92-01 Finland	Tiitinen A, Nikander E, Hietanen P, Metsa Heikkila M, Ylikorkala O. Changes in bone mineral density during and after 3 years' use of tamoxifen or toremifene. *Maturitas*. 2004; 48(3):321-327.
1993B	MA.12 Canada	Bramwell VHC, Pritchard KI, Tu D, Tonkin K, Vachhrajani H, Vandenberg TA, et al. A randomized placebo-controlled study of tamoxifen after adjuvant chemotherapy in premenopausal women with early breast cancer (National Cancer Institute of Canada--Clinical Trials Group Trial, MA.12). *Ann Oncol*. 2010; 21:283-290.
1993C	GABG-4-A-93 Germany	von Minckwitz G, Graf E, Geberth M, Eiermann W, Jonat W, Conrad B, et al. CMF versus goserelin as adjuvant therapy for node-negative, hormone-receptor-positive breast cancer in premenopausal patien9ts: A randomised trial (GABG trial IV-A-93). *Eur J Cancer*. 2006; 42(12):1780-1788.
1993H	IBCSG Trial 11-93	Thürlimann B, Price KN, Gelber RD, Holmberg SB, Crivellari D, Colleoni M, et al. Is chemotherapy necessary for premenopausal women with lower-risk node-positive, endocrine responsive breast cancer?10-year update of International Breast Cancer Study Group Trial 11-93. *Breast Cancer Res Treat*. 2009; 113(1):137-144.
1993J	Fondazione Michelangelo High-Dose Therapy Trial	Gianni AM, Siena S, Bregni M, Di Nicola M, Orefice S, Cusumano F, et al. Efficacy, toxicity, and applicability of high-dose sequential chemotherapy as adjuvant treatment in operable breast cancer with 10 or more involved axillary nodes: five-year results. *J Clin Oncol*. 1997; 15(6):2312-2321.

1993L	IBCSG Trial 10-93	Rudenstam CM, Zahrieh D, Forbes JF, Crivellari D, Holmberg SB, Rey P, et al. Randomized trial comparing axillary clearance versus no axillary clearance in older patients with breast cancer: first results of International Breast Cancer Study Group Trial 10-93. *J Clin Oncol*. 2006; 24(3):337-344.
1993M	IBCSG Trial 12a-93	Pagani O, Gelber S, Simoncini E, Castiglione-Gertsch M, Price KN, Gelber RD, et al. Is adjuvant chemotherapy of benefit for postmenopausal women who receive endocrine treatment for highly endocrine-responsive, node-positive breast cancer? International Breast Cancer Study Group Trials VII and 12-93. *Breast Cancer Res Treat*. 2009; 116(3):491-500.
1993N	IBCSG 13-93	Colleoni M, Gelber S, Goldhirsch A, Aebi S, Castiglione Gertsch M, Price KN, et al. Tamoxifen after adjuvant chemotherapy for premenopausal women with lymph node-positive breast cancer: International Breast Cancer Study Group Trial 13-93. *J Clin Oncol*. 2006; 24(9):1332-1341.
1993P	IBCSG 14a-93	International Breast Cancer Study Group. Effects of a treatment gap during adjuvant chemotherapy in node-positive breast cancer: results of International Breast Cancer Study Group (IBCSG) Trials 13-93 and 14-93. *Ann Oncol*. 2007; 18(7):1177-1184.
1993Q	IBCSG 15-95	Colleoni M, Sun Z, Martinelli G, Basser RL, Coates AS, Gelber RD, et al. The effect of endocrine responsiveness on high-risk breast cancer treated with dose-intensive chemotherapy: results of International Breast Cancer Study Group Trial 15-95 after prolonged follow-up. *Ann Oncol*. 2009; 20(8):1344-1351.
1993S	Chemo-N0-Trial Germany	Harbeck N, Schmitt M, Meisner C, Friedel C, Untch M, Schmidt M, et al. Ten-year analysis of the prospective multicentre Chemo-N0 trial validates American Society of Clinical Oncology (ASCO)-recommended biomarkers uPA and PAI-1 for therapy decision making in node-negative breast cancer patients. *Eur J Cancer*. 2013; 49(8):1825-1835.
1993V	M93SCB	Steenbruggen TG, Steggink LC, Seynaeve CM, van der Hoeven JJM, Hooning MJ, Jager A, et al. High-dose chemotherapy with hematopoietic stem cell transplant in patients with high-risk breast cancer and 4 or more involved axillary lymph nodes. 20-year follow-up of a phase 3 randomized clinical trial. *JAMA Oncol*. 2020:Published online Jan 30, 2020.
1994B	MD Anderson Protocol 94-00	Albert JM, Buzdar AU, Guzman R, Allen PK, Strom EA, Perkins GH, et al. Prospective randomized trial of 5-fluorouracil, doxorubicin, and cyclophosphamide (FAC) versus paclitaxel and FAC (TFAC) in patients with operable breast cancer: impact of taxane chemotherapy on locoregional control. *Breast Cancer Res Treat*. 2011; 128(2):421-427.
1994C	C.A.L.G.B. Study 9343	Hughes KS, Schnaper LA, Bellon JR, Cirrincione CT, Berry DA, McCormick B, et al. Lumpectomy plus tamoxifen with or without irradiation in women age 70 years or older with early breast cancer: long-term follow-up of CALGB 9343. *J Clin Oncol*. 2013; 31(19):2382-2387.
1994D	C.A.L.G.B. Study 9344 / Intergroup 0148	Liu MC, Demetri GD, Berry DA, Norton L, Broadwater G, Robert NJ, et al. Dose-escalation of filgrastim does not improve efficacy: clinical tolerability and long-term follow-up on CALGB study 9141 adjuvant chemotherapy for node-positive breast cancer patients using dose-intensified doxorubicin plus cyclophosphamide followed by paclitaxel. *Cancer Treat Rev*. 2008; 34(3):223-230.

1994H	Huguenin FNCLCC Radiotherapy Trial	Rouëssé J, de la Lande B, Bertheault Cvitkovic F, Serin D, Graic Y, Combe M, et al. A phase III randomized trial comparing adjuvant concomitant chemoradiotherapy versus standard adjuvant chemotherapy followed by radiotherapy in operable node-positive breast cancer: final results. *Int J Radiat Oncol Biol Phys*. 2006; 64(4):1072-1080.
1994J	GOIRC SANG 2B R1	Conventional chemotherapy compared to experimental chemotherapy (rotational crossing CMFEV) as adjuvant treatment in patients with moderate-high-risk stage I or with stage II (1 to 9 positive nodes) breast carcinoma. SANG 2B. . in: Piccart, M. J.; Goldhirsch, A., and on behalf of the Breast International Group. *An overview of recent and ongoing adjuvant clinical trials for breast cancer.* 2nd Edition ed. Breast International Group; 2000:140-141.
1994R	PEGASE 01	Marino P, Roche H, Biron P, Janvier M, Spaeth D, Fabbro M, et al. Deterioration of quality of life of high-risk breast cancer patients treated with high-dose chemotherapy: The PEGASE 01 quality of life study. *Value in Health*. 2008; 11(4):709-718.
1994S	ECOG EST3193/INT0142	Tevaarwerk AJ, Wang M, Zhao F, Fetting JH, Cella D, Wagner LI, et al. Phase III comparison of tamoxifen versus tamoxifen plus ovarian function suppression in premenopausal women with node-negative, hormone receptor–positive breast cancer (E-3193, INT-0142): a trial of the Eastern Cooperative Oncology Group. *J Clin Oncol*. 2014; 32(35):3948-3958.
1995K	NSABP Protocol B-28	Mamounas EP, Bryant J, Lembersky B, Fehrenbacher L, Sedlacek SM, Fisher B, et al. Paclitaxel after doxorubicin plus cyclophosphamide as adjuvant chemotherapy for node-positive breast cancer: results from NSABP B-28. *J Clin Oncol*. 2005; 23(16):3686-3696.
1995M	GEICAM 9401	Pico C, Martin M, Jara C, Barnadas A, Pelegri A, Balil A, et al. Epirubicin-cyclophosphamide adjuvant chemotherapy plus tamoxifen administered concurrently versus sequentially: randomized phase III trial in postmenopausal node-positive breast cancer patients. A GEICAM 9401 study. *Ann Oncol*. 2004; 15(1):79-87.
1995R	ECOG E-EB193 / INT-0151	Cobleigh MA, Gray R, Graham M, Norton L, Martino S, Budd GT, et al. Fenretinide (FEN) vs placebo in postmenopausal breast cancer patients receiving adjuvant tamoxifen (TAM), an Eastern Cooperative Oncology Group phase III Intergroup trial (EB193, INT-0151). *Proc Annu Meet Am Soc Clin Oncol*. 2000; 19:86a, A328.
1995S	Protocol CNR-9502 Milan	Martelli G, Boracchi P, Orenti A, Lozza L, Maugeri I, Vetrella G, et al. Axillary dissection versus no axillary dissection in older T1N0 breast cancer patients: 15-year results of trial and out-trial patients. *European Journal of Surgical Oncology*. 2014; 40(7):805-812.
1995T	HORG Docetaxel Trial	Polyzos A, Malamos N, Boukovinas I, Adamou A, Ziras N, Kalbakis K, et al. FEC versus sequential docetaxel followed by epirubicin/cyclophosphamide as adjuvant chemotherapy in women with axillary node-positive early breast cancer: a randomized study of the Hellenic Oncology Research Group (HORG). *Breast Cancer Res Treat*. 2010; 119(1):95-104.
1996A	NEAT Trial UCRI	Earl HM, Hiller L, Dunn JA, Vallier AL, Bowden SJ, Jordan SD, et al. Adjuvant epirubicin followed by cyclophosphamide, methotrexate and fluorouracil (CMF) vs CMF in early breast cancer: results with

		over 7 years median follow-up from the randomised phase III NEAT/BR9601 trials. *Br J Cancer.* 2012; 107(8):1257-1267.
1996E	ABCSG Study IX	Jakesz R, Samonigg H, Gnant M, Kubista E, Depisch D, Kolb R, et al. Significant increase in breast conservation in 16 years of trials conducted by the Austrian Breast & Colorectal Cancer Study Group. *Ann Surg*. 2003; 237(4):556-564.
1996J	ATAC Trial CRUK	Cuzick J, Sestak I, Baum M, Buzdar A, Howell A, Dowsett M, et al. Effect of anastrozole and tamoxifen as adjuvant treatment for early-stage breast cancer: 10-year analysis of the ATAC trial. *Lancet Oncol*. 2010; 11(12):1135-1141.
1996R	HCFU Trial Japan	Tominaga T, Kimura M, Asaga T, Yoshida M, Awane H, Koyama H, et al. 1-hexylcarbamoyl-5-fluorouracil + cyclophosphamide + tamoxifen versus CMF + tamoxifen in women with lymph node-positive breast cancer after primary surgery: a randomized controlled trial. *Oncol Rep*. 2004; 12(4):797-803.
1996U	ARCOSEIN Study	Toledano A, Azria D, Garaud P, Fourquet A, Serin D, Bosset JF, et al. Phase III trial of concurrent or sequential adjuvant chemoradiotherapy after conservative surgery for early-stage breast cancer: final results of the ARCOSEIN trial. *J Clin Oncol*. 2007; 25(4):405-410.
1996W	ECTO Milan	Gianni L, Baselga J, Eiermann W, Porta VG, Semiglazov V, Lluch A, et al. Phase III trial evaluating the addition of paclitaxel to doxorubicin followed by cyclophosphamide, methotrexate, and fluorouracil, as adjuvant or primary systemic therapy: European Cooperative Trial in Operable Breast Cancer. *J Clin Oncol*. 2009; 27(15):2474-2481.
1996X	GONO-MIG 5	Del Mastro L, Levaggi A, Michelotti A, Cavazzini G, Adami F, Scotto T, et al. 5-Fluorouracil, epirubicin and cyclophosphamide versus epirubicin and paclitaxel in node-positive early breast cancer: a phase-III randomized GONO-MIG5 trial. *Breast Cancer Res Treat*. 2016; 155(1):117-126.
1996Y	ABCSG Trial VIII	Bago-Horvath Z, Rudas M, Dubsky P, Jakesz R, Singer CF, Kemmerling R, et al. Adjuvant sequencing of tamoxifen and anastrozole is superior to tamoxifen alone in postmenopausal women with low proliferating breast cancer. *Clin Cancer Res*. 2011; 17(24):7828-7834.
1997A	C/14/96 DEVA ICCG	Coombes RC, Bliss JM, Espie M, Erdkamp F, Wals J, Tres A, et al. Randomized, phase III trial of sequential epirubicin and docetaxel versus epirubicin alone in postmenopausal patients with node-positive breast cancer. *J Clin Oncol*. 2011; 29(24):3247-3254.
1997D	C.A.L.G.B. Study 9741 / Intergroup C9741	Citron ML, Berry DA, Cirrincione C, Hudis C, Winer EP, Gradishar WJ, et al. Randomized trial of dose-dense versus conventionally scheduled and sequential versus concurrent combination chemotherapy as postoperative adjuvant treatment of node-positive primary breast cancer: first report of Intergroup Trial C9741/Cancer and Leukemia Group B Trial 9741. *J Clin Oncol*. 2003; 21(8):1431-1439.
1997J	PACS 01 France	Coudert B, Asselain B, Campone M, Spielmann M, Machiels JP, Penault-Llorca F, et al. Extended benefit from sequential administration of docetaxel after standard fluorouracil, epirubicin, and cyclophosphamide regimen for node-positive breast cancer: the 8-year follow-up results of the UNICANCER-PACS01 trial. *Oncologist*. 2012; 17(7):900-909.

1997L	BCIRG 001 / GEICAM 9703 (RP56976-V-316)	Mackey JR, Martin M, Pienkowski T, Rolski J, Guastalla JP, Sami A, et al. Adjuvant docetaxel, doxorubicin, and cyclophosphamide in node-positive breast cancer: 10-year follow-up of the phase 3 randomised BCIRG 001 trial. *Lancet Oncol*. 2013; 14(1):72-80.
1997N	USON Trial 97-35	Jones S, Holmes FA, O'Shaughnessy J, Blum JL, Vukelja SJ, McIntyre KJ, et al. Docetaxel with cyclophosphamide is associated with an overall survival benefit compared with doxorubicin and cyclophsphamide: 7-year follow-up of US Oncology Research Trial 9735. *J Clin Oncol*. 2009; 27:1177-1183.
1997R	HeCOG HE 1097	Fountzilas G, Skarlos D, Dafni U, Gogas H, Briasoulis E, Pectasides D, et al. Postoperative dose-dense sequential chemotherapy with epirubicin, followed by CMF with or without paclitaxel, in patients with high-risk operable breast cancer: a randomized phase III study conducted by the Hellenic Cooperative Oncology Group. *Ann Oncol*. 2005; 16:1762-1771.
1997U	IBIS 03 Italy	Amadori D, Silvestrini R, De Lena M, Boccardo F, Rocca A, Scarpi E, et al. Randomized phase III trial of adjuvant epirubicin followed by cyclophosphamide, methotrexate, and 5-fluorouracil (CMF) versus CMF followed by epirubicin in patients with node-negative or 1–3 node-positive rapidly proliferating breast cancer. *Breast Cancer Res Treat*. 2011; 125:775-784.
1997V	IBIS 02 Italy	Boccardo F, Amadori D, Guglielmini P, Sismondi P, Farris A, Agostara B, et al. Epirubicin followed by cyclophosphamide, methotrexate and 5-fluorouracil versus paclitaxel followed by epirubicin and vinorelbine in patients with high-risk operable breast cancer. *Oncology*. 2010; 78(3-4):274-281.
1998@	NAFTA USA	Lewis JD, Chagpar AB, Shaughnessy EA, Nurko J, McMasters K, Edwards MJ. Excellent outcomes with adjuvant toremifene or tamoxifen in early stage breast cancer. *Cancer*. 2010; 116(10):2307-2315.
1998D	BIG 02-98 / TAX 315 / GEICAM 9803	Sonnenblick A, Francis PA, Azim HAJ, de Azambuja E, Nordenskjöld B, Gutiérez J, et al. Final 10-year results of the Breast International Group 2-98 phase III trial and the role of Ki67 in predicting benefit of adjuvant docetaxel in patients with ostrogen receptor positive breast cancer. *Eur J Cancer*. 2015; 51:1481-1489.
1998K	MD Anderson Protocol 98-240	Green MC, Buzdar AU, Smith T, Ibrahim NK, Valero V, Rosales M, et al. Weekly (wkly) paclitaxel (P) followed by FAC as primary systemic chemotherapy (PSC) of operable breast cancer improves pathologic complete remission (pCR) rates when compared to every 3-week (Q 3 wk) P therapy (tx) followed by FAC - final results of a prospective phase III randomized trial. *Proc Annu Meet Am Soc Clin Oncol*. 2002:35a, A135.
1998M	IBCSG 18-98 / BIG 01-98 / FEMTA	Regan MM, Neven P, Giobbie-Hurder A, Goldhirsch A, Ejlertsen B, Mauriac L, et al. Assessment of letrozole and tamoxifen alone and in sequence for postmenopausal women with steroid hormone receptor-positive breast cancer: the BIG 1-98 randomised clinical trial at 8.1 years median follow-up. *Lancet Oncol*. 2011; 12(12):1101-1108.
1998T	ECOG EST2197	Goldstein LJ, O'Neill A, Sparano JA, Perez EA, Shulman LN, Martino S, et al. Concurrent doxorubicin plus docetaxel is not more effective than concurrent doxorubicin plus cyclophosphamide in operable breast cancer with 0 to 3 positive axillary nodes: North American Breast Cancer Intergroup trial E 2197. *J Clin Oncol*. 2008; 26(25):4092-4099.

1999%	GEICAM 9805 / TARGET 0	Martin M, Lluch A, Segui MA, Ruiz A, Ramos M, Adrover E, et al. Toxicity and health-related quality of life in breast cancer patients receiving adjuvant docetaxel, doxorubicin, cyclophosphamide (TAC) or 5-fluorouracil, doxorubicin and cyclophosphamide (FAC): impact of adding primary prophylactic granulocyte-colony stimulating factor to the TAC regimen. *Ann Oncol*. 2006; 17(8):1205-1212
1999G	NSABP Protocol B-30	Ganz PA, Land SR, Geyer CEJ, Cecchini RS, Costantino JP, Pajon ER, et al. Menstrual history and quality-of-life outcomes in women with node-positive breast cancer treated with adjuvant therapy on the NSABP B-30 trial. *J Clin Oncol*. 2011; 29(9):1110-1116.
1999K	GEICAM 9906	Martin M, Rodriguez-Lescure A, Ruiz A, Alba E, Calvo L, Ruiz-Borrego M, et al. Randomized phase 3 trial of fluorouracil, epirubicin, and cyclophosphamide alone or followed by Paclitaxel for early breast cancer. *J Natl Cancer Inst*. 2008; 100(11):805-814.
1999N	RAPP-01 France	Brain EG, Debled M, Eymard J, Bachelot T, Extra J, Serin D, et al. Final results of the RAPP-01 phase III trial comparing doxorubicin and docetaxel with doxorubicin and cyclophosphamide in the adjuvant treatment of high-risk node negative and limited node positive (<=3) breast cancer patients. *Cancer Res*. 2009; 69(2 Suppl):A4101.
1999V	ECOG E1199/Intergroup	Sparano JA, Zhao F, Martino F, Ligibel JA, Perez EA, Saphner T, et al. Long-term follow-up of the E1199 phase III trial evaluating the role of taxane and schedule in operable breast cancer. *J Clin Oncol*. 2015; 33(21):2353-2360.
1999W	PRIME Scotland	Prescott RJ, Kunkler IH, Williams LJ, King CC, Jack W, Dixon JM, et al. Post-operative radiotherapy (RT) in minimum-risk elderly (PRIME) assessing the impact of breast radiotherapy on quality of life in low risk older patients. *Eur J Cancer Suppl*. 2007; 5(3):6-7, O-19.
2000=	USON Trial 99-016	Loesch D, Greco FA, Senzer NN, Burris HA, Hainsworth JD, Jones S, et al. Phase III multicenter trial of doxorubicin plus cyclophosphamide followed by paclitaxel compared with doxorubicin plus paclitaxel followed by weekly paclitaxel as adjuvant therapy for women with high-risk breast cancer. *J Clin Oncol*. 2010; 28(18):2958-2965.
2000A	Fondazione Michelangelo GMB/99/02	Moliterni A, Mansutti M, Aldrighetti D, Merlini L, Zuccarino L, Bari M, et al. Anthracycline-based sequential adjuvant chemotherapy in operable breast cancer: Five-year results of a randomized study by the Michelangelo Foundation. *J Clin Oncol*. 2007; 25(18_suppl):11s, A535.
2000C	NSABP Protocol B-31	Gavin PG, Song N, Kim SR, Lipchik C, Johnson NL, H. B, et al. Association of polymorphisms in FCGR2A and FCGR3A with degree of trastuzumab benefit in the adjuvant treatment of ERBB2/HER2-positive breast cancer: analysis of the NSABP B-31 trial. *JAMA Oncol*. 2017; 3(3):335-341.
2000E	FinHer / FBCG 00-01	Joensuu H, Bono P, Kataja V, Alanko T, Kokko R, Asola R, et al. Fluorouracil, epirubicin, and cyclophosphamide with either docetaxel or vinorelbine, with or without trastuzumab, as adjuvant treatments of breast cancer: final results of the FinHer Trial. *J Clin Oncol*. 2009; 27(34):5685-5692.
2000F	MA.21 Canada	Burnell M, Levine MN, Chapman J-AW, Bramwell V, Gelmon K, Walley B, et al. Cyclophosphamide, epirubicin, and fluorouracil versus dose-dense epirubicin and cyclophosphamide followed by paclitaxel versus doxorubicin and cyclophosphamide followed by paclitaxel in node-positive or high-risk node-negative breast cancer. *J Clin Oncol*. 2010; 28(1):77-82.

2000H	BCIRG 005 (GMA TAX301)	Mackey JR, Pienkowski T, Crown J, Sadeghi S, Martin M, Chan A, et al. Long-term outcomes after adjuvant treatment of sequential versus combination docetaxel with doxorubicin and cyclophosphamide in node-positive breast cancer: BCIRG-005 randomized trial. *Ann Oncol*. 2016; 27(6):1041-1047.
2000L	NSABP Protocol B-34	Paterson AH, Anderson SJ, Lembersky BC, Fehrenbacher L, Falkson CI, King KM, et al. Oral clodronate for adjuvant treatment of operable breast cancer (National Surgical Adjuvant Breast and Bowel Project protocol B-34): a multicentre, placebo-controlled, randomised trial. *Lancet Oncol*. 2012; 13(7):734-742.
2000U	AERO-B2000 France	Delbaldo C, Serin D, Mousseau M, Greget S, Audhuy B, Priou F, et al. A phase III adjuvant randomised trial of 6 cycles of 5-fluorouracil-epirubicine-cyclophosphamide (FEC100) versus 4 FEC 100 followed by 4 Taxol (FEC-T) in node positive breast cancer patients (Trial B2000). *Eur J Cancer*. 2014; 50(1):23-30.
2000X	HeCOG HE 10/00	Gogas H, Dafni U, Karina M, Papadimitriou C, Batistatou A, Bobos M, et al. Postoperative dose-dense sequential versus concomitant administration of epirubicin and paclitaxel in patients with node-positive breast cancer: 5-year results of the Hellenic Cooperative Oncology Group HE 10/00 phase III Trial. *Breast Cancer Res Treat*. 2012; 132(2):609-619.
2000~	NCCTG / Intergroup N9831	Romond EH, Perez EA, Bryant J, Suman VJ, Geyer CE, Davidson NE, et al. Trastuzumab plus adjuvant chemotherapy for operable HER2-positive breast cancer. *N Engl J Med*. 2005; 353:1673-1684.
2001=	HORG CT/01.04	Mavroudis D, Saloustros E, Boukovinas I, Papakotoulas P, Kakolyris S, Ziras N, et al. Sequential vs concurrent epirubicin and docetaxel as adjuvant chemotherapy for high-risk, node-negative, early breast cancer: an interim analysis of a randomised phase III study from the Hellenic Oncology Research Group. *Br J Cancer*. 2017; 117(2):164-170.
2001B	HERA / BIG 01-01	Cameron D, Piccart-Gebhart MJ, Gelber RD, Procter M, Goldhirsch A, de Azambuja E, et al. 11 years-follow-up of trastuzumab after adjuvant chemotherapy in HER2-positive early breast cancer: final analysis of the HERceptin Adjuvant (HERA) trial. *Lancet*. 2017; 389:1195-1205.
2001E	PACS 04 France	Spielmann M, Roche H, Delozier T, Canon JL, Romieu G, Bourgeois H, et al. Trastuzumab for patients with axillary-node-positive breast cancer: results of the FNCLCC-PACS 04 trial. *J Clin Oncol*. 2009; 27(36):6129-6134.
2001F	TACT ICR-CTSU	Ellis P, Barrett-Lee P, Johnson L, Cameron D, Wardley A, O'Reilly S, et al. Sequential docetaxel as adjuvant chemotherapy for early breast cancer (TACT): an open-label, phase III, randomised controlled trial. *Lancet*. 2009; 373:1681-1692
2001M	BCIRG 006 (GMA TAX302)	Slamon D, Eiermann W, Robert N, Pienkowski T, Martin M, Press M, et al. Adjuvant trastuzumab in HER2-positive breast cancer. *N Engl J Med*. 2011; 365(14):1273-1283.
2001P	CALGB 49907 / MAC.1	Muss HB, Polley M-Y, Berry DA, Liu H, Cirrincione CT, Theodoulou M, et al. Randomized trial of standard adjuvant chemotherapy regimens versus capecitabine in older women with early breast cancer: 10-year update of the CALGB 49907 trial. *J Clin Oncol*. 2019; 37(26):2338-2348.
2001Q	SBG 2000-1 / SBG CEF-60 Sweden	Lindman H, Andersson M, Ahlgren J, Balslev E, Sverrisdottir A, Holmberg SB, et al. A randomised study of tailored toxicity-based dosage of fluorouracil-epirubicin-cyclophosphamide chemotherapy for early breast cancer (SBG 2000-1). *Eur J Cancer*. 2018; 94:79-86

2001X	TEAM	Derks MGM, Blok EJ, Seynaeve C, Nortier JWR, Meershoek-Klein Kranenberg E, Liefers G-J, et al. Adjuvant tamoxifen and exemestane in women with postmenopausal early breast cancer (TEAM): 10-year follow-up of a multicentre, open-label, randomised, phase 3 trial. *Lancet Oncol*. 2017; 18:1211-1220.
2002D	GBG 42 / NNBC 3-Europe	Thomssen C, Vetter M, Kantelhardt EJ, Meisner C, Schmidt M, Martin PM, et al. Adjuvant docetaxel in node-negative breast cancer patients: a randomized trial of AGO-Breast Study Group, German Breast Group, and EORTC-Pathobiology Group. *Cancers*. 2023; 15(5):1580.
2002H	M.D. Anderson ID01-580	Kelly CM, Green MC, Broglio K, Thomas ES, Brewster AM, Valero V, et al. Phase III trial evaluating weekly paclitaxel versus docetaxel in combination with capecitabine in operable breast cancer. *J Clin Oncol*. 2012; 30(9):930-935.
2002N	PACS 05 France	Kerbrat P, Desmoulins I, Roca L, Levy C, Lortholary A, Marre A, et al. Optimal duration of adjuvant chemotherapy for high-risk node-negative (N-) breast cancer patients: 6-year results of the prospective randomised multicentre phase III UNICANCER-PACS 05 trial (UCBG-0106). *Eur J Cancer*. 2017; 79:166-175.
2002W	USON 01062 / N017629	O'Shaughnessy J, Koeppen H, Xiao Y, Lackner MR, Paul D, Stokoe C, et al. Patients with slowly proliferative early breast cancer have low five-year recurrence rates in a phase III adjuvant trial of capecitabine. *Clin Cancer Res*. 2015; 21(19):4305-4311.
2002X	CALGB 40101	Shulman LN, Berry DA, Cirrincione CT, Becker HP, Perez EA, O'Regan R, et al. Comparison of doxorubicin and cyclophosphamide versus single-agent paclitaxel as adjuvant therapy for breast cancer in women with 0 to 3 positive axillary nodes: CALGB 40101 (Alliance). *J Clin Oncol.* 2014; 32(22):2311-2317.
2003)	GIM 6 / PROMISE	Lambertini M, Boni L, Michelotti A, Gamucci T, Scotto T, Gori S, et al. Ovarian suppression with triptorelin during adjuvant breast cancer chemotherapy and long-term ovarian function, pregnancies, and disease-free survival: a randomized clinical trial. *JAMA*. 2015; 314(24):2632-2640.
2003<	TEXT / IBCSG 25-02 / MAC.5 / BIG 03-02	Pagani O, Francis PA, Fleming GF, Walley BA, Viale G, Colleoni M, et al. Absolute improvements in freedom from distant recurrence to tailor adjuvant endocrine therapies for premenopausal women: results from TEXT and SOFT. *J Clin Oncol*. 2020; 38(12):1293-1303.
2003E	SOFT / IBCSG 24-02 / MAC.4 / BIG 02-02	Pagani O, Francis PA, Fleming GF, Walley BA, Viale G, Colleoni M, et al. Absolute improvements in freedom from distant recurrence to tailor adjuvant endocrine therapies for premenopausal women: results from TEXT and SOFT. *J Clin Oncol*. 2020; 38(12):1293-1303.
2003G	MA.27 / IBCSG 30-04	Goss PE, Ingle JN, Pritchard KI, Ellis MJ, Sledge GW, Budd GT, et al. Exemestane versus anastrozole in postmenopausal women with early breast cancer: NCIC CTG MA.27—a randomized controlled phase III trial. *J Clin Oncol.* 2013; 31(11):1398-1404.
2003Q	GIM 2 Italy	Del Mastro L, Poggio F, Blondeaux E, De Placido S, Giuliano M, Forestieri V, et al. Fluorouracil and dose-dense adjuvant chemotherapy in patients with early-stage breast cancer (GIM2): end-of-study results from a randomised, phase 3 trial. *Lancet Oncol*. 2022:Published online Nov 09, 2022.

2003S	Elderly Breast Cancer-Docetaxel Adjuvant Study (ELDA)	Perrone F, Nuzzo F, Di Rella F, Gravina A, Iodice G, Labonia V, et al. Weekly docetaxel versus CMF as adjuvant chemotherapy for older women with early breast cancer: final results of the randomized phase III ELDA trial. *Ann Oncol.* 2015; 26(4):675-682.
2003U	GEICAM 2003-02	Martin M, Ruiz A, Ruiz Borrego M, Barnadas A, Gonzalez S, Calvo L, et al. Fluorouracil, doxorubicin, and cyclophosphamide (FAC) versus FAC followed by weekly paclitaxel as adjuvant therapy for high-risk, node-negative breast cancer: results from the GEICAM/2003-02 study. *J Clin Oncol.* 2013; 31(20):2593-2599.
2003W	AZURE / BIG 1-04	Coleman R, Cameron D, Dodwell D, Bell R, Wilson C, Rathbone E, et al. Adjuvant zoledronic acid in patients with early breast cancer: final efficacy analysis of the AZURE (BIG 01/04) randomised open-label phase 3 trial. *Lancet Oncology*. 2014; 15(9):997-1006.
2004=	SBG 2004-1 Phase II	Matikas A, Margolin S, Hellstrom M, Johansson H, O. Bengtsson N, Karlsson L, et al. Long-term safety and survival outcomes from the Scandinavian Breast Group 2004-1 randomized phase II trial of tailored dose-dense adjuvant chemotherapy for early breast cancer. *Breast Cancer Res Treat*. 2018; 168(2):349-355.
2004A	FinXX	Joensuu H, Kellokumpu-Lehtinen PL, Huovinen R, Jukkola-Vuorinen A, Tanner M, Kokko R, et al. Adjuvant capecitabine, docetaxel, cyclophosphamide, and epirubicin for early breast cancer: final analysis of the randomized FinXX trial. *J Clin Oncol.* 2012; 30(1):11-18.
2004B	ABCSG Study XVI / SALSA	Gnant M, Steger G, Greil R, Fitzal F, Mlineritsch B, Manfreda D, et al. A prospective randomized multi-center phase-III trial of additional 2 versus additional 5 years of anastrozole after initial 5 years of adjuvant endocrine therapy – results from 3,484 postmenopausal women in the ABCSG-16 trial. *Cancer Res*. 2018; 78(4 Suppl):GS3-01.
2004D	NSABP Protocol B-38	Swain SM, Tang G, Geyer CE, Jr., Rastogi P, Atkins JN, Donnellan PP, et al. Definitive results of a phase III adjuvant trial comparing three chemotherapy regimens in women with operable, node-positive breast cancer: the NSABP B-38 trial. *J Clin Oncol.* 2013; 31(26):3197-3204.
2004F	GEICAM 2003-10	Martín M, Ruiz Simón A, Ruiz Borrego M, Ribelles N, Rodríguez-Lescure A, Muñoz-Mateu M, et al. Epirubicin plus cyclophosphamide followed by docetaxel versus epirubicin plus docetaxel followed by capecitabine as adjuvant therapy for node-positive early breast cancer: results from the GEICAM/2003-10 study. *J Clin Oncol*. 2015; 33(32):3788-3795.
2004N	MATADOR / BOOG 2005-02 / CKTO 2004-04	van Rossum AGJ, M. K, van Werkhoven E, Opdam M, Mandjes IAM, van Leeuwen-Stok AE, et al. Adjuvant dose-dense doxorubicin-cyclophosphamide versus docetaxel-doxorubicin-cyclophosphamide for high-risk breast cancer: First results of the randomised MATADOR trial (BOOG 2004-04). *Eur J Cancer*. 2018; 102:40-48.
2004X	GAIN / GBG 33	Mobus V, von Minckwitz G, Jackisch C, Luck HJ, Schneeweiss A, Tesch H, et al. German Adjuvant Intergroup Node-positive Study (GAIN): a phase III trial comparing two dose-dense regimens (iddEPC versus ddEC-PwX) in high-risk early breast cancer patients. *Ann Oncol*. 2017; 28(8):1803-1810.

2004]	HOBOE	Perrone F, De Laurentiis M, De Placido S, Orditura M, Cinieri S, Riccardi F, et al. Adjuvant zoledronic acid and letrozole plus ovarian function suppression in premenopausal breast cancer: HOBOE phase 3 randomised trial. *Eur J Cancer*. 2019; 118:178-186.
2005C	HeCOG HE 10/05	Fountzilas G, Dafni U, Papadimitriou C, Timotheadou E, Gogas H, Eleftheraki AG, et al. Dose-dense sequential adjuvant chemotherapy followed, as indicated, by trastuzumab for one year in patients with early breast cancer: first report at 5-year median follow-up of a Hellenic Cooperative Oncology Group randomized phase III trial. *BMC Cancer*. 2014; 14:515.
2005P	TACT2	Cameron D, Morden JP, Canney P, Velikova G, Coleman R, Bartlett J, et al. Accelerated versus standard epirubicin followed by cyclophosphamide, methotrexate, and fluorouracil or capecitabine as adjuvant therapy for breast cancer in the randomised UK TACT2 trial (CRUK/05/19): a multicentre, phase 3, open-label, randomised, controlled trial. *Lancet Oncol*. 2017; 18(7):929-945.
2006%	CIBOMA 2004-01 / GEICAM 2003-11	Lluch A, Barrios CH, Torrecillas L, Ruiz-Borrego M, Bines J, Segalla J, et al. Phase III trial of adjuvant capecitabine after standard neo-/adjuvant chemotherapy in patients with early triple-negative breast cancer (GEICAM/2003-11_CIBOMA/2004-01). *J Clin Oncol*. 2020; 38(3):203-213.
2006L	TAILORx	Sparano J, Gray R, Zujewski JA, Makower D, Pritchard K, Albain K, et al. Prospective trial of endocrine therapy alone in patients with estrogen-receptor positive, HER2-negative, node-negative breast cancer: Results of the TAILORx low risk registry. *Eur J Cancer*. 2015; 51:S714.
2006Z	EORTC 10041 / BIG 3-04 / MINDACT	Delaloge S, Piccart M, Rutgers E, Litière S, van 't Veer LJ, van den Berkmortel F, et al. Standard anthracycline based versus docetaxel-capecitabine in early high clinical and/or genomic risk breast cancer in the EORTC 10041/BIG 3-04 MINDACT phase III trial. *J Clin Oncol*. 2020; 38(11):1186-97
2007$	ECOG E5103	Miller KD, O'Neill A, Gradishar W, Hobday TJ, Goldstein LJ, Mayer IA, et al. Double-blind phase III trial of adjuvant chemotherapy with and without bevacizumab in patients with lymph node-positive and high-risk lymph node-negative breast cancer (E5103). *J Clin Oncol*. 2018; 36(25):2621-2629.
2007&	USO 06090 / 11271	Blum JL, Flynn PJ, Yothers G, Asmar L, Geyer CE, Jr., Jacobs SA, et al. Anthracyclines in early breast cancer: the ABC Trials--USOR 06-090, NSABP B-46-I/USOR 07132, and NSABP B-49 (NRG Oncology). *J Clin Oncol*. 2017; 35(23):2647-2655.
2007B	SBG 2004-1 Phase III / ABCSG-25 / GBG 53 / PANTHER	Foukakis T, von Minckwitz G, Bengtsson N-O, Brandberg Y, Wallberg B, Fornander T, et al. Effect of tailored dose-dense chemotherapy vs standard 3-weekly adjuvant chemotherapy on recurrence-free survival among women with high-risk early breast cancer. A randomized trial. *JAMA*. 2016; 316(18):1888-1896.
2007E	TEAM IIb/BOOG 2006-04b	Vliek SB, Meershoek-Klein Kranenbarg E, van Rossum AGJ, Tanis BC, Putter H, van der Velden AWG, et al. The efficacy and safety of the addition of ibandronate to adjuvant hormonal therapy in postmenopausal women with hormone-receptor positive early breast cancer. First results of the TEAM IIB trial (BOOG 2006-04). *Cancer Res*. 2017; 77(4 suppl.):S6-02
2007R	AERAS NSAS-BC 05	Iwase T, Saji S, Iijima K, Higaki K, Ohtani S, Sato Y, et al. Postoperative adjuvant anastrozole for 10 or 5 years in patients with hormone receptor–positive breast cancer: AERAS, a randomized multicenter open-label phase III trial. *J Clin Oncol*. 2023: 41(18):3329-3338.

2007S	FATA-GIM3	De Placido S, Gallo C, De Laurentiis M, Bisagni G, Arpino G, Sarobba MG, et al. Adjuvant anastrozole versus exemestane versus letrozole, upfront or after 2 years of tamoxifen, in endocrine-sensitive breast cancer (FATA-GIM3): a randomised, phase 3 trial. *Lancet Oncol*. 2018:19(4):474-485
2009$	GBG 52 / ICE II Phase II	von Minckwitz G, Conrad B, Reimer T, Decker T, Eidtman H, Eiermann W, et al. A randomised phase 2 study comparing EC or CMF versus nab-paclitaxel plus capecitabine as adjuvant chemotherapy for nonfrail elderly patients with moderate to high-risk early breast cancer (ICE II-GBG 52). *Cancer*. 2015; 121:3639-3658.
2009J	NSABP B-46-I	Blum JL, Flynn PJ, Yothers G, Asmar L, Geyer CE, Jr., Jacobs SA, et al. Anthracyclines in early breast cancer: the ABC Trials--USOR 06-090, NSABP B-46-I/USOR 07132, and NSABP B-49 (NRG Oncology). *J Clin Oncol*. 2017; 35(23):2647-2655.
2009M	WSG Plan B	Nitz U, Gluz O, Clemens M, Malter WR, T., Nuding B, Aktas B, et al. West German Study PlanB trial: adjuvant four cycles of epirubicin and cyclophosphamide plus docetaxel versus six cycles of docetaxel and cyclophosphamide in HER2-negative early breast cancer. *J Clin Oncol*. 2019; 37(10):799-808.
2009X	TITAN / SCRA BRE145	Yardley DA, Arrowsmith ER, Daniel BR, Eakle J, Brufsky A, Drosick DR, et al. TITAN: phase III study of doxorubicin/cyclophosphamide followed by ixabepilone or paclitaxel in early-stage triple-negative breast cancer. *Breast Cancer Res Treat*. 2017; 164(3):649-658.

Figure S1: Changes in trial population over time

ER-positive disease **ER-negative disease**

(a) Nodal status (P<.0001 for trend in both groups)

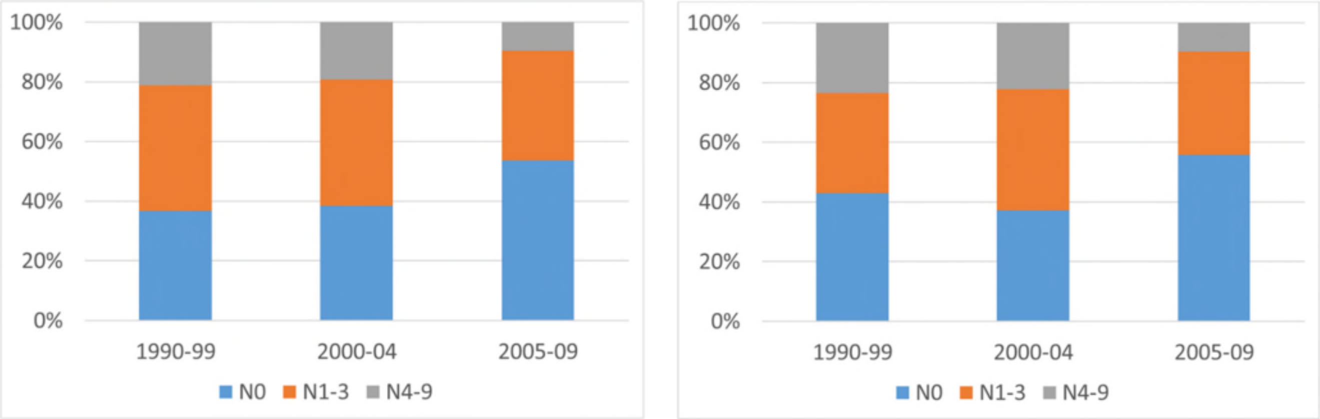

(b) Tumour size (P<.0001 ER-positive, p=0.02 ER-negative)

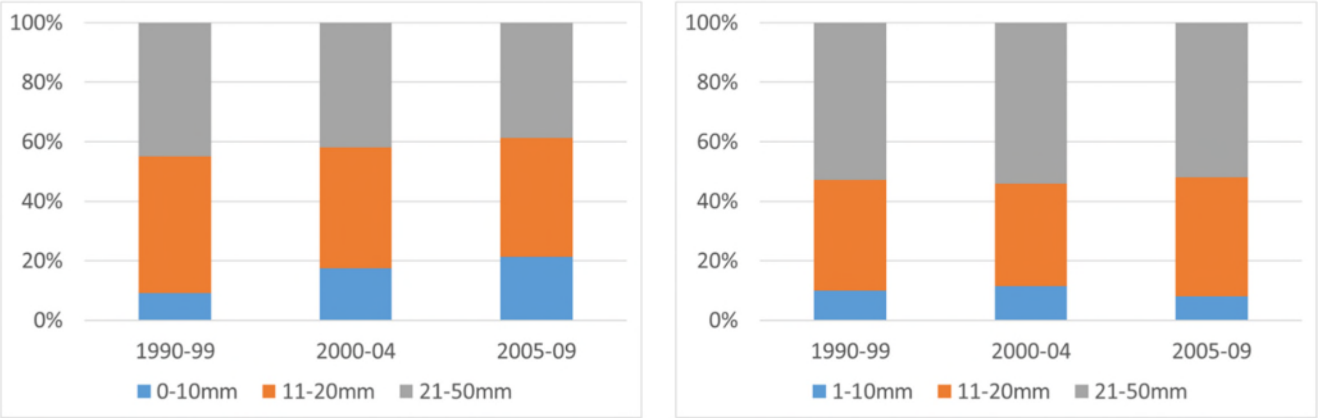

(c) TN status

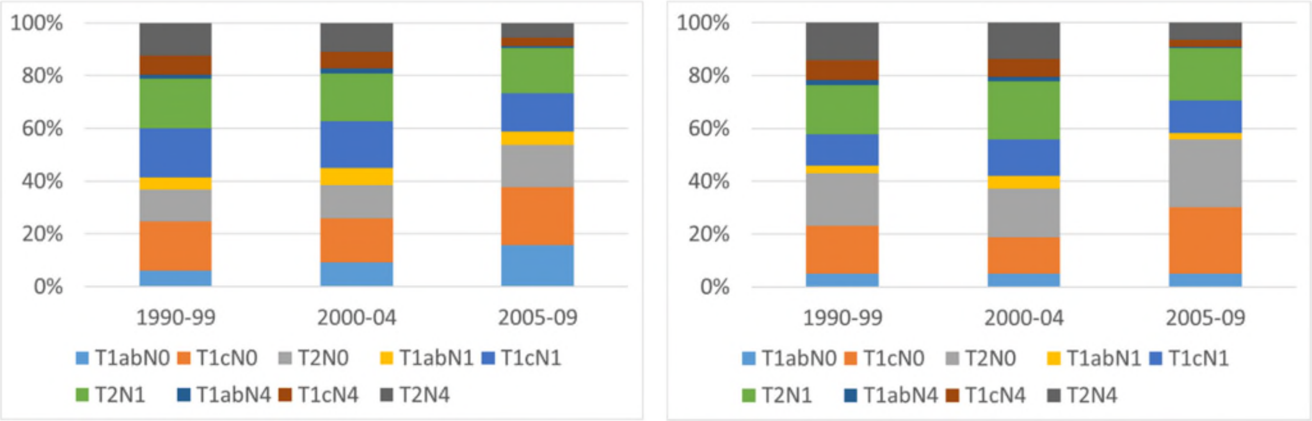

(d) Age (P<.0001 for younger age by time ER-postive, P<.0001 for older ER-negative)

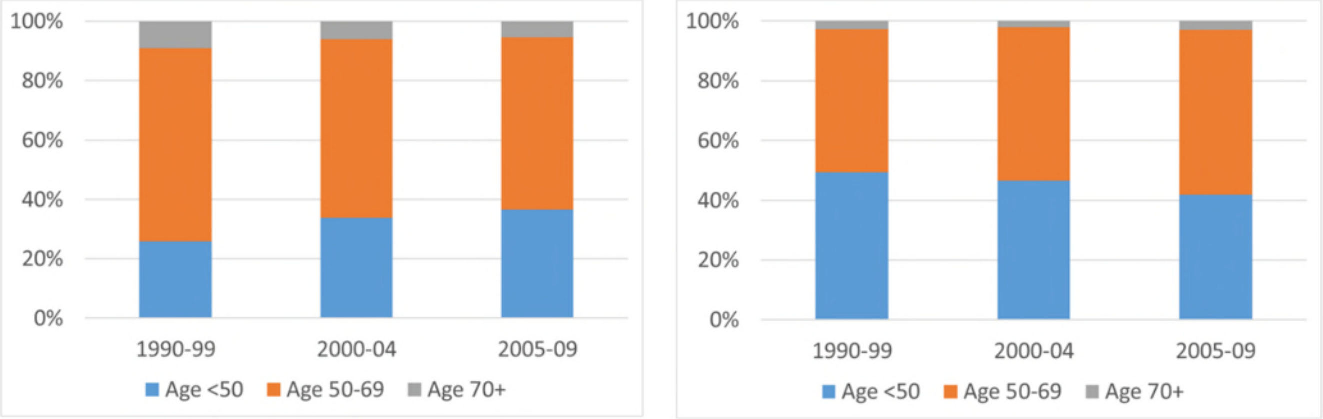

(e) Grade (P=.0001 ER-positive, P<.0001 ER-negative)

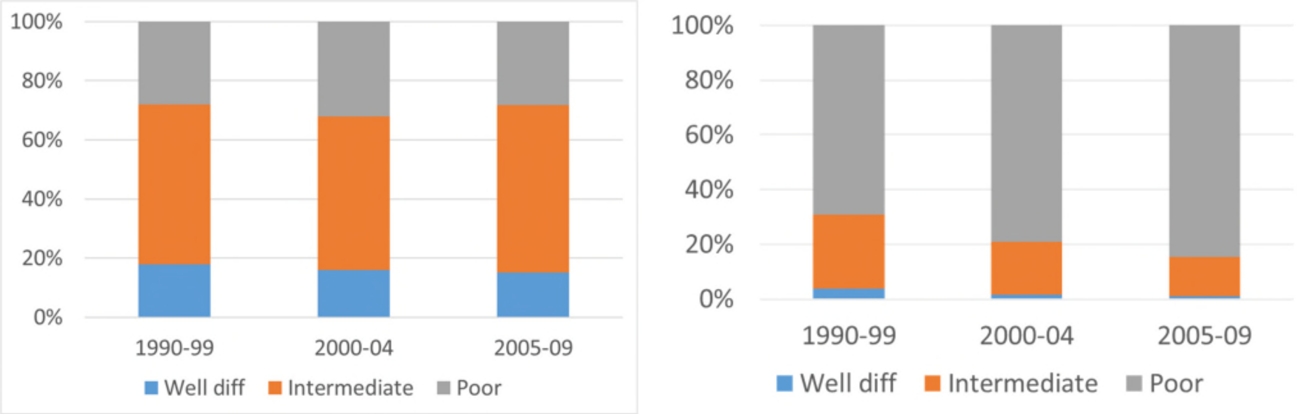

(f) HER2 Status

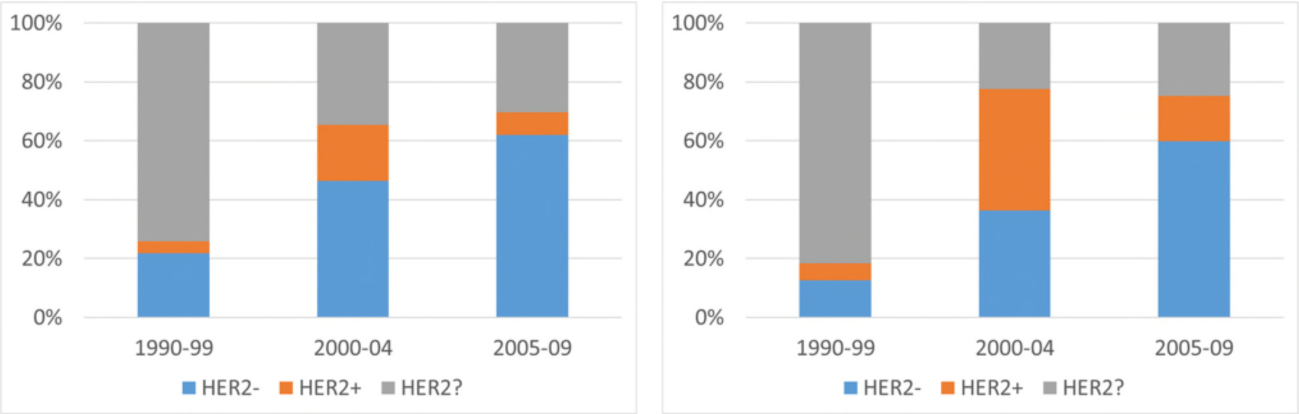

(g) Treatments

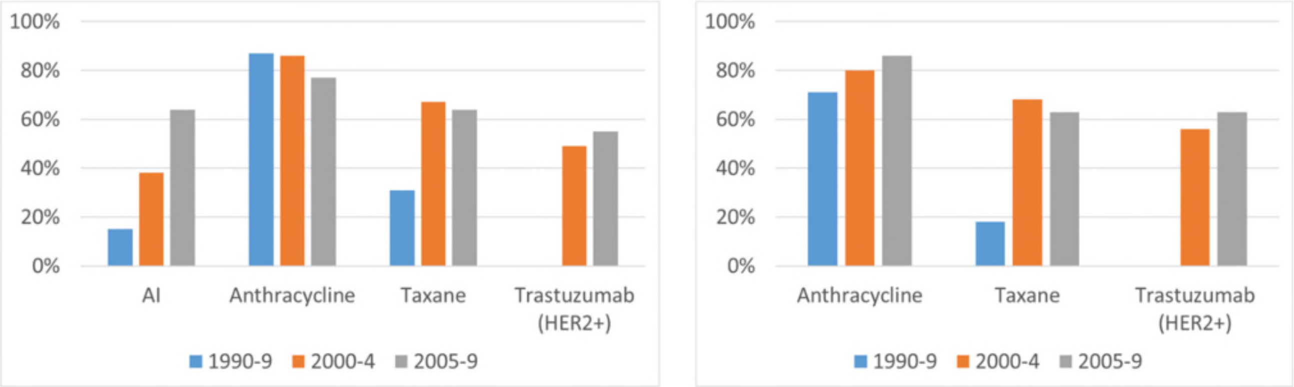

Figure S2: Risk of distant recurrence by period of enrolment overall and by nodal status. Interaction between rates in Figures 3a,b p<.0001.

Y-axis scale varies by number of positive nodes

ER-positive disease

(a) Unadjusted

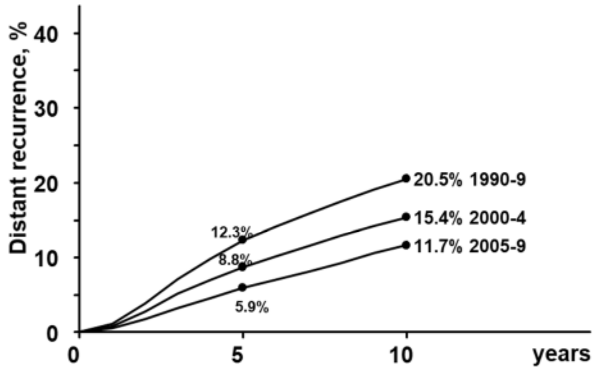

No. at risk (and, in each 5-year period, no. of events and annual rate)

1990-9	29080	(3458, 2.6%)	23509	(1865, 2.0%)	13014
2000-4	45495	(3770, 1.8%)	35101	(1662, 1.5%)	10563
2005-9	40236	(2116, 1.2%)	25271	(790, 1.2%)	2507

ER-negative disease

(b) Unadjusted

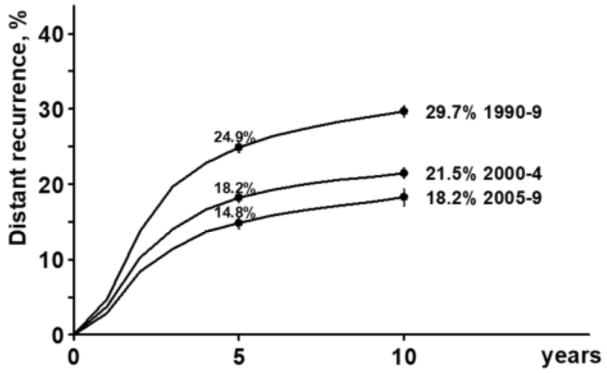

No. at risk (and, in each 5-year period, no. of events and annual rate)

1990-9	15925	(3857, 5.9%)	10526	(560, 1.4%)	6050
2000-4	16180	(2805, 4.1%)	11201	(354, 0.9%)	5000
2005-9	8830	(1222, 3.4%)	4902	(107, 0.9%)	432

(c) N0 disease

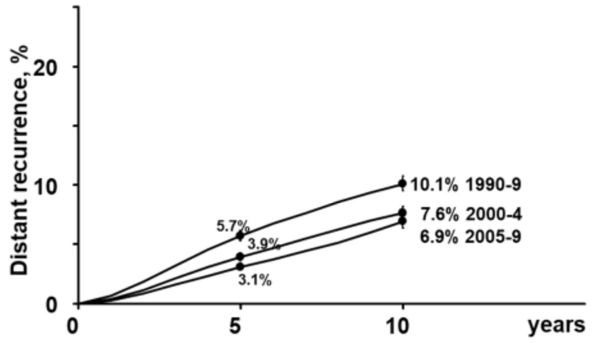

No. at risk (and, in each 5-year period, no. of events and annual rate)

1990-9	10673	(588, 1.2%)	9054	(351, 1.0%)	4992
2000-4	17465	(640, 0.8%)	13664	(288, 0.8%)	2542
2005-9	21597	(582, 0.6%)	12621	(258, 0.8%)	1332

(d) N0 disease

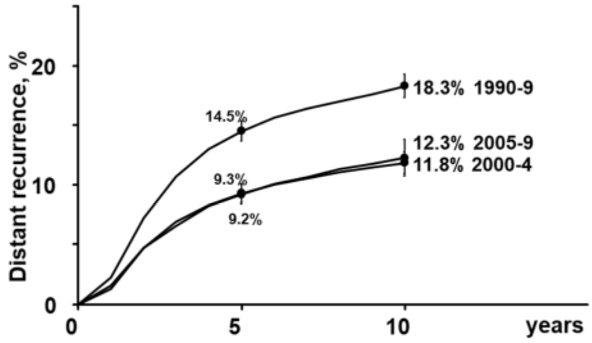

No. at risk (and, in each 5-year period, no. of events and annual rate)

1990-9	6841	(959, 3.2%)	5141	(192, 0.9%)	3125
2000-4	6025	(528, 2.0%)	4648	(100, 0.6%)	1878
2005-9	4918	(412, 2.0%)	2586	(44, 0.8%)	165

(e) N1-3 disease

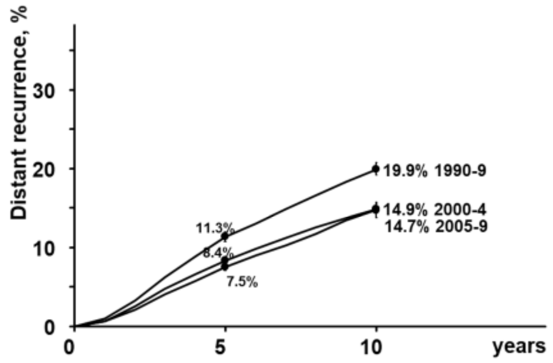

No. at risk (and, in each 5-year period, no. of events and annual rate)

1990-9	12285	(1356, 2.4%)	10173	(821, 2.0%)	5775
2000-4	19328	(1547, 1.7%)	15466	(790, 1.5%)	5846
2005-9	14799	(1021, 1.5%)	10162	(368, 1.6%)	894

(f) N1-3 disease

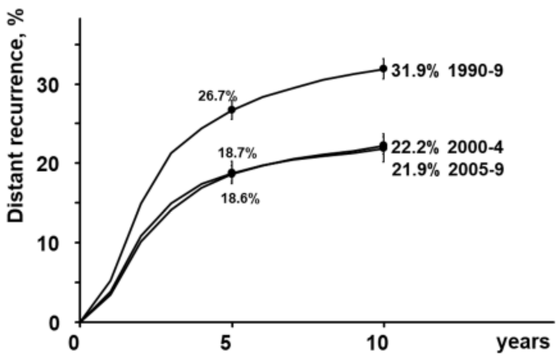

No. at risk (and, in each 5-year period, no. of events and annual rate)

1990-9	5329	(1397, 6.4%)	3595	(220, 1.5%)	2082
2000-4	6570	(1173, 4.2%)	4570	(155, 0.9%)	2126
2005-9	3068	(544, 4.3%)	1877	(42, 0.9%)	193

(g) N4-9 disease

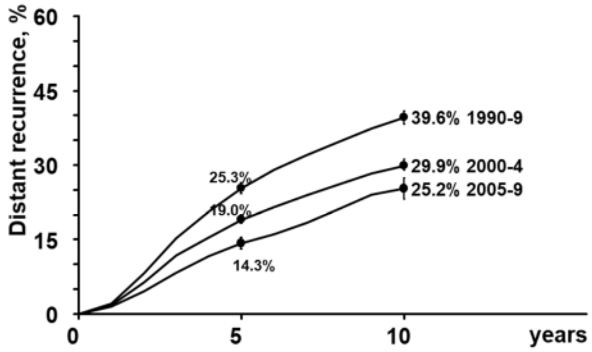

No. at risk (and, in each 5-year period, no. of events and annual rate)

1990-9	6122	(1514, 5.7%)	4282	(693, 4.3%)	2247
2000-4	8702	(1583, 4.2%)	5971	(584, 3.0%)	2175
2005-9	3840	(513, 3.0%)	2488	(164, 2.7%)	281

(h) N4-9 disease

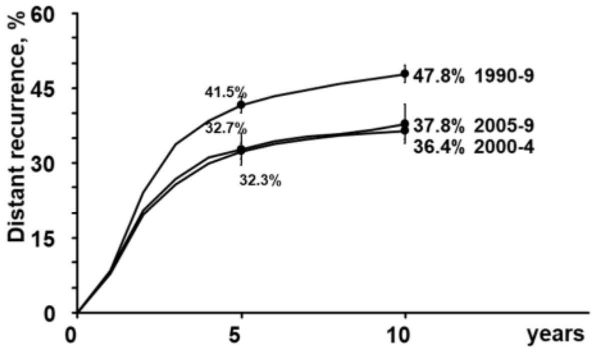

No. at risk (and, in each 5-year period, no. of events and annual rate)

1990-9	3755	(1501, 11.2%)	1790	(148, 2.4%)	843
2000-4	3585	(1104, 8.2%)	1983	(99, 1.4%)	996
2005-9	844	(266, 8.4%)	439	(21, 1.8%)	74

Figure S3. Improvements over time split by TN status – women with ER-positive tumours. Nodal status is denoted by the rows and tumour size by the columns, so that a T1c, N1-3 tumour is reflected in the 2nd row, 2nd column

Y-axis scale varies by TN status

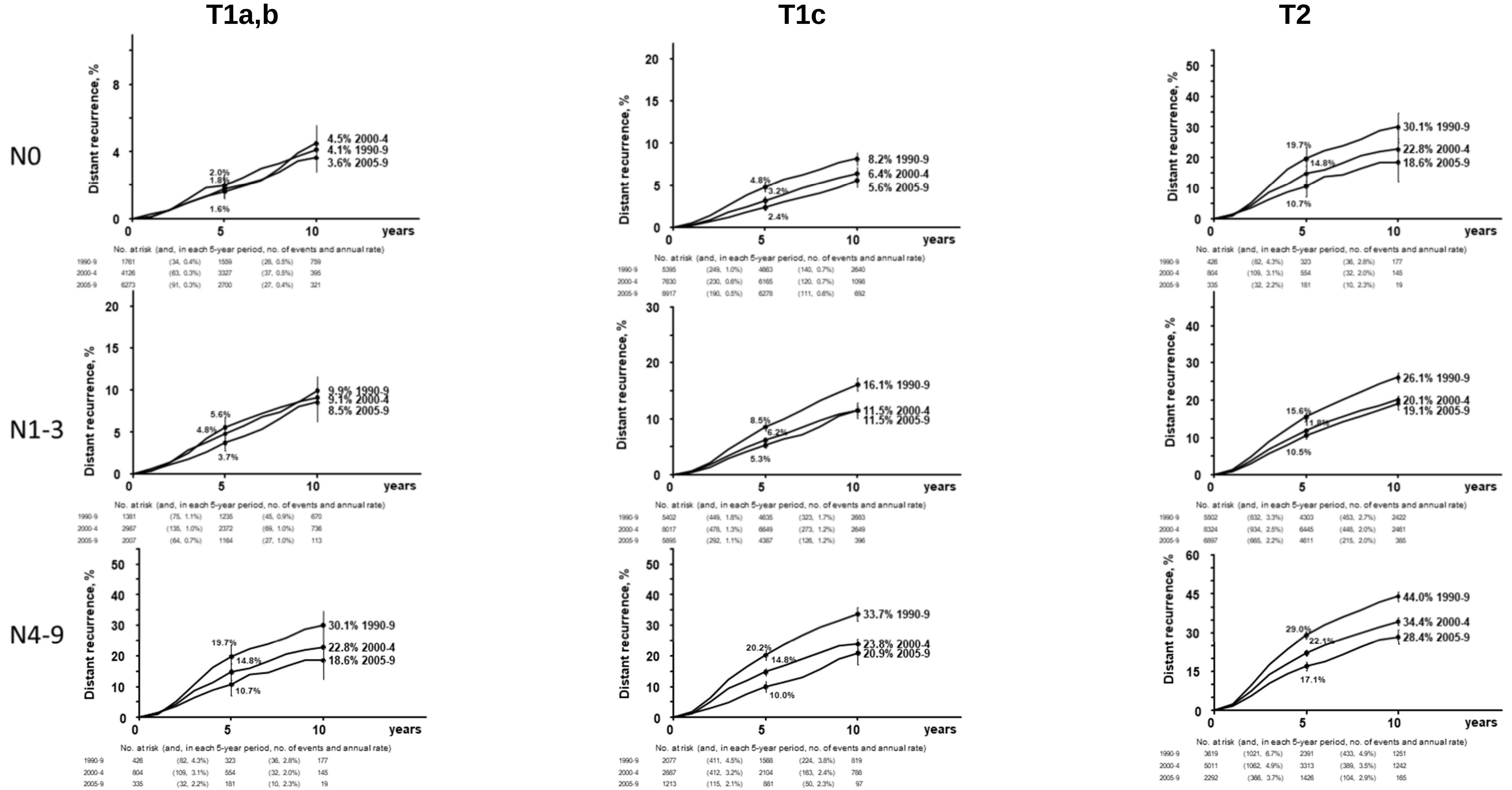

Figure S4. Improvements over time split by TN status – women with ER-negative tumours. Nodal status is denoted by the rows and tumour size by the columns, so that a T1c, N1-3 tumour is reflected in the 2nd row, 2nd column

Y-axis scale varies by TN status

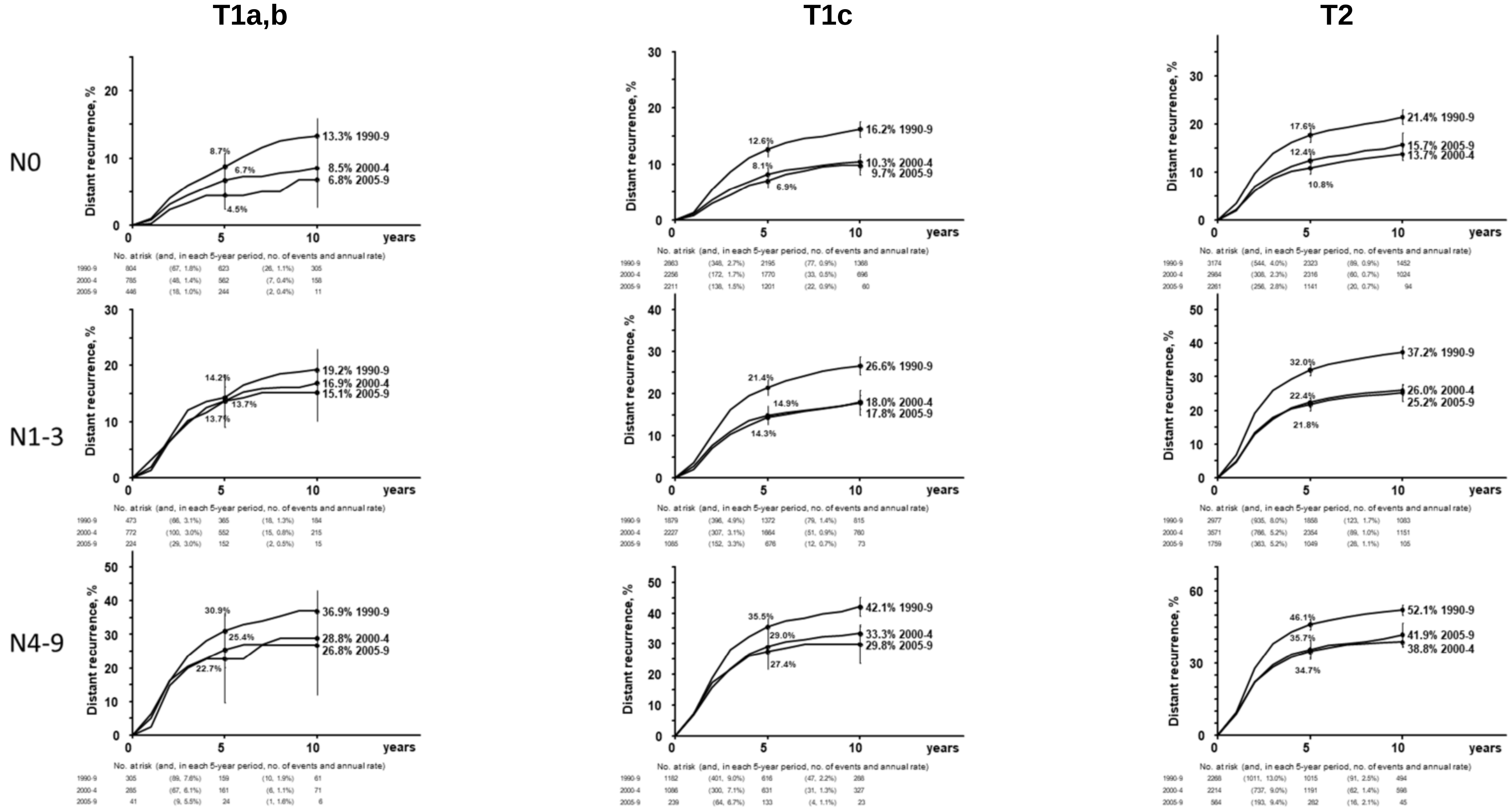

Figure S5. Distant recurrence by nodal status, excluding women with HER2-positive tumours

Y-axis scale varies by number of positive nodes

ER-positive N0

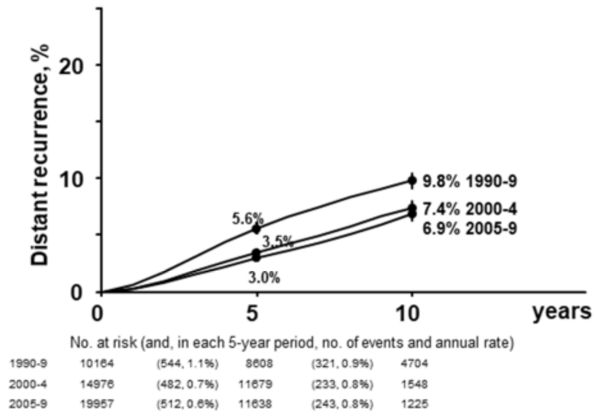

ER-positive N1-3

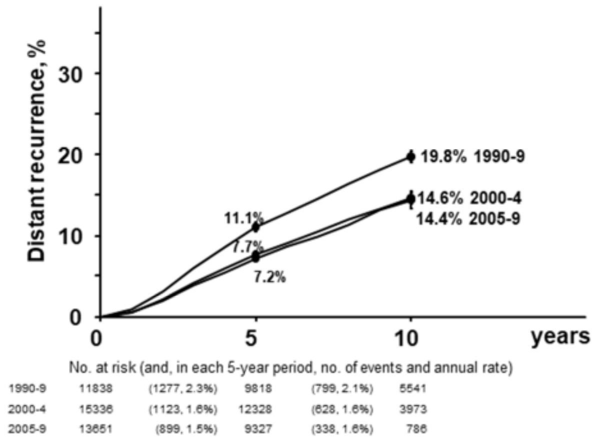

ER-positive N4-9

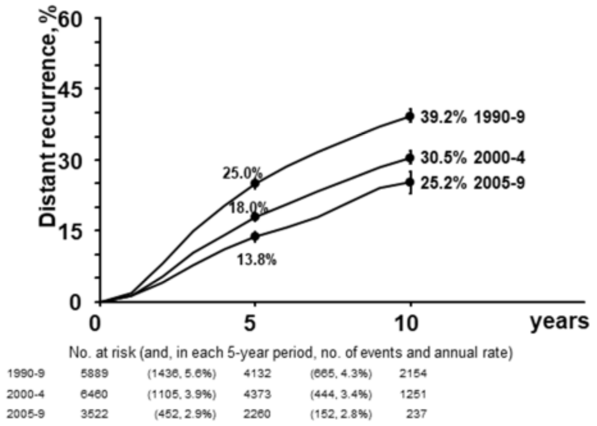

ER-negative N0

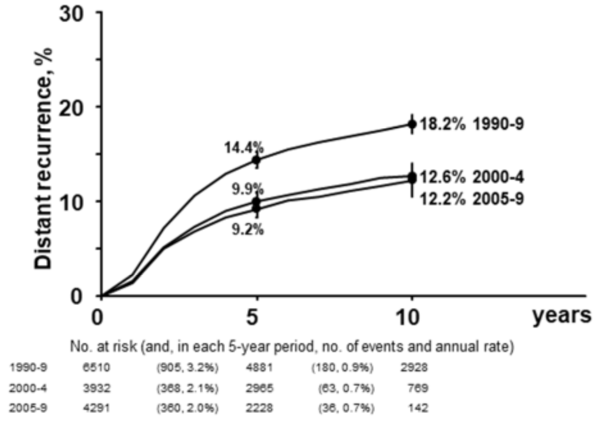

ER-negative N1-3

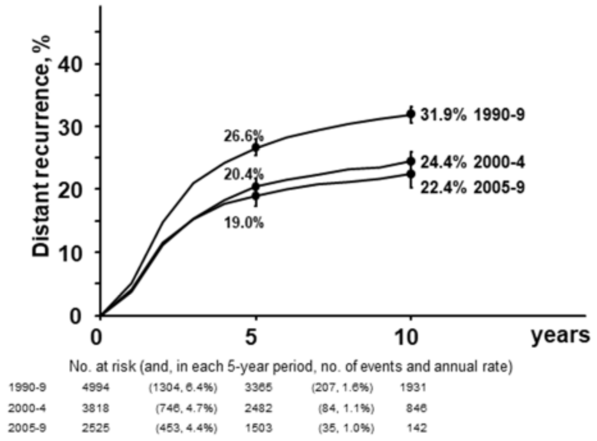

ER-negative N4-9

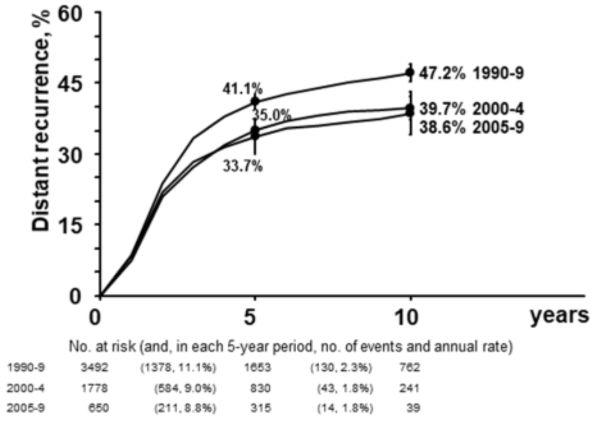

Figure S6. Effect of adjustment on rate ratios over time, excluding women with HER2-positive tumours

ER-positive, 5+ years of ET

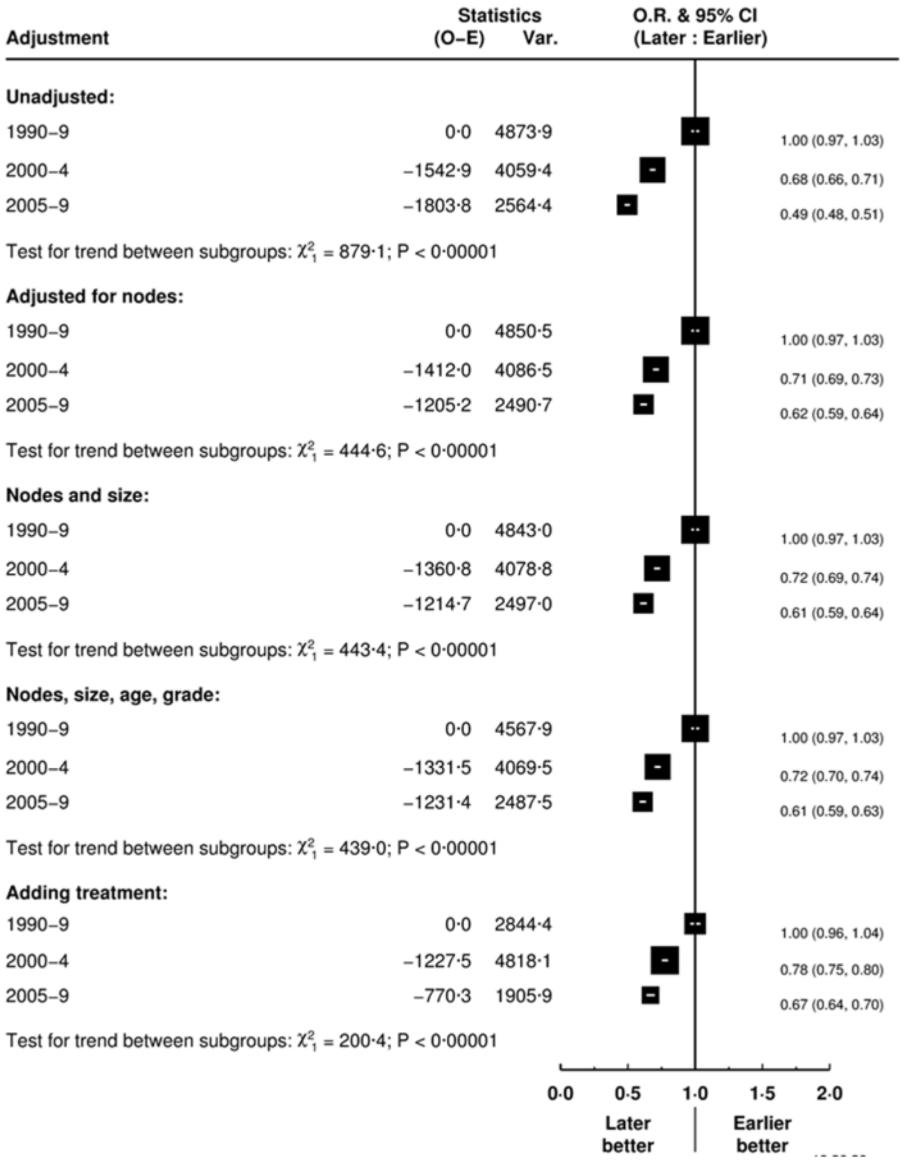

ER-negative

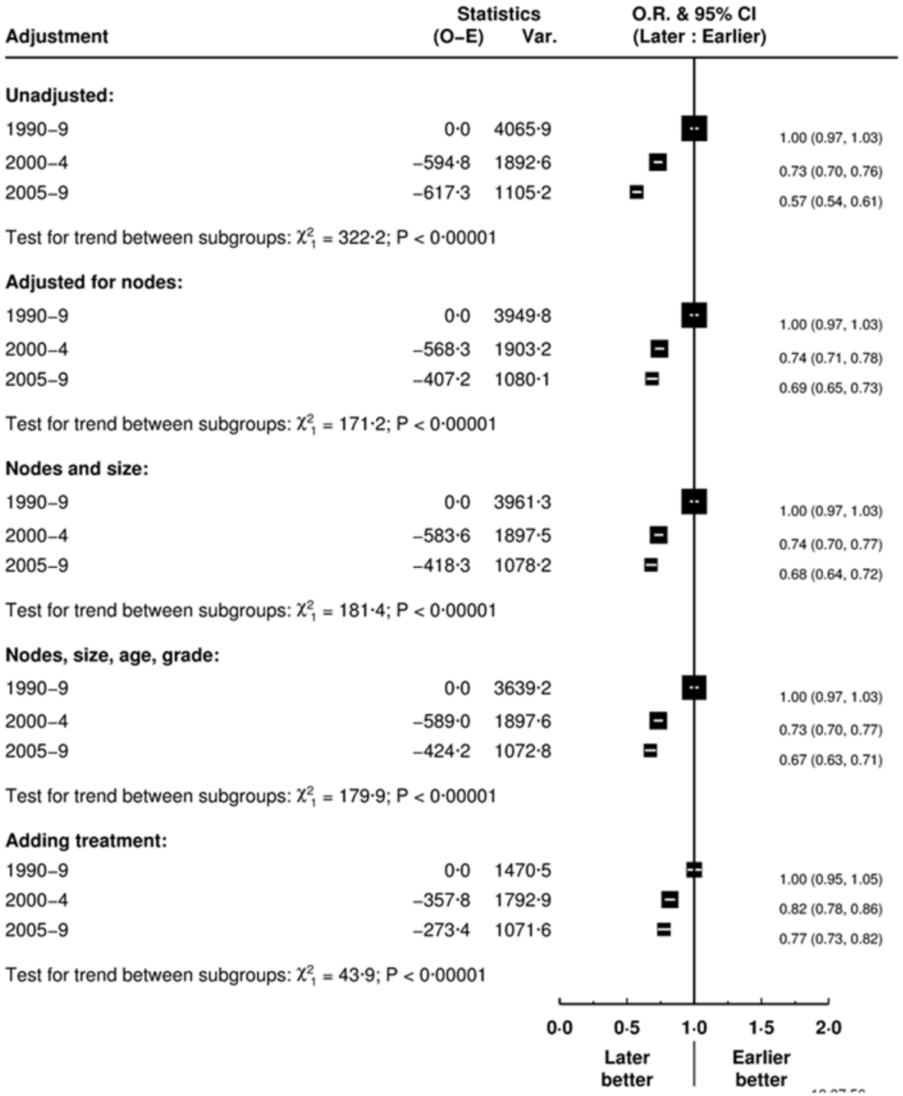

Figure S7. Distant recurrence by nodal status, women with confirmed HER2-negative tumours only

Y-axis scale varies by number of positive nodes

ER-positive N0

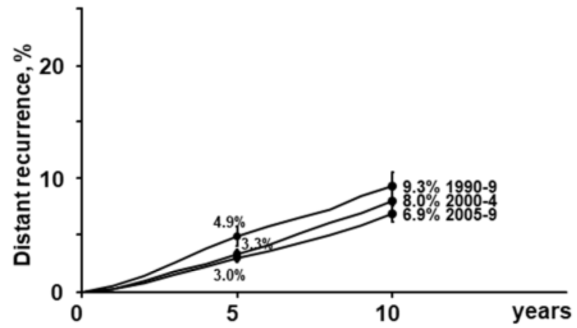

No. at risk (and, in each 5-year period, no. of events and annual rate)

1990-9	2654	(127, 1.0%)	2382	(96, 0.9%)	1544
2000-4	7371	(234, 0.7%)	6179	(154, 1.0%)	722
2005-9	14500	(387, 0.6%)	10253	(213, 0.8%)	1005

ER-positive N1-3

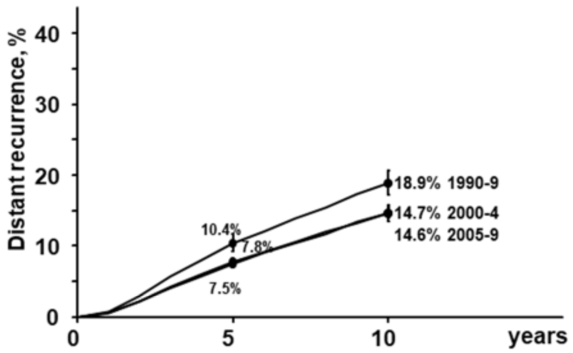

No. at risk (and, in each 5-year period, no. of events and annual rate)

1990-9	2368	(242, 2.2%)	2036	(166, 2.0%)	1205
2000-4	9848	(733, 1.6%)	8070	(453, 1.6%)	3239
2005-9	8675	(602, 1.5%)	6450	(250, 1.6%)	704

ER-positive N4-9

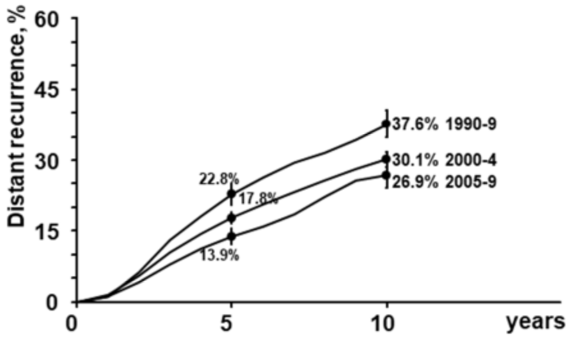

No. at risk (and, in each 5-year period, no. of events and annual rate)

1990-9	1298	(293, 5.1%)	966	(165, 4.3%)	557
2000-4	3841	(655, 3.9%)	2824	(327, 3.3%)	1102
2005-9	1786	(237, 3.0%)	1363	(118, 3.2%)	216

ER-negative N0

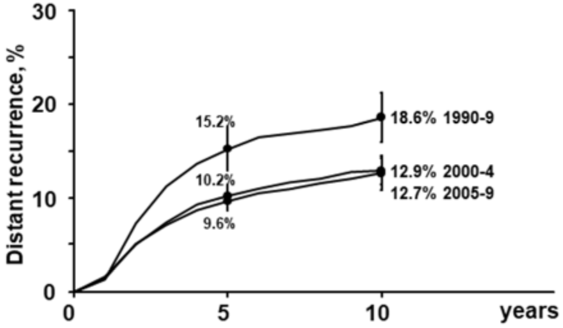

No. at risk (and, in each 5-year period, no. of events and annual rate)

1990-9	882	(133, 3.3%)	722	(26, 0.8%)	536
2000-4	2730	(262, 2.2%)	2024	(41, 0.7%)	490
2005-9	3562	(315, 2.1%)	2136	(35, 0.7%)	142

ER-negative N1-3

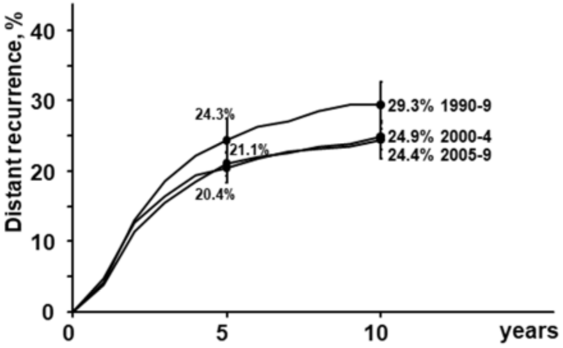

No. at risk (and, in each 5-year period, no. of events and annual rate)

1990-9	725	(175, 5.7%)	520	(32, 1.5%)	322
2000-4	2305	(463, 4.8%)	1557	(56, 1.0%)	708
2005-9	1429	(277, 4.8%)	905	(27, 1.2%)	117

ER-negative N4-9

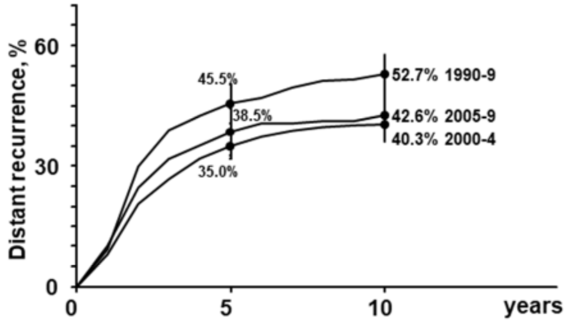

No. at risk (and, in each 5-year period, no. of events and annual rate)

1990-9	402	(180, 12.7%)	210	(26, 2.9%)	138
2000-4	853	(286, 8.9%)	480	(33, 2.0%)	202
2005-9	291	(108, 10.3%)	156	(7, 1.6%)	28

Figure S8. Effect of adjustment on rate ratios over time, women with confirmed HER2-negative tumours only

ER positive, 5+ years of ET

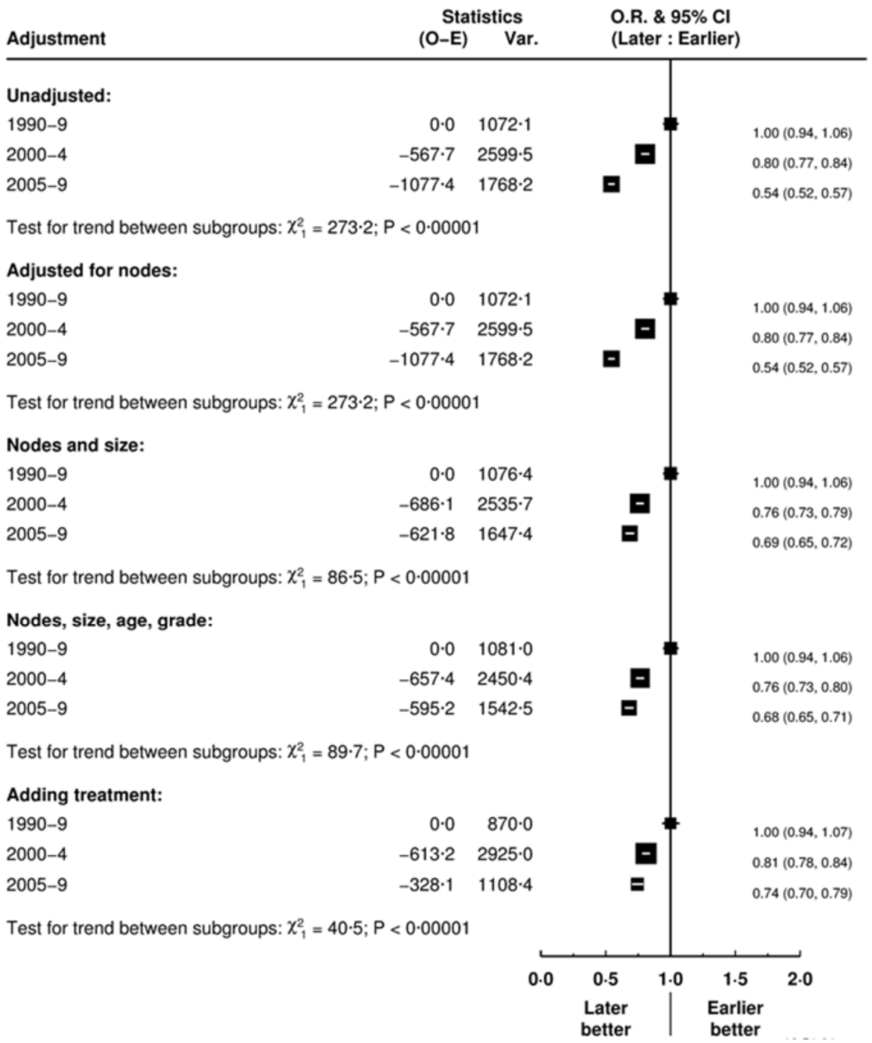

ER negative

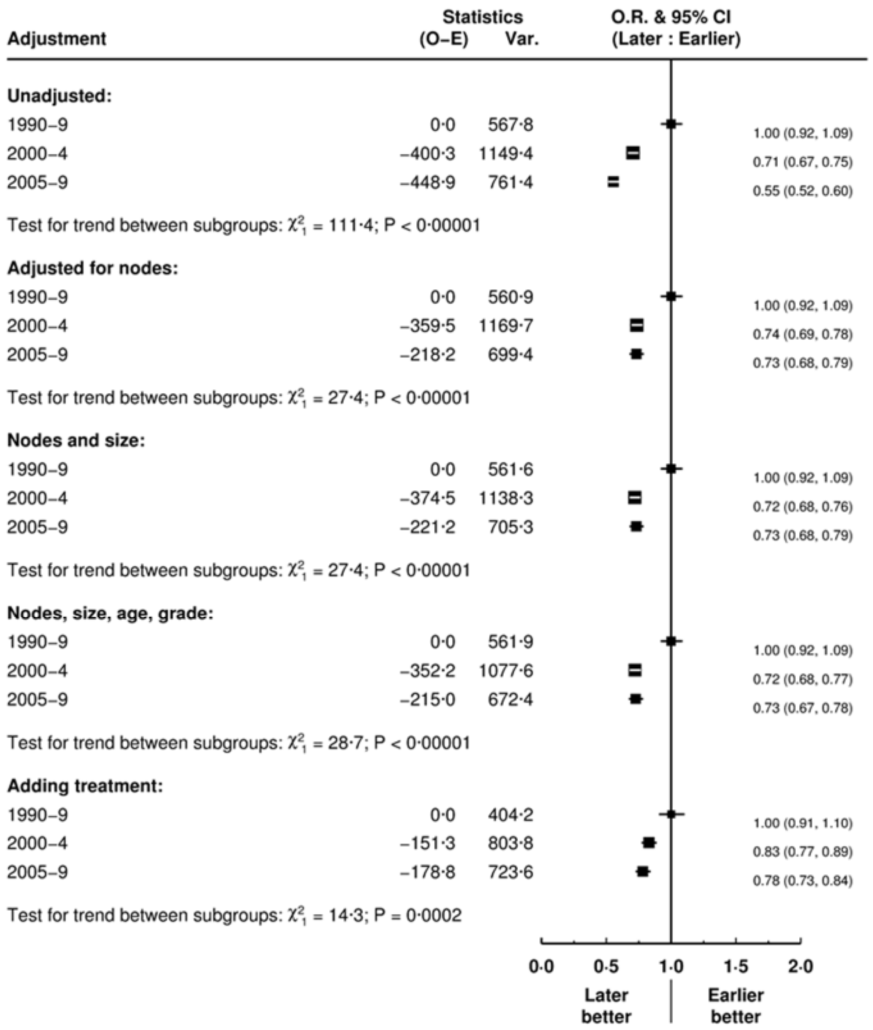

Figure S9. Distant recurrence by nodal status, women with confirmed HER2-positive tumours only

Y-axis scale varies by number of positive nodes

ER-positive N0

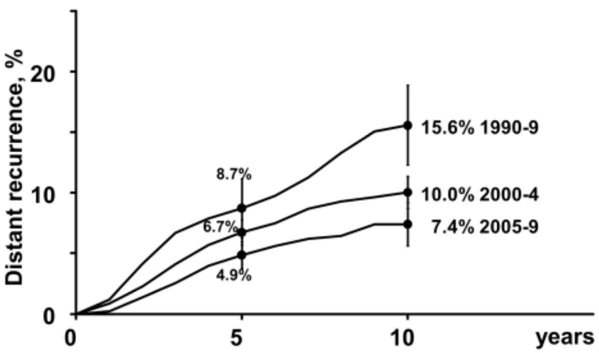

No. at risk (and, in each 5-year period, no. of events and annual rate)

1990-9	509	(44, 1.8%)	446	(30, 1.6%)	288
2000-4	2489	(158, 1.4%)	1985	(55, 0.8%)	994
2005-9	1640	(70, 1.0%)	983	(15, 0.6%)	118

ER-positive N1-3

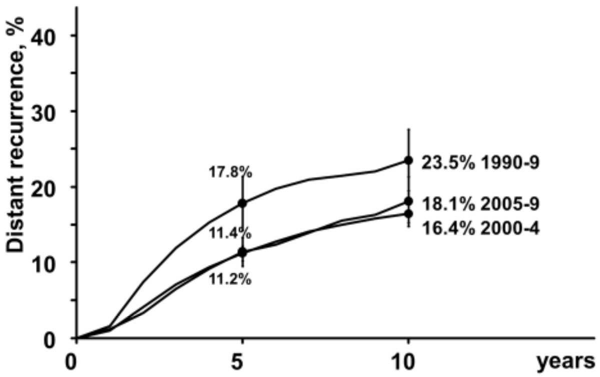

No. at risk (and, in each 5-year period, no. of events and annual rate)

1990-9	447	(79, 3.9%)	355	(22, 1.5%)	234
2000-4	3992	(424, 2.4%)	3138	(162, 1.3%)	1873
2005-9	1148	(122, 2.4%)	835	(30, 1.4%)	110

ER-positive N4-9

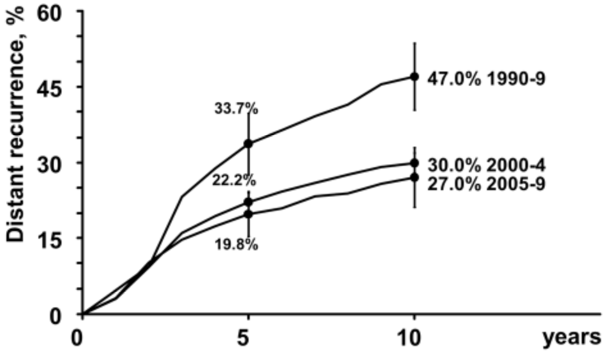

No. at risk (and, in each 5-year period, no. of events and annual rate)

1990-9	233	(78, 8.0%)	150	(28, 4.5%)	93
2000-4	2242	(478, 5.0%)	1598	(140, 2.2%)	924
2005-9	318	(61, 4.5%)	228	(12, 1.8%)	44

ER-negative N0

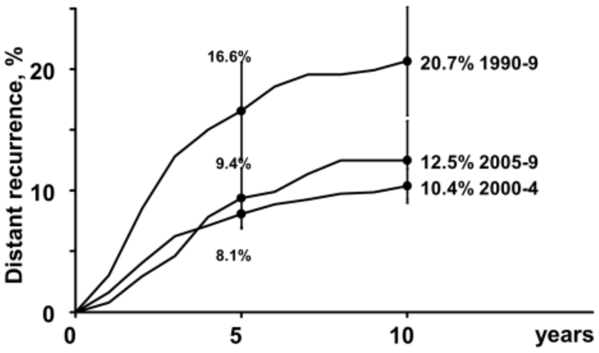

No. at risk (and, in each 5-year period, no. of events and annual rate)

1990-9	331	(54, 3.7%)	260	(12, 1.0%)	197
2000-4	2093	(160, 1.7%)	1683	(37, 0.5%)	1109
2005-9	627	(52, 2.0%)	358	(8, 1.0%)	23

ER-negative N1-3

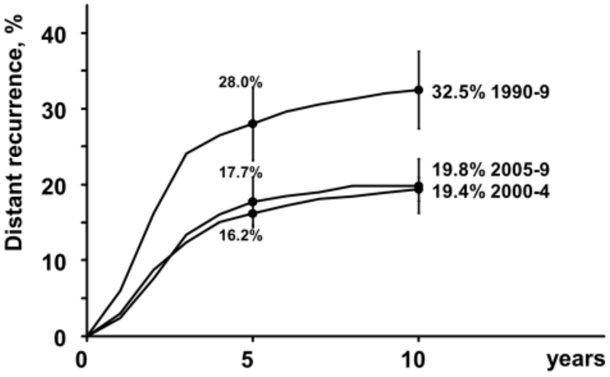

No. at risk (and, in each 5-year period, no. of events and annual rate)

1990-9	335	(93, 6.8%)	230	(13, 1.3%)	151
2000-4	2752	(427, 3.6%)	2088	(71, 0.8%)	1280
2005-9	543	(91, 4.0%)	374	(7, 0.7%)	51

ER-negative N4-9

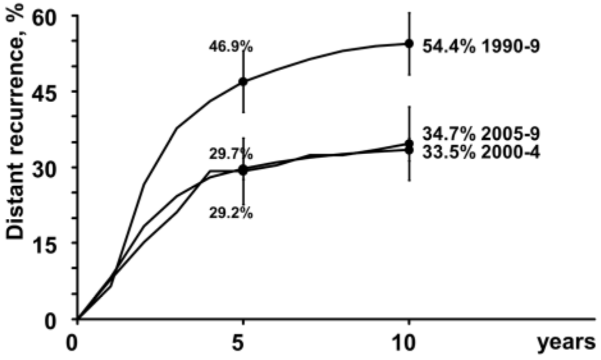

No. at risk (and, in each 5-year period, no. of events and annual rate)

1990-9	263	(123, 12.9%)	137	(18, 3.2%)	81
2000-4	1807	(520, 7.4%)	1153	(56, 1.1%)	755
2005-9	194	(55, 7.1%)	124	(7, 1.7%)	35

Figure S10. Effect of adjustment on rate ratios over time, women with ER-positive tumours, 5 years of endocrine therapy scheduled

ER positive, 5 years of ET

Adjustment	Statistics (O–E)	Var.	O.R. & 95% CI (Later : Earlier)
**Unadjusted:**			
1990–9	0·0	5180·4	1.00 (0.97, 1.03)
2000–4	–1674·0	4874·0	0.71 (0.69, 0.73)
2005–9	–1216·9	2067·7	0.56 (0.53, 0.58)
Test for trend between subgroups: χ^2_1 = 595·3; P < 0·00001			
**Adjusted for nodes:**			
1990–9	0·0	5196·6	1.00 (0.97, 1.03)
2000–4	–1640·6	4883·4	0.71 (0.69, 0.73)
2005–9	–717·2	2008·8	0.70 (0.67, 0.73)
Test for trend between subgroups: χ^2_1 = 281·7; P < 0·00001			
**Nodes and size:**			
1990–9	0·0	5160·7	1.00 (0.97, 1.03)
2000–4	–1614·5	4899·9	0.72 (0.70, 0.74)
2005–9	–719·7	2008·6	0.70 (0.67, 0.73)
Test for trend between subgroups: χ^2_1 = 278·4; P < 0·00001			
**Nodes, size, age, grade:**			
1990–9	0·0	4568·2	1.00 (0.97, 1.03)
2000–4	–1771·4	5113·0	0.71 (0.69, 0.73)
2005–9	–752·1	1950·9	0.68 (0.65, 0.71)
Test for trend between subgroups: χ^2_1 = 293·8; P < 0·00001			
**Adding treatment:**			
1990–9	0·0	2482·0	1.00 (0.96, 1.04)
2000–4	–1540·2	5892·4	0.77 (0.75, 0.79)
2005–9	–427·9	1477·5	0.75 (0.71, 0.79)
Test for trend between subgroups: χ^2_1 = 102·4; P < 0·00001			

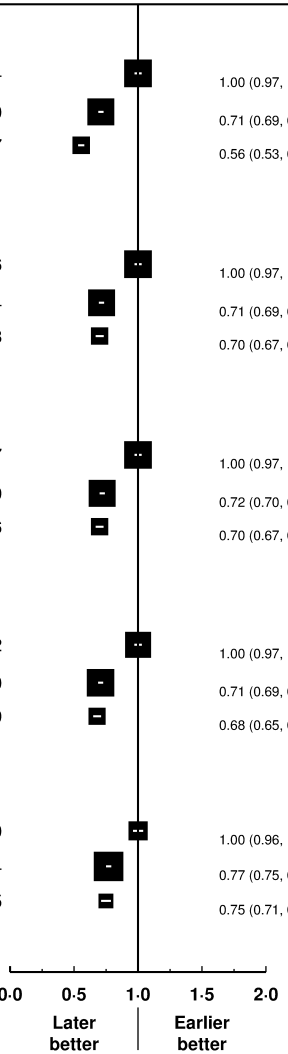

Supplement: Supplementary appendix [file mmc1.pdf]
